# Neuronal detection of social actions directs collective escape behaviour

**DOI:** 10.1038/s41586-026-11041-1

**Published:** 2026-09-23

**Authors:** Jo-Hsien Yu, Geoff T. Meyerhof, Jimjohn Milan, Julia L. Napoli, Matthew Lovett-Barron

**Affiliations:** Department of Neurobiology, School of Biological Sciences. University of California, San Diego, La Jolla, CA USA

**Keywords:** Social behaviour, Motion detection, Cooperation, Animal behaviour

## Abstract

Animals in groups obtain information from social partners to engage in adaptive behaviour^1–3^. Social information transmission is observed in fish schools^4–6^, bird flocks^7,8^ and human groups^9,10^, but the neural representation of such socially acquired information is poorly understood^3,11,12^. Here we show that, in the schooling glassfish *Danionella cerebrum*^13–15^, collective escape from danger can be mediated by an individual’s visual perception of other escaping animals. To understand the neural basis of socially transmitted escape behaviour, we imaged neural activity from adult glassfish viewing the actions of virtual conspecifics. Visual neurons in the midbrain optic tectum^16,17^ and thalamus^18^ increased their activity when virtual conspecifics escaped. Escape-responsive neurons also responded to the sudden disappearance of virtual fish, yet were unaffected by the disappearance of stimuli moving with non-biological linear motion. Behaviourally, fish retreated from virtual schools that escaped or disappeared, but only those swimming with biological burst-and-glide motion. Neural encoding of this rapid social offset allows fish to infer danger from social information alone, a potentially effective strategy for animals that are capable of rapid movement but have a limited visual range^14,19^. These results show how the neural computations of individuals enable rapid information sharing in collectives.

## Abstract (web summary)

Collective escape in *Danionella cerebrum* arises from visual detection of rapid, biologically realistic motion of other fish, with midbrain and thalamic neurons encoding social offsets that enable fish to infer danger from others’ behaviour.

## Main

Living in groups provides animals with a variety of advantages, including protection from danger and predation^1–8^. In fish schools and bird flocks, an approaching predator or other threatening stimulus can drive collective escape and avoidance behaviours^5,6,20–24^. Individuals in these groups need not directly sense the threat to avoid danger if they act in response to the movements of their threat-informed neighbours, a benefit of collective living referred to as the ‘many eyes effect’^25,26^. Despite the prevalence of collective movement and predator avoidance in nature^1–3,22,27^, little is known about the neural mechanisms that allow individuals to obtain relevant, actionable information from their partners^3,11,12^. The neural basis of social recognition has been characterized in a variety of invertebrate and vertebrate species^15,18,28–31^, but it remains unclear how individuals recognize the specific actions of their social partners to engage in adaptive behaviour that benefits the individual and the group.

We address this question by studying the brain and behaviour of *D. cerebrum*, a micro glassfish that engages in collective schooling behaviour using visual perception of social partners^13,15,32^ and that is amenable to large-scale in vivo neural activity imaging^15,33–35^. This model system provides us with experimental access to the sensory cues and neural circuits that underlie social information transmission during collective responses to threats. Here we examine the perceptual and neural basis of collective escape behaviours in adult *D. cerebrum* and identify a neural signature of social action detection in central visual circuits that engages escape behaviour in observers to produce collective threat avoidance.

## Collective escape of *Danionella* groups

**Fig. 1:**
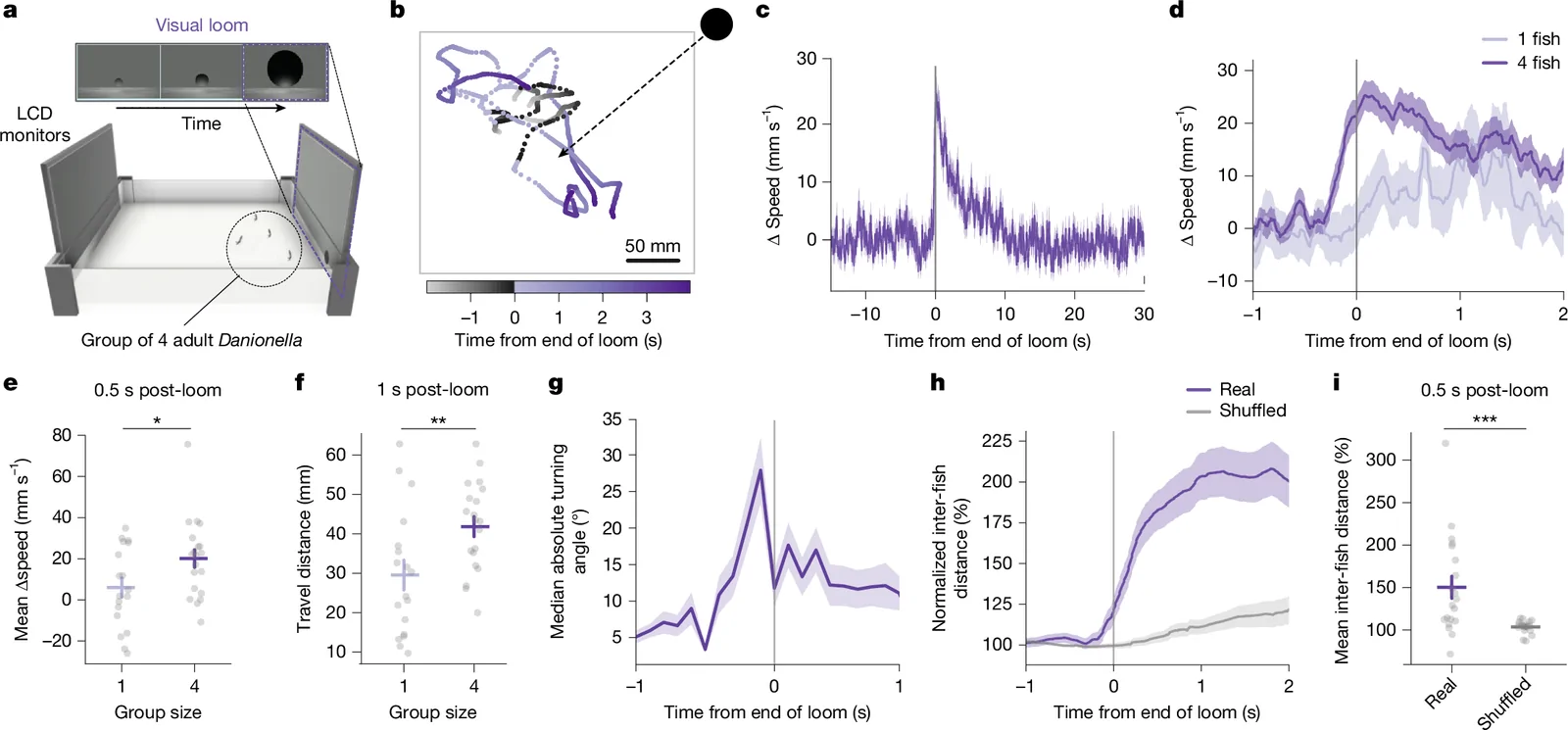
Collective escape behaviour of *D. cerebrum* groups. **a**, Behaviour paradigm for vision-driven escape. Top, sequence of looming sphere on LCD monitors. **b**, Example fish trajectories in a group of 4 fish over 6 s. Position or each fish is coloured by time from the end of the loom. Note the increased distance in position before the stimulus ends. The virtual path of the looming stimulus projection is marked with a dashed arrow. **c**, Change in speed at loom offset. *n* = 20 sessions from 4× groups of 4 fish. Note the punctate escape response in this longer time series. **d**, Same as **c**, but in the 3 s around loom offset. Speed of the single fish and the average speed of the four fish in a group was used for each trial. *n* = 18 (individual fish) and 20 (4-fish group) sessions. **e**, Change of speed in 0.5 s after loom offset. *n* = 18 and 20 sessions. Each dot is a single fish in each trial (chosen at random from groups of 4). **f**, Travel distance in 1 s after loom offset. *n* = 18 and 20 sessions. Each dot is a single fish or the average all fish in each trial. **g**, Median turning angle for all fish in groups of 4 at the end of loom. *n* = 80 fish from 20 sessions. Note the increase of large turning angle before the end of the loom. **h**, Inter-fish distance around loom offset, normalized with the average of 5 s before loom offset. Time-shuffled distances are present in grey. *n* = 20 sessions. **i**, Mean inter-fish distance in 0.5 s after loom offset. *n* = 20 groups. Each dot is the average distance of all pairs in each trial. All data were from 6 single fish (2–4 sessions each) and 4 4-fish groups (5 sessions each), and are shown as mean ± s.e.m. See Supplementary Table 1 for full statistics and sample sizes. *P < 0.05, **P < 0.01, ***P < 0.001. Source data

**Extended Data Fig. 1:**
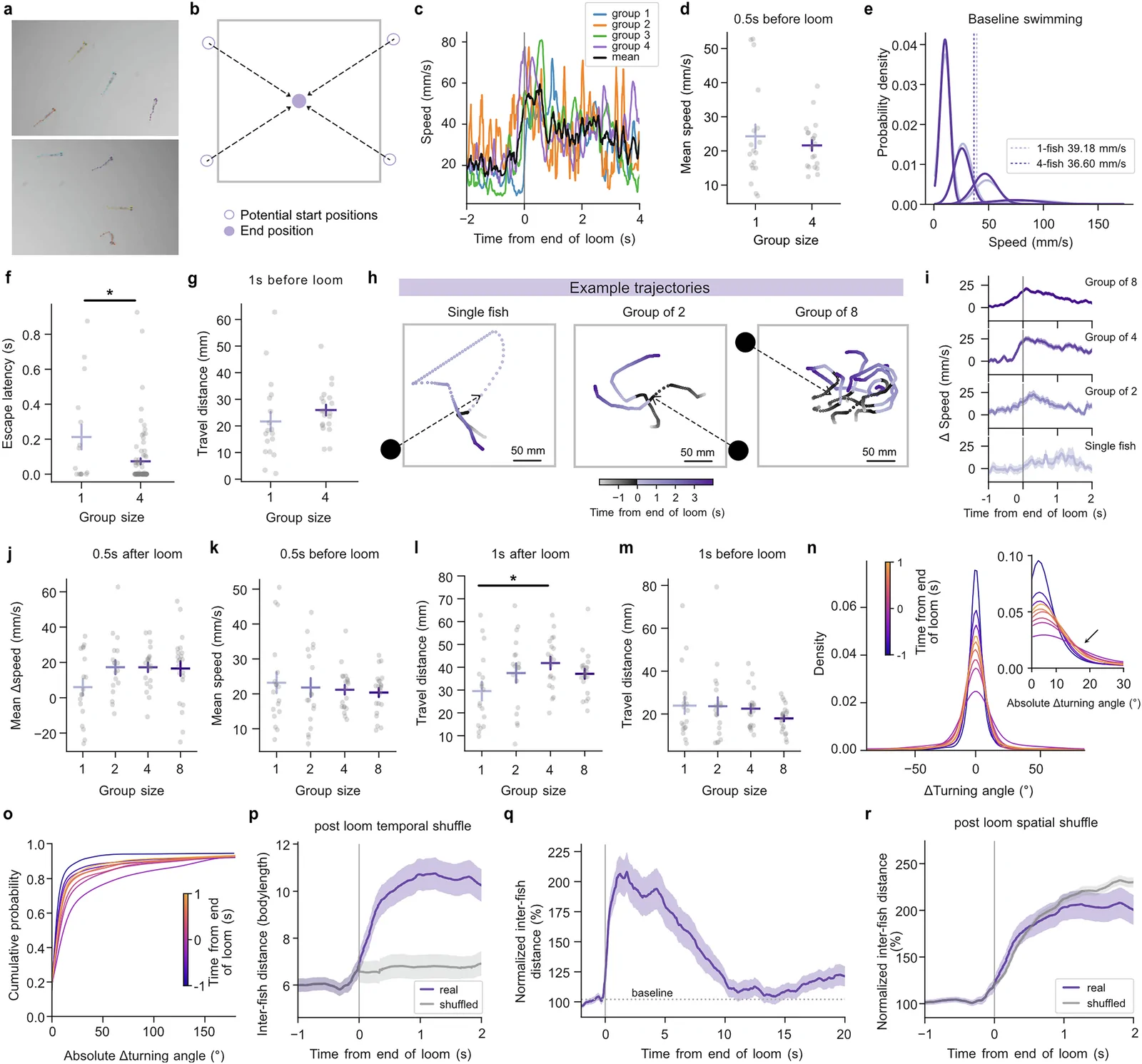
Visual loom-driven escape behaviour of *D. cerebrum* groups. **a** Example frames with SLEAP inference overlaid for multi-animal tracking. **b** Schematics of randomized loom stimulus approach trajectories. **c** Example speed from 4 four-fish groups around loom offset. **d** Baseline speed in the 0.5 sec before loom offset. n = 18 and 20 sessions. Each dot is a single fish or the average all fish in each trial. **e** Probability density function (PDF) of baseline speed before loom onset. Vertical dashed lines mark the threshold for low vs high speed states for each group size. **f** Escape latency. n = 15 and 80 sessions (fish reached high speed within 1 sec). Each dot is a fish in a trial. **g** Baseline travel distance in the 1 sec before loom offset. n = 18 and 20 sessions. Each dot is a single fish or the average of all fish in each trial. **h** Example fish trajectories over 6 sec. Position of each fish is colored by time from the end of the loom. The virtual path of the looming stimulus projection is marked with a dashed arrow. **i-k** Speed of different group sizes: (**j**) change of speed in the 0.5 sec after and (**k**) baseline speed in the 0.5 sec before loom offset. n = 18, 18, 20, 22 sessions. Each dot is a single fish or the average of all fish in each trial. **l**,**m** Travel distance (**l**) in the 1 sec after and (**m**) in the 1 sec before loom offset. n = 18, 18, 20, 22 sessions. Each dot is a single fish or the average of all fish in each trial. **n**,**o** (**n**) Probability density function (PDF) and (**o**) Cumulative distribution function (CDF) of turning angle in 250 ms bins for fish in a group of four. Inset in (**n**) shows the PDFs between 0° to 30°. Note the increase of large angles 250 ms before loom offset (arrow). n = 20 sessions from 80 fish. **p-r** Inter-fish distance at loom offset for four-fish groups. Gray line in (**p**) is the distance when fish position is time-shuffled post loom. (**q**) Normalized inter-fish distance over 20 sec at loom offset. Note the recovery of inter-fish distance to baseline at ~10 s post-stimulus. (**r**) Normalized inter-fish distance at loom offset. Gray line is the distance when fish position is identity-shuffled post loom. n = 20 groups. *p < 0.05, **p < 0.01, ***p < 0.001. All data was from 10 single fish, 3 two-fish groups, 5 four-fish groups, and 6 eight-fish groups (1–5 sessions each), and shown in mean ± SEM. See Supplementary Table 1 for full statistics and sample sizes. Source data

We first characterized the escape behaviour of adult *D. cerebrum* in groups of four by measuring the positions and postures of fish responding to a looming visual object, which mimics an approaching predator (Extended Data Fig. 1a,b and Methods). Visual looming stimuli drive robust escape and defensive behaviours in aquatic and terrestrial animals^36^. We built a 265 mm×265 mm square arena to deliver directional looming stimuli to groups of *D. cerebrum*, in which two of the four walls were lined with a video screen displaying a textured floor and dim sky (Fig. 1a). Looming stimuli were rendered as dark spheres moving towards the centre of the arena from one of four randomly selected directions (3–6 s in duration, delivered every 7 min; Methods). These stimuli elicited a short-latency escape behaviour (Fig. 1b,c and Extended Data Fig. 1c). We investigated how the presence of social partners may inform escape behaviour by comparing the behaviour of single fish to fish in groups of four. Single fish escaped by increasing their speed by 6.0 ± 4.6 mm s^−1^ in the 0.5 s after loom offset (*n* = 18), whereas a randomly chosen fish in a group of 4 increased their speed by 17.3 ± 2.7 mm s^−1^ (*n* = 20). Compared to single fish, fish in groups showed a greater increase in speed (Fig. 1d,e and Extended Data Fig. 1d–f) and escaped by a greater distance (Fig. 1f and Extended Data Fig. 1g). We observed these escape behaviours in groups of different sizes, but not in single fish (Extended Data Fig. 1h–m). Escaping fish also rapidly increased the magnitude of their turning, but their turns were directionally unbiased^37^ (Fig. 1g; Extended Data Fig. 1n,o). These results demonstrate that *D. cerebrum* collectively escape in response to visual looming stimuli.

**Extended Data Fig. 2:**
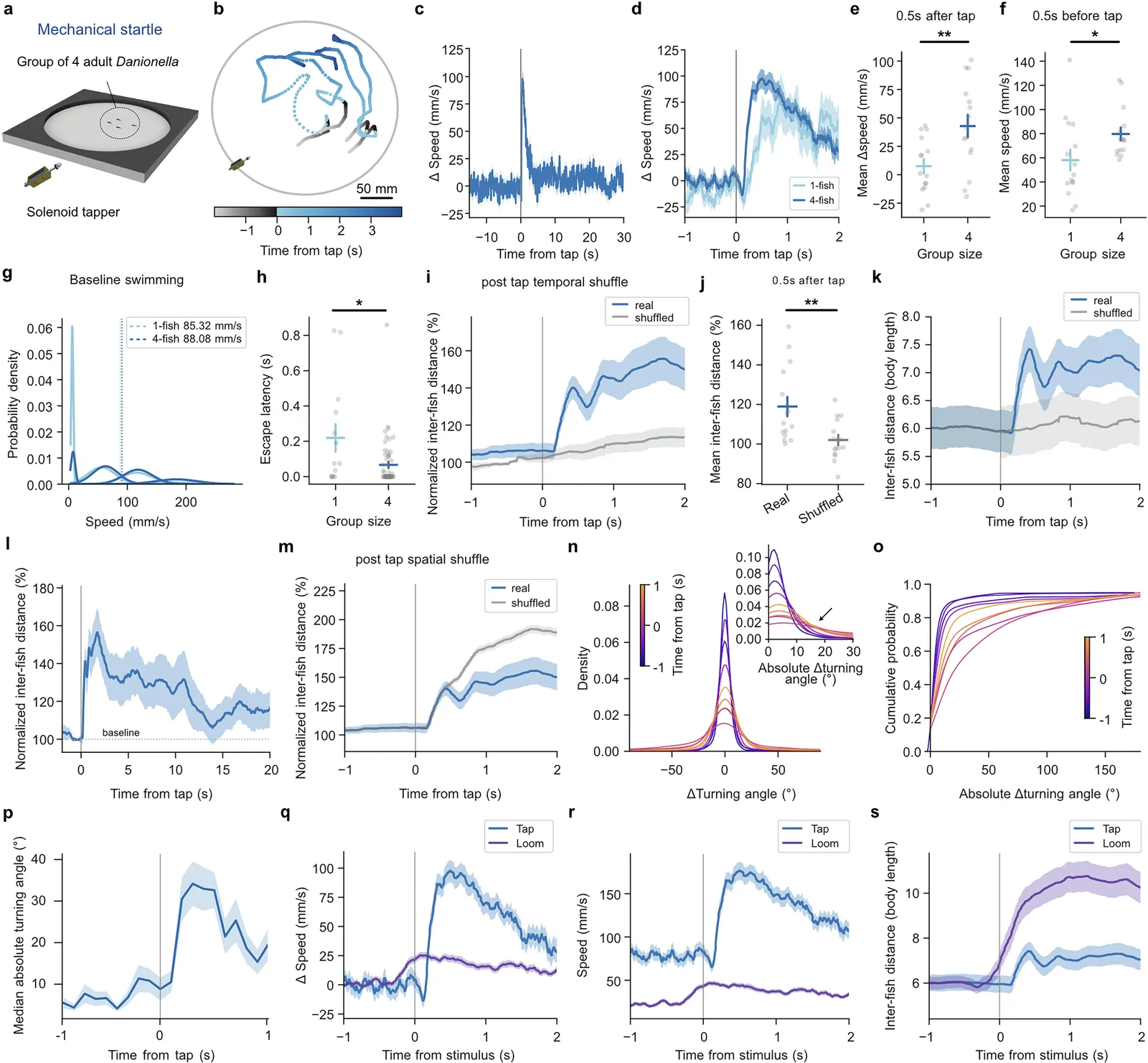
Mechanosensory startle behaviour of *D. cerebrum* groups. **a** Behavior paradigm for mechanosensation-evoked escape. A solenoid actuator is hidden underneath one of the corners of the circular arena. **b** Example fish trajectories of four fish over 6 s. Position of each fish is colored by time from tap. Note rapid change in position after the tap. **c** Change in speed at tap onset. n = 60 sessions from 20 fish. Note the punctate escape response in this longer time series. **d** Same as (**c**), but in the 3 s around tap onset. Speed of the single fish and the average speed of the four fish in a group were used for each trial. n = 15. **e** Change of speed in the 0.5 s from tap onset. n = 15 sessions. Each dot is a single fish randomly chosen in each trial. **f** Baseline speed in the 0.5 s before tap onset. n = 15 sessions. Each dot is a single fish or the average of all fish in each trial. **g** Probability density function (PDF) of baseline speed before tap onset. Vertical dashed lines mark the threshold for low vs high speed states each group size. **h** Escape latency. n = 14 and 58 sessions (fish reached high speed within 1 s). Each dot is a fish in a trial. **i-m** Inter-fish distance at tap onset for four-fish groups. (**i**) Normalized inter-fish distance. Gray line in is the distance when fish position is time-shuffled post tap. Normalized inter-fish distance at tap onset. Time-shuffled distance is present in gray. (**j**) Mean inter-fish distance in the 0.5 s after tap onset. Each dot is the average distance of all pairs in each trial. (**k**) Absolute inter-fish distance. Gray line is the distance when fish position is time-shuffled post-tap. (**l**) Normalized inter-fish distance over 20 s around tap onset. Note the recovery of inter-fish distance to baseline at ~10–15 s post stimulus. (**m**) Normalized inter-fish distance at tap onset. Gray line is the distance when fish position is identity-shuffled post tap. n = 15 sessions. **n**,**o** (**n**) Probability density function (PDF) and (**o**) Cumulative distribution function (CDF) of turning angle in 250 ms bins for fish in group of four. Inset in (**n**) shows the PDFs between 0° to 30°. Note the increase of large angles 250 ms before the tap onset marked with arrow. n = 60 sessions from 20 fish. **p** Median turning angle for all fish in groups of four at tap onset. n = 60 sessions from 20 fish. **q-s** Comparison of vision- vs. mechanosensation-evoked escape. (**q**) Change in speed at time of stimulus. (**r**) Speed at time of stimulus. (**s**) Inter-fish distance at time of stimulus. n = 15 and 20 sessions. *p < 0.05, **p < 0.01, ***p < 0.001. All data was from 10 single fish (1–5 sessions each) and 5 four-fish groups (1–5 sessions each), and shown in mean ± SEM. See Supplementary Table 1 for full statistics and sample sizes. Source data

Whereas some dense animal collectives show contractions or directional escape waves in response to external threats^6,20–22,36,38^, we observed that *D. cerebrum* groups scattered after exposure to a looming stimulus (Fig. 1h). Groups of four rapidly increased their mean inter-animal distances to 150.3 ± 12.9% of their baseline (Fig. 1i and Extended Data Fig. 1p–r) and returned to the group approximately 10 s afterwards (Extended Data Fig. 1q). These fish also scattered in response to a mechanical tap stimulus, with a short latency^39^ (Extended Data Fig. 2a–c and  Methods). Similar to visually evoked collective escape, groups of fish escaped more rapidly than individuals to taps (Extended Data Fig. 2d–h), increased their mean inter-animal distances to 118.9 ± 4.9% of their baseline (Extended Data Fig. 2i–l) and increased turning (Extended Data Fig. 2n–p).

Although collective escape to visual and mechanical stimuli are not identical (Extended Data Fig. 2q–s), we conclude that groups of *D. cerebrum* exhibit a collective escape behaviour by rapidly scattering. In response to either stimulus, groups only dispersed briefly (Extended Data Figs. 1q and 2l) and no more than a random arrangement (Extended Data Figs. 1r and 2m). This group scattering resembles flash expansions^38,40,41^, a type of collective escape strategy that may improve the fitness of group members by preventing predators from following all animals in the group at once^40,42,43^. However, we note that during escapes *D. cerebrum* are not actively avoiding each other; rather, they transiently depart from their typically coherent group structure.

## Vision of social partners enhances escape

**Extended Data Fig. 3:**
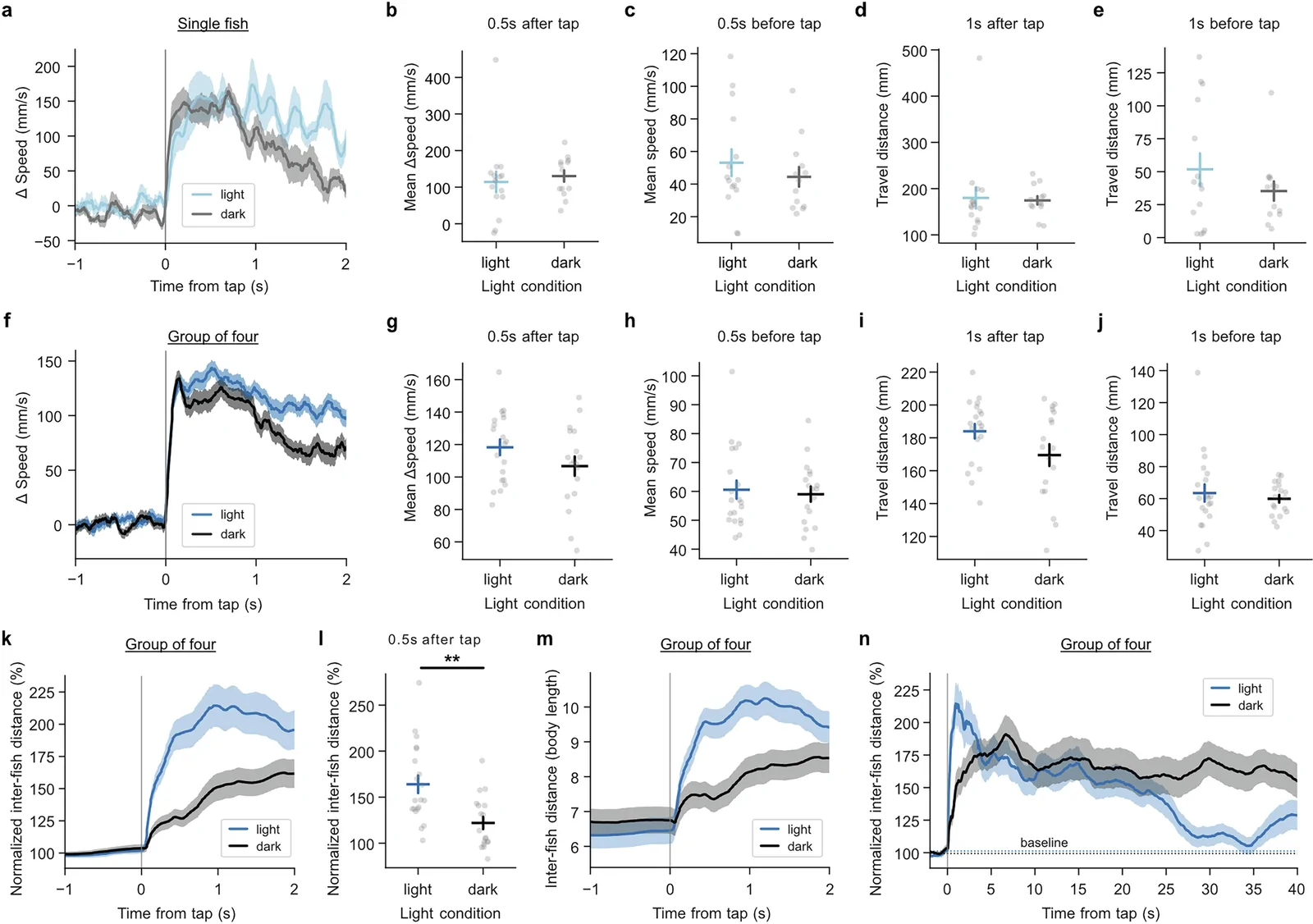
The role of vision in collective escape to mechanosensory startle. **a-e** Single fish escape from mechanosensory startle in light and darkness: (**a**) speed increase at tap onset, (**b**) mean change in speed increase in the 0.5 s from tap onset, (**c**) baseline speed in the 0.5 s before tap onset, (**d**) travel distance in the 1 s after tap onset, and (**e**) travel distance in the 1 s before tap onset. n = 15 and 13 sessions. Each dot is a trial from a single fish. **f-j** Fish in a group of four escape from mechanosensory startle in light and darkness: (**f**) speed increase around tap onset, (**g**) mean change in speed in the 0.5 s from tap onset, (**h**) baseline speed in the 0.5 s before tap onset, (**i**) travel distance in the 1 sec from tap onset, and (**j**) baseline travel distance in the 1 sec before tap onset. n = 20 and 18 sessions. Each dot is the average of all fish in each trial. **k** Normalized inter-fish distance around tap onset. n = 20 and 18 sessions. **l** Mean inter-fish distance in the 0.5 s after tap onset. n = 20 and 18 sessions. Each dot is the average of all fish pairs in each trial. **m** Inter-fish distance at tap onset. n = 20 and 18 sessions. **n** Normalized inter-fish distance at tap onset over 45 s. Note the inter-fish distance in dark condition didn’t recover at the end. *p < 0.05, **p < 0.01, ***p < 0.001. Data are shown in mean ± SEM. See Supplementary Table 1 for full statistics and sample sizes. Source data

*D. cerebrum* school using their vision of social partners^13,15,32^, and thus we hypothesized that individuals modify their escape behaviours by obtaining information from conspecifics using vision. We tested this hypothesis by quantifying and manipulating vision during escape behaviour. We first tested the mechanosensory escape of groups in light and darkness. Loss of visual cues did not influence the fast tap-evoked escape behaviour of single fish or groups, with comparable changes in short-latency speed and travel distance (Extended Data Fig. 3a–j). However, groups required vision to effectively scatter; darkness reduced tap-evoked scattering and prevented group reformation (Extended Data Fig. 3k–n). Therefore, vision of social partners facilitates collective scattering in response to a mechanical startle, consistent with prior findings of the importance of vision in the collective escape of other fish species^5,6,26^.

**Fig. 2:**
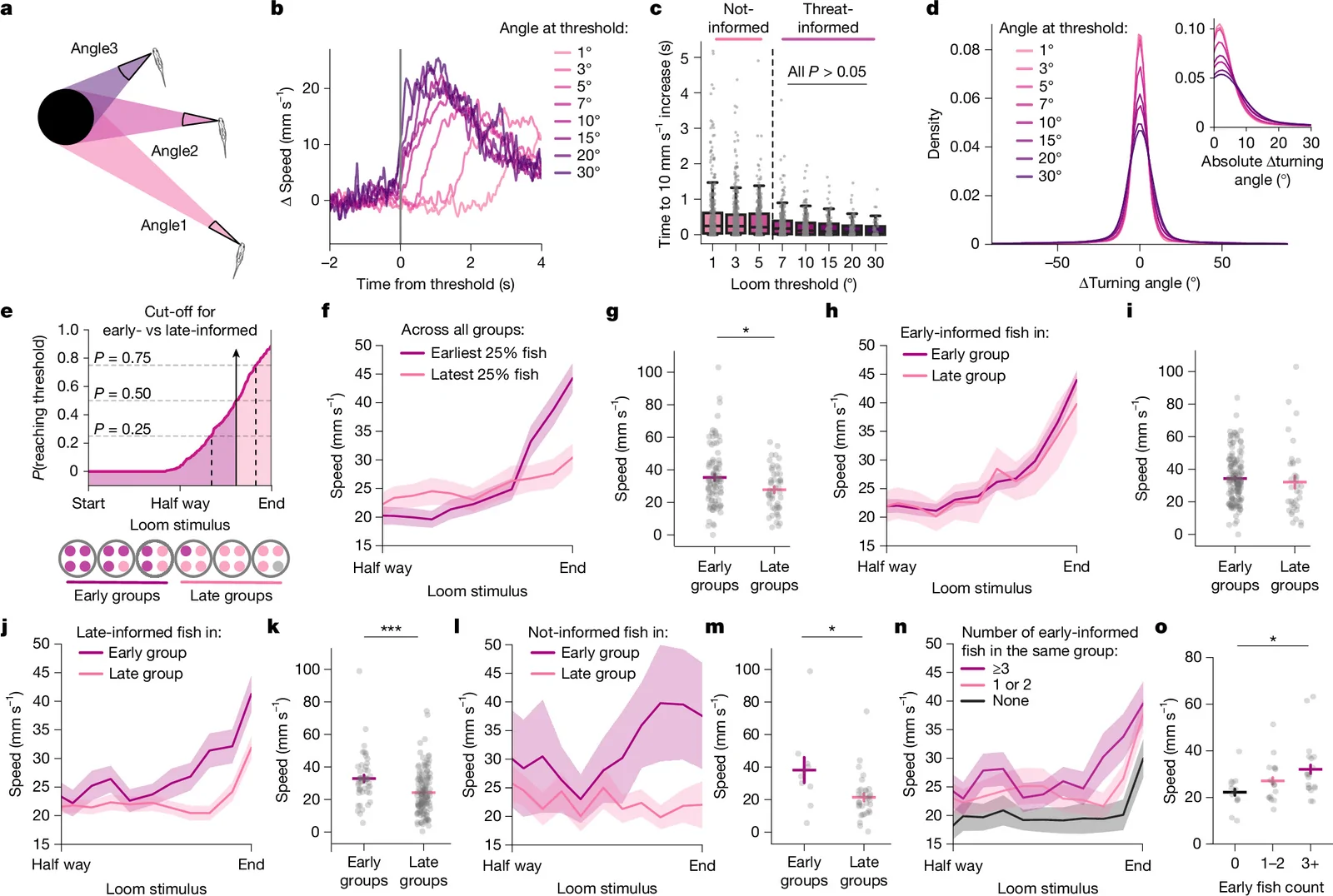
Visual access to escaping social partners drives escape in uninformed fish. **a**, Schematic of eye angle tracing of looming stimulus for multiple fish. Note that the same loom stimulus occupies unequal angular size in the visual field for each fish. Drawing adapted with permission from ref. ^12^, Elsevier. **b**, Change in speed after fish see defined loom thresholds (grey line). Over 62 sessions, *n* = 376 to 58 fish for threshold from 1° to 30°. **c**, Time for speed to increase by 10 mm s^−1^ after the grey line in **b**. Over 62 sessions, *n* = 372 to 58 fish for threshold from 1° to 30°. Box plot displays 25th, 50th and 75th percentiles, with whiskers extending to 1.5× the interquartile range or minimum and maximum values. **d**, Probability density function (PDF) of turning angle in 250 ms during which fish see defined loom thresholds. Inset shows PDFs between 0° to 30°. Over 62 sessions, *n* = 372 to 58 fish for threshold from 1° to 30°. **e**, Top, cumulative density function of time to 7° loom angle for each fish. Time at *P* = 0.5 is used to classify individual fish as early- or late-informed. Note the curve ends at 0.88, as fish that never see a 7° loom are classified as not-informed. *n* = 376 fish in 62 sessions. Bottom, classification of early or late groups, based on the composition of individual fish in the group (early, late and not-informed individuals are shown as purple, pink and grey dots, respectively). **f**,**g**, Speed during the final half (**f**) and in the last 20% (**g**) of the loom stimulus in the 25% earliest- and latest-informed fish. Each dot in **g** represents a fish from a single trial. *n* = 94 (earliest 25%) and 51 (latest 25%) fish in 54 sessions. **h**–**o**, Speed during the final half (**h**,**j**,**l**,**n**) and in the last 20% (**i**,**k**,**m**,**o**) of the loom stimulus. Each dot is a fish from a single trial. **h**,**i**, Early-informed fish in early- or late-informed groups. *n* = 153 (early-informed) and 35 (late-informed) fish in 49 sessions. **j**,**k**, Late-informed fish in early- or late-informed groups. *n* = 49 (early) and 139 (late) fish in 49 sessions. **l**,**m**, Not-informed fish in early- or late-informed groups. *n* = 10 (early) and 33 (late) fish in 23 sessions. **n**,**o**, Late-informed fish with 0, 1–2 or ≥3 early-informed neighbours. Each dot in **o** is the average of all late-informed fish in the trial. *n* = 13 (0 early fish), 17 (1–2 early fish) and 19 (3+ early fish) sessions. All data were from 13 groups (4–8 fish per group, 4–5 sessions each), and are shown as mean ± s.e.m. See Supplementary Table 1 for full statistics and sample sizes. *P < 0.05, ***P < 0.001. Source data

**Extended Data Fig. 4:**
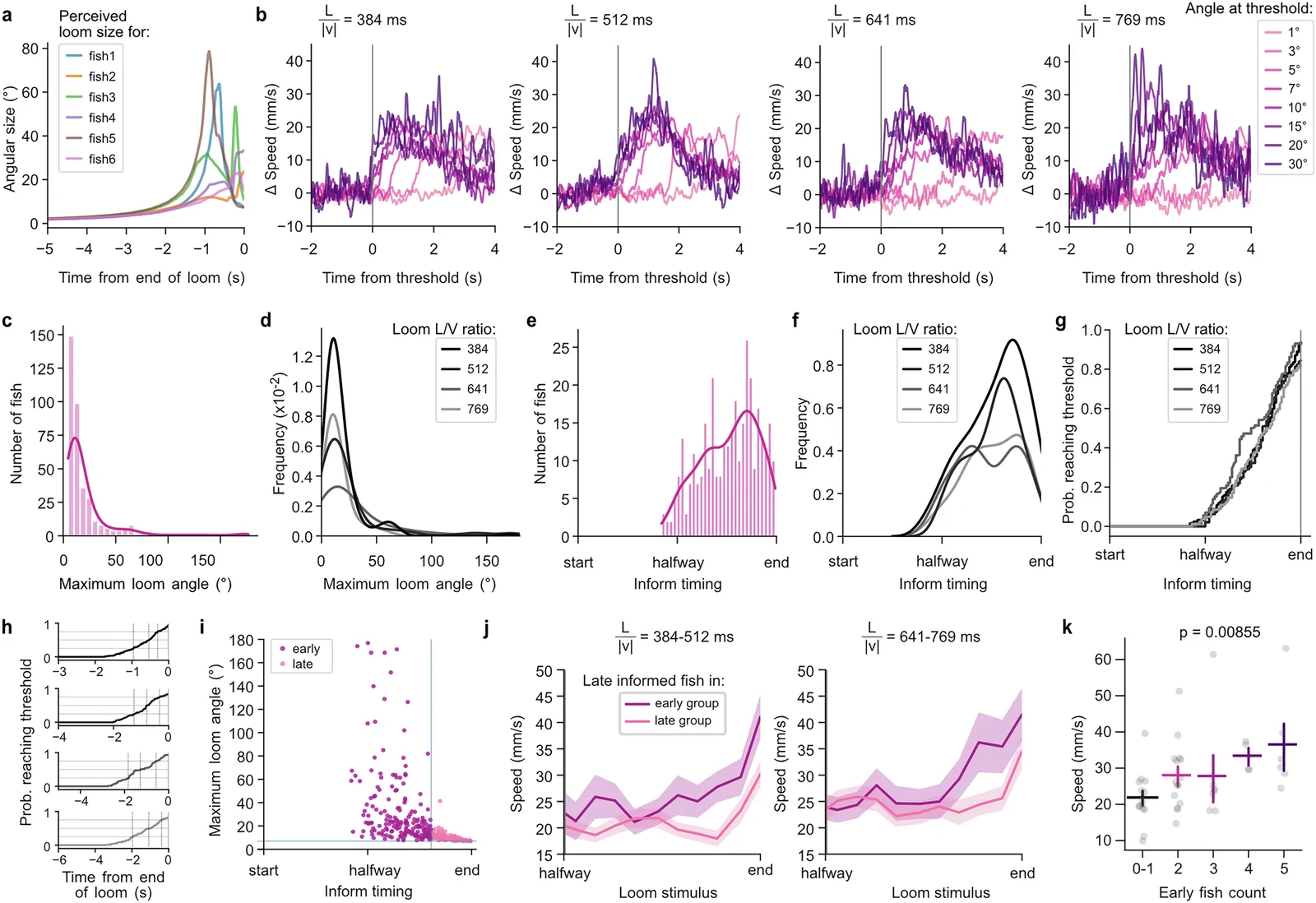
Visual basis of collective escape movements to looming stimuli. **a** Example eye angle traces of loom stimulus for fish in a group over 5 s. Note that threat proximity varies across fish in the group. **b** Change in speed after fish see specific loom thresholds (gray line). From left to right: sessions with loom approaching at length/velocity (L/V) ratio from 384 to 769 ms. **c** Distribution of maximum loom visual angle seen by individual fish. n = 376 sessions. **d** Probability density function of maximum loom visual angle seen by individual fish separated by approaching speed of loom durations. n = 126, 96, 72, and 82 sessions for loom L/V of 384, 512, 641, and 769 ms. **e** Distribution of time for loom stimulus reaching critical angle for each individual fish (ratio of trial length). n = 376 sessions. **f** Probability density function of time for loom stimulus reaching critical angle for each individual fish separated by approaching speed of loom durations. n = 118, 81, 67, and 67 fish for loom L/V of 384, 512, 641, and 769 ms. **g** Cumulative density function of reaching 7° visual loom angle over time across fish separated by approaching speed of loom durations. Note the overlap between probability curves. N = 126, 96, 72, and 82 fish for loom L/V of 384, 512, 641, and 769 ms. **h** Cumulative density function of fish seeing 7° visual loom angle over time. n = 126, 96, 72, and 82 sessions for loom L/V of 384, 512, 641, and 769 ms. **i** Scatterplot of maximum loom angle and time of reaching critical angles for all sessions. Vertical lines show the critical angle (7°) and inform timing threshold (P = 0.5). n = 376 sessions. **j** Speed during the last half of the loom stimulus of the “late informed” fish in early or late informed groups. Left: sessions during slow approaching loom (L/V = 384 or 512 ms) n = 28 and 85 sessions in early and late informed groups. Right: sessions during fast approaching loom (L/V = 641 or 769 ms) n = 21 and 54 sessions in early and late informed groups. **k** Speed of the last 20% of loom stimulus. n = 15, 15, 7, 4, and 6 sessions for groups with 0–1, 2, 3, 4, and 5 early informed individuals. Each dot is the average of all late informed fish in each session. *p < 0.05, **p < 0.01, ***p < 0.001. All data was from 13 groups (4–8 fish per group, 4–5 sessions each), and shown in mean ± SEM. See Supplementary Table 1 for full statistics and sample sizes. Source data

Although the mechanical threat stimulus is delivered synchronously to all group members, the visual loom stimulus is perceived asynchronously by individual fish, depending on the proximity of each fish to the approaching visual object; the size of the looming sphere in each individual’s field of view depends on their position in the arena relative to its approach (Fig. 2a and Extended Data Fig. 4a). We measured swimming speed after the loom passed different angular thresholds in the field of view for each fish, ranging from 1 to 30° (Methods). We observed that speed immediately increases upon passing a threshold of 7°, whereas smaller angles evoke delayed escape behaviour (Fig. 2b,c and Extended Data Fig. 4b). The time from passing the angular threshold to increasing speed is not significantly different amongst trials with an angular size threshold of 7° or higher (Fig. 2c). This critical angle for loom-evoked escape is similar to those reported for adult zebrafish^44^, but smaller than those reported for larval zebrafish^45^. We also observed a shift to increased turning amplitude at these larger visual angles (Fig. 2d).

Within groups of four or more *D. cerebrum*, we examined the angular and temporal distribution of the individuals’ loom angles across the duration of the loom stimulus (Extended Data Fig. 4c–f). We calculated the cumulative probability of the loom stimulus reaching the 7° critical angle across individual fish and observed a similar temporal pattern (Extended Data Fig. 4g,h), obtaining a cut-off time (80.5% through the loom) for all trials when half of fish see the loom at critical angle size or larger (Fig. 2e). We then classified individual fish as ‘early-informed’ or ‘late-informed’ depending on whether that individual’s time to critical loom angle was before or after the cut-off time, respectively (Fig. 2e and Extended Data Fig. 4i). Across stimulus durations, early-informed fish increased their speed more rapidly than late-informed fish as the looming stimulus approached (Fig. 2f,g).

To determine whether individuals benefitted from having early-informed social partners, we categorized groups as predominantly ‘early’ or predominantly ‘late’ (Fig. 2e and  Methods). Early-informed fish comprised 72.1% (153 out of 212 fish) of the individuals in early groups and 16.9% (35 out of 207 fish) of those in late groups. We found that early-informed individuals moved at similar speeds regardless of the status of their group (Fig. 2h,i), suggesting that their direct observation of the looming stimulus drives their escape behaviour. By contrast, late-informed individuals increased their speed earlier when they were members of early groups, in contrast to those in late groups (Fig. 2j,k and Extended Data Fig. 4j). Additionally, fish characterized as ‘not-informed’—those that never reach the 7° loom threshold—only increased their speed if they were members of an early-informed group (Fig. 2l,m). Furthermore, the speed of late- or not-informed observers increases with greater number of early-informed fish in their group (Fig. 2n,o and Extended Data Fig. 4k). These results indicate that individuals with limited direct perception of a looming threat stimulus can nonetheless execute a timely escape when their social partners are informed of the threat.

## Response to virtual conspecific escape

**Fig. 3:**
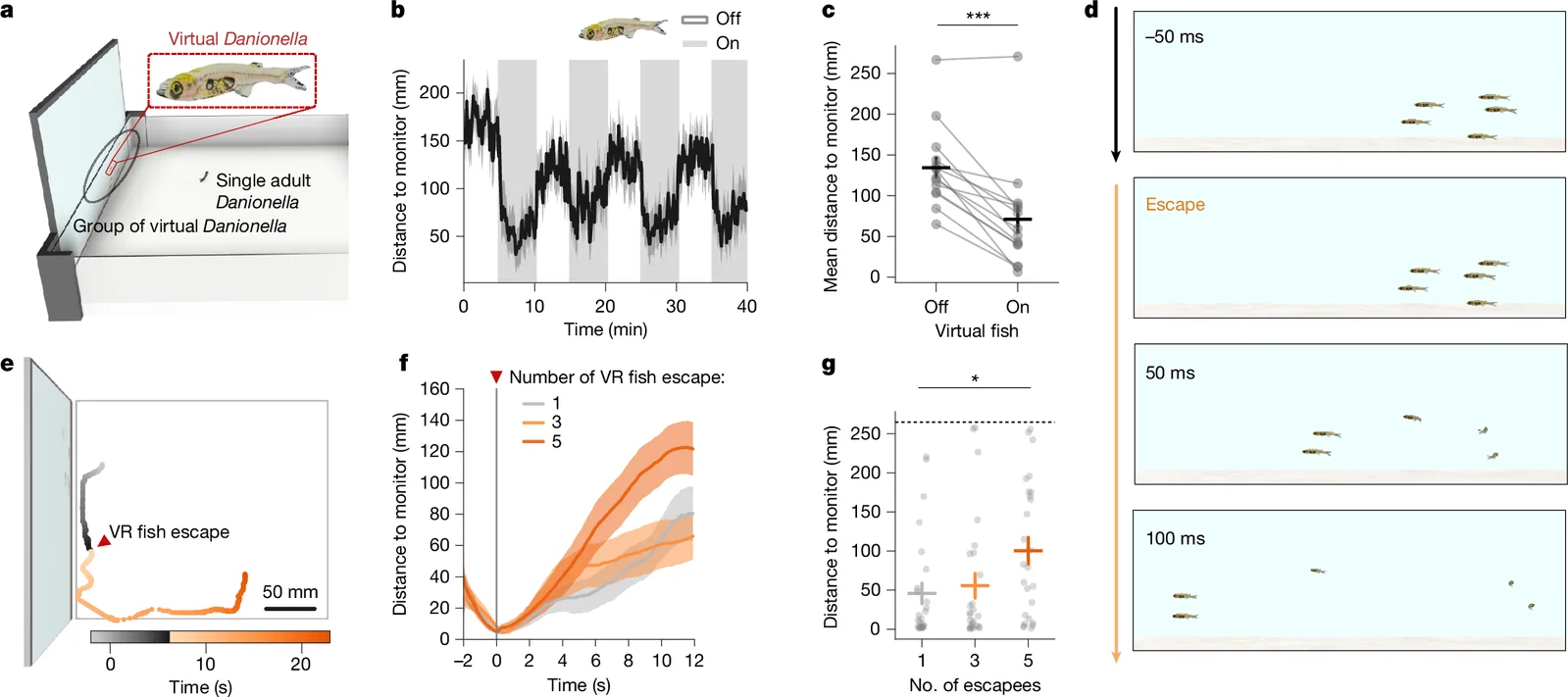
*D. cerebrum* withdraw from virtual fish that escape. **a**, Behaviour paradigm for virtual fish-evoked behaviour. A school of visually realistic *D. cerebrum* is displayed on the LCD monitor on the side of the square arena in which a single *D. cerebrum* freely swims. **b**, Distance from the monitor over 40 min. The virtual school was rendered for 4× 5-min segments, indicated by grey shading. *n* = 14 fish. **c**, Mean distance from the monitor during display of virtual conspecifics. *n* = 14 fish. Each dot is the mean distance of a fish during the 20 min with or without virtual conspecifics displayed. **d**, Example frames of virtual *D. cerebrum* group on the monitor in a span of 150 ms at a proximity-triggered escape. Note the scattering of the virtual school away from the screen during the escape. Images of virtual fish are pixelated, as they appear when displayed to real fish in experiments. **e**, Example fish trajectory over 30 s. Positions are coloured by the time to onset of virtual conspecific escape. Note that the fish swim in parallel to the monitor (grey dots) before the escape event is triggered. VR, virtual reality. **f**,**g**, Distance from monitor. **f**, Virtual escape onset is marked with a grey line at 0 s. **g**, Mean distance is taken from 5 to 12 s after escape onset. The dashed line is the maximal distance (265 mm). *n* = 26 fish for sessions of 1, 3 and 5 fish escaping in a virtual school of 5 fish. Data are mean ± s.e.m. See Supplementary Table 1 for full statistics and sample sizes. *P < 0.05, ***P < 0.001. Fish image in **a**,**b**,**d** adapted from ref. ^59^, CC BY 4.0. Source data

**Extended Data Fig. 5:**
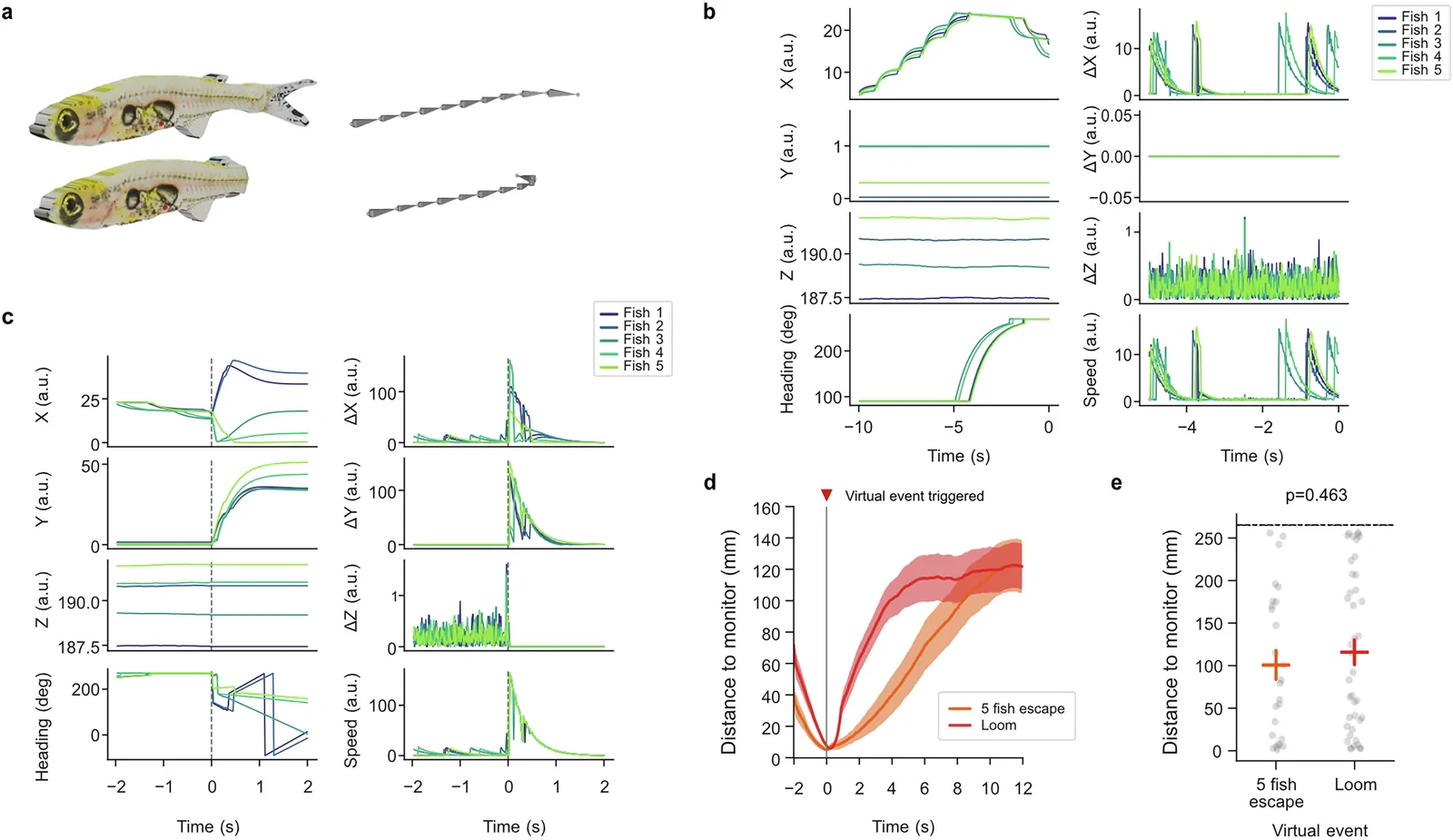
Movements of virtual *D. cerebrum*, and behavioural responses. **a** *D. cerebrum* model texture (left) and skeleton (right) used in the virtual reality environment. Note the bending of the tail in the model follows the curvature of the skeleton in three- dimensional space. Fish image adapted from ref. ^59^, CC BY 4.0. **b** Example kinematics of the virtual *D. cerebrum* in the VR environment over 10 s of normal swimming. Left: (top to bottom) X-, Y-, Z-positions and heading direction. Right: (top to bottom) derivatives of X, Y, Z, and speed. **c** Example kinematics of the virtual *D. cerebrum* in the VR environment over 4 s at the onset of virtual escape (dashed line). Left: (top to bottom) X-, Y-, Z-positions and heading direction. Right: (top to bottom) derivatives of X, Y, Z, and speed. **d**,**e** Distance from monitor. (**d**) Virtual escape onset or loom onset is marked with a gray line at 0 s. (**e**) Mean distance is taken from 5 to 12 s after escape onset. Dotted grey line is the maximal distance (265 mm). n = 26 and 41 sessions for virtual fish escape and loom. Note the five-virtual-fish escape data are replotted from Fig. 3f. *p < 0.05, **p < 0.01, ***p < 0.001. Data are shown in mean ± SEM. See Supplementary Table 1 for full statistics and sample sizes. Source data

Our results suggest that fish can escape after receiving indirect information about a threat from the visual perception of their social partners’ escape behaviours. Testing this requires experimental conditions in which single *D. cerebrum* can observe conspecifics escaping but cannot directly sense a threat. To present fish with experimentally controllable social partners, we developed a visual virtual reality environment populated by three-dimensional fish models whose appearance and movement closely match those of adult *D. cerebrum* (Fig. 3a, Extended Data Fig. 5a,b and  Methods). We found that individual adult *D. cerebrum* readily engage with these virtual partners, swimming at an average distance of 71.1 ± 13.3 mm from the monitor when they are displayed, compared to 134.4 ± 17.7 mm at baseline (Fig. 3b,c).

We developed a closed-loop paradigm in which virtual fish would execute an escape action when real *D. cerebrum* came within 16 mm of the virtual groups (>60 s inter-stimulus interval; Fig. 3d and Extended Data Fig. 5c). Because *D. cerebrum* scatter during collective escape (see Fig. 1h and Extended Data Fig. 2i), we reasoned that real fish would often escape away from the screen, rather than into it. We observed that individual fish executed avoidance behaviours to these escaping virtual fish (Fig. 3e), which scaled with increasing numbers of escaping virtual partners (Fig. 3f,g). This escape behaviour was comparable in magnitude, but delayed in onset, to escape from direct perception of a threat; when we replaced the conspecific escape action with a visual looming stimulus, observers escaped at a faster rate (Extended Data Fig. 5d,e and Methods). This finding is in agreement with prior work showing that socially transmitted information decays with distance^6,46,47^, a strategy to prevent continuous unabated transmission of threat information^22,46^.

Therefore, we conclude that collective escape in *D. cerebrum* is mediated by the visual perception of the actions of one’s social partners: observing an escaping conspecific promotes one’s own escape. For individual fish to achieve this, their visual systems must be capable of detecting these escape actions.

## Neural encoding of biological motion

Neurons in the visual systems of *D. cerebrum* and zebrafish are responsive to stimuli whose size, shape and motion patterns resemble conspecifics^15,18^. These objects robustly activate populations of neurons in the optic tectum (homologue of superior colliculus in mammals), a retino-recipient midbrain structure that mediates innate visually driven orienting behaviours in vertebrates ranging from fish to birds to primates^16,17,36,48^. To examine how neural populations in the *D. cerebrum* visual system respond to the escape behaviours of conspecifics, we recorded neural activity while fish observed virtual social motion stimuli with routine swimming movements versus those with sudden escape behaviour (Methods).

**Fig. 4:**
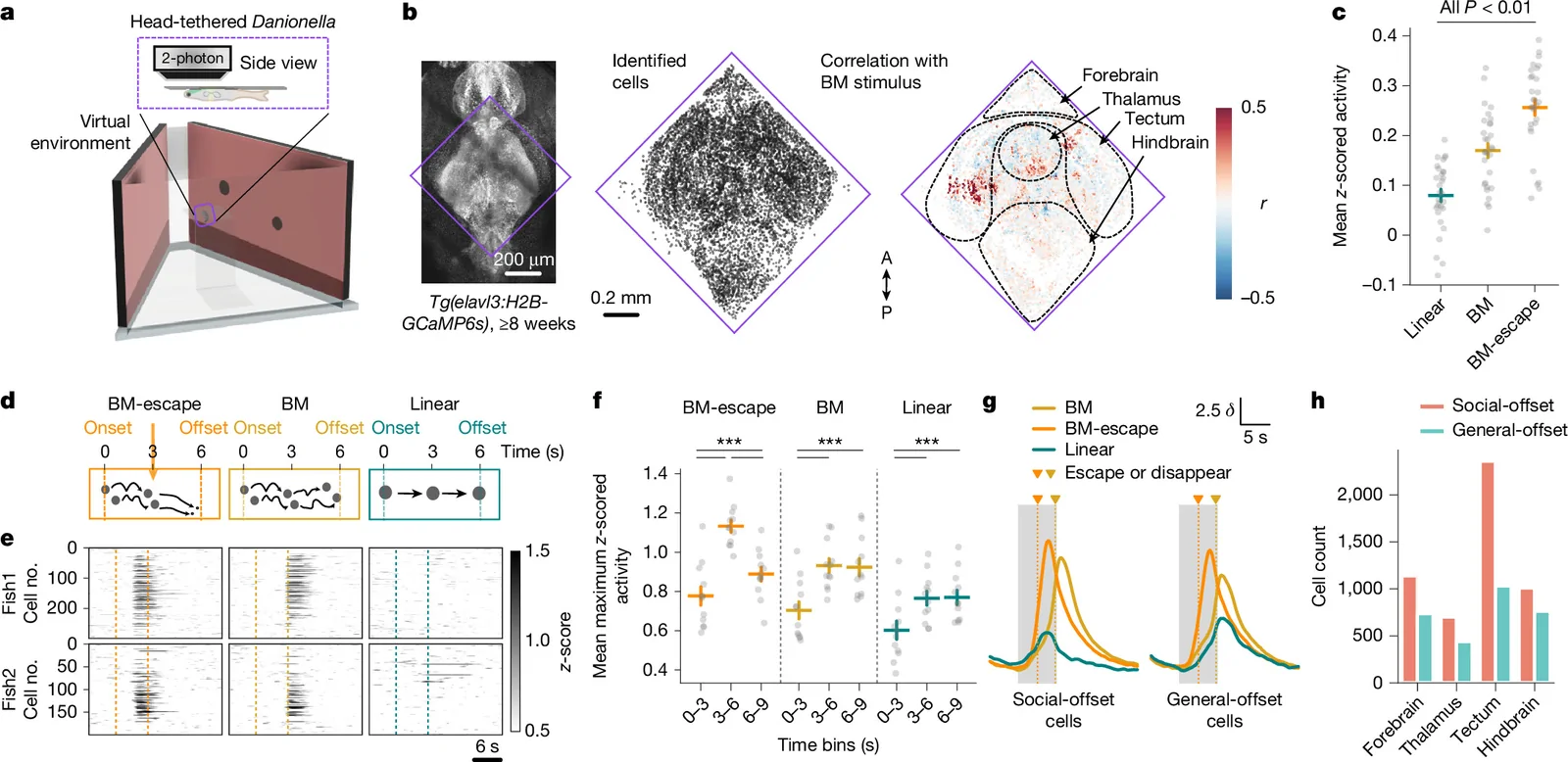
Neurons respond to the actions of visual stimuli with biological motion. **a**, Schematic of in vivo two-photon calcium imaging in head-tethered *D. cerebrum* with panoramic visual display of virtual environment. Inset, side view of fish suspended under a coverslip. **b**, Left, dorsal view of adult *D. cerebrum* brain expressing nuclear-localized GCaMP6s. Field of view (purple box) covers the midbrain optic tectum and surrounding brain regions. Middle, positions of all cells extracted in an example session. Right, cell positions coloured by the correlation coefficient of their activities to the biological motion (BM) stimulus. A, anterior; P, posterior. **c**, Maximal *z*-scored fluorescence of cells responsive to each stimulus type. Each dot represents the average of all cells in one session. *n* = 30 sessions from 12 fish. **d**, Schematics of stimulus movements for the BM-escape, BM and linear motion during 6 s of stimulus presentation. **e**, Trial-averaged responses of neurons in two example fish, to each stimulus. Note the timing differences in responses to BM and BM-escape stimuli, and the lack of response to the linear stimulus. *n* = 300 and 250 cells. **f**, Mean of the maximum *z*-scored activity of socially responsive cells, grouped by their time-to-peak for each stimulus. *n* = 12 fish. Each dot represents the average of all cells recorded per fish. **g**, Mean *z*-scored responses to each stimulus type from social-offset cells (left) and general-offset cells (right). *n* = 5,237 (social-offset) and 2,970 (general-offset) cells. Grey shading shows the stimulus presentation time. **h**, Total cell number across anatomically defined regions in 12 recorded fish. *n* = 1,137 (forebrain), 702 (thalamus), 2,357 (tectum) and 1,012 (hindbrain) social-offset cells, and *n* = 736 (forebrain), 439 (thalamus), 1,032 (tectum) and 763 (hindbrain) general-offset cells. Data are mean ± s.e.m. See Supplementary Table 1 for full statistics and sample sizes. ***P < 0.001. Source data

**Extended Data Fig. 6:**
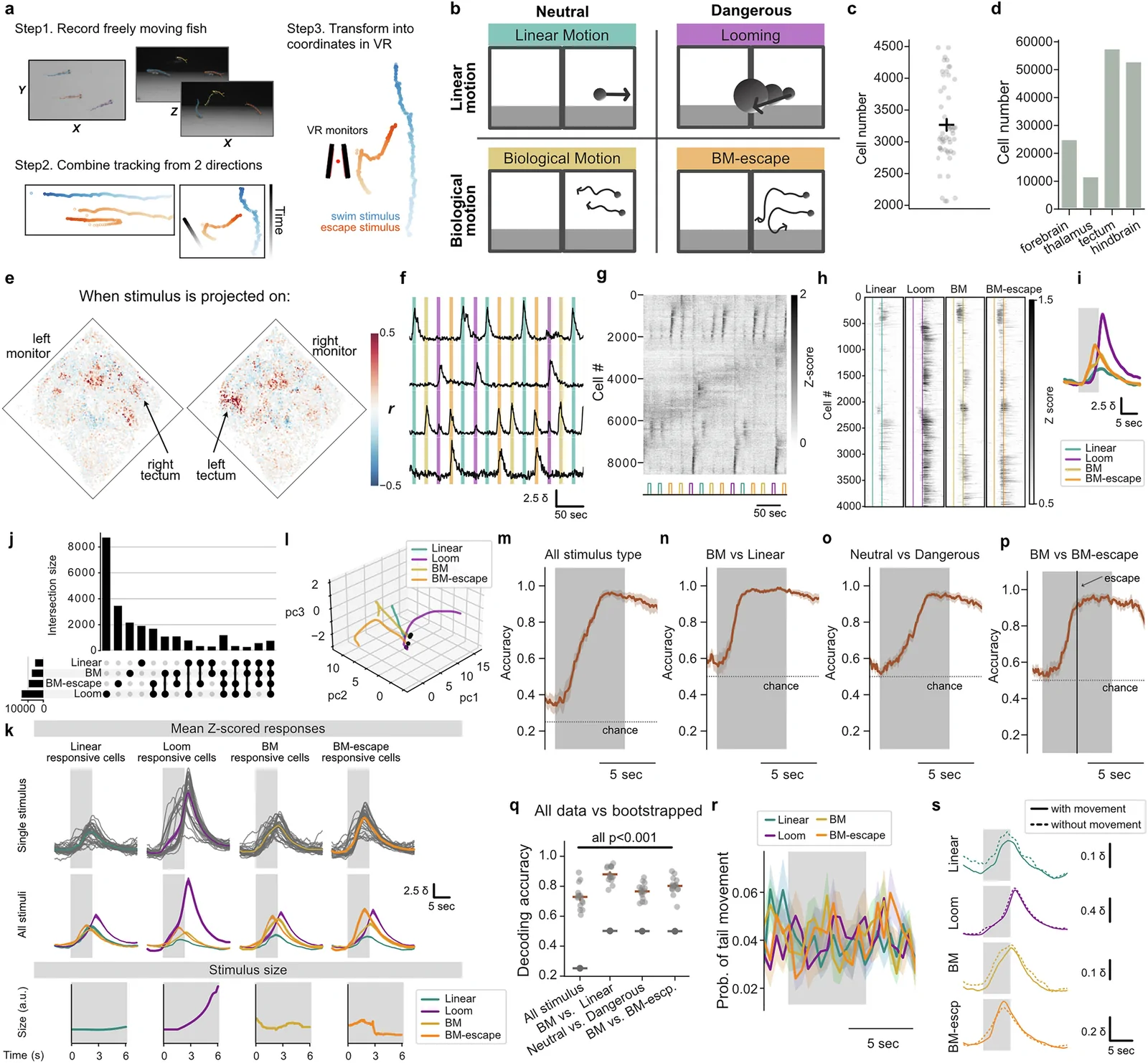
Neural responses to visual stimuli. **a** Workflow of making dot stimuli moving with naturalistic motion, from freely moving *D. cerebrum* recordings. **b** Illustration of the four types of visual stimuli where simple spheres are programmed to move with: linear motion (smooth lateral translation), looming (smooth approach to observer), biological motion (BM; burst-and-glide swimming actions extracted from recordings of real *D. cerebrum*), and escape after biological motion (BM-escape; actions extracted from recordings of real *D. cerebrum*, where the recorded fish escaped). **c** Mean cell number recorded per imaging plane. n = 50 planes from 30 sessions in 12 fish. (3267 ± 92). **d** Total cell number recorded across anatomical regions. n = 25101, 11823, 57649, 53040 cells in forebrain, thalamus, tectum, and hindbrain, respectively. **e** Positions of all cells extracted in two example sessions, and colored by the correlation coefficient of their activity to the swimming-dot stimulus. Swimming stimulus was displayed on opposite monitors between these two sessions. Note the lateralization of highly correlated neuronal clusters on the contralateral side of the visual stimulus. **f-h** Example neural responses with color blocks and lines showing the stimulus presentation time and type in (**b**). (**f**) Short epoch of stimulus driven single neurons’ responses in an example fish over 300 sec. Example raster map of all recorded cells (**g**) and trial averaged responses from all responsive cells (**h**) in an example fish. n = 8579 and 4006 cells. **i** Mean z-scored response to each stimulus type from all responsive cells in an example fish overlaid in (**h**) with gray shade marking stimulus presentation time. n = 4006 cells. **j** Intersection plot (UpSet) showing response combinations of neurons in all fish recorded. n = 23452 cells. **k** Mean z-scored response to different stimulus types in all fish recorded. Gray shade marks the time of stimulus presentation. Top: gray lines are the mean activity of all cells recorded in one session, and colored line is the average of the gray lines. Middle: thick lines show the mean response of cells responsive to each stimulus; thin lines show the response of the same cells to other stimulus types. n = 13827, 30556, 17871, and 20590 responsive cells to linear, loom, BM, and BM-escape stimulus from 30 sessions in 12 fish. Bottom: Visual occupancy of different stimulus types over time. **l** Example low-dimensional trajectory of trial-averaged stimulus responses, beginning at 1 s before the stimulus starts (black dot) and progressing for 6 s until the stimulus ends. Trajectories are colored by stimulus type and are shown for the first three principal components (PCs). **m-p** Accuracy of support vector machine (SVM) models over time. Gray shade marks the time of stimulus presentation. Chance level is calculated from mean accuracy score of bootstrapped models. N = 15 sessions (15 iterations of bootstrapped each). Models are trained to classify (**m**) all four stimulus types, **n** biological motion vs. linear motion, (**o**) Same as m, but models are trained to classify neutral vs. dangerous stimulus, and (**p**) BM vs. BM with escape action. **q** Performance of SVM models. Each point represents the mean accuracy of a model trained with cells recorded in one session, taken over 6 s during stimulus presentation. N = 15 (15 iterations of bootstrapped each). Gray bars are the average performance of bootstrapped models. **r** Probability of tail movement of the head-tethered fish during stimulus presentation (see Methods). Gray shade marks the time of stimulus presentation. **s** Mean z-scored response to different stimulus types in all fish recorded, separated by the presence of tail movement. Mean probability of tail movements during stimulus presentation is used to classify sessions with motion (n = 2704 trials, in solid lines) or without motion (n = 832 trials, in dashed lines). *p < 0.05, **p < 0.01, ***p < 0.001. All data are from 12 fish (1–4 imaging sessions, 1–2 plane per session). Each session included 4–10 trials per stimulus type. Data are shown in mean ± SEM. See Supplementary Table 1 for full statistics and sample sizes. Source data

Using a *D. cerebrum* transgenic line expressing a nuclear-localized calcium indicator in almost all neurons, we performed large-scale two-photon calcium imaging from fish tethered within a panoramic visual display environment^15^ (Fig. 4a). We displayed simplified biological motion stimuli^15,18,49^: fish-sized spherical objects whose motion in virtual space is derived from recordings of real *D. cerebrum* groups moving with their characteristic burst-and-glide kinematics (Extended Data Fig. 6a). Tethered *D. cerebrum* observed objects that either moved in a typical swimming pattern (biological motion group), moved normally then escaped (biological motion-escape group), or moved with smooth non-biological motion (linear motion group), in addition to a looming stimulus (Extended Data Fig. 6b and Methods). We presented each stimulus for 6 s, in a pseudorandom order with a 15 ± 1 s inter-stimulus interval, and recorded neural activity from 12 adult *D. cerebrum*, observing each stimulus 7–10 times.

We recorded from 3,267 ± 92 neurons in a 978 μm × 978 μm field of view in each of the 50 planes in a total of 12 fish (1–2 planes per imaging session, 1–4 sessions per fish; Extended Data Fig. 6c). After motion correction and cell extraction (Methods), we sorted neurons by their anatomical position to identify cells in the midbrain optic tectum, as well as surrounding diencephalic, forebrain, and hindbrain regions (Fig. 4b and Extended Data Fig. 6d). We identified neurons responsive to visual stimuli across all recorded brain areas (Fig. 4b and  Methods), including cells in portions of the optic tectum corresponding to the retinotopic position of stimuli presented to the contralateral eye (Extended Data Fig. 6e). We also observed visually driven cells in the dorsal thalamus and telencephalon, regions identified as responsive to biological motion in juvenile zebrafish^18,50^. We classified neurons by their responses to visual stimuli, identifying cells with significant responses to individual or combinations of stimuli (Extended Data Fig. 6f–i; *n* = 23,452 neurons in 12 fish, *P* < 0.05). Many cells were activated by looming stimuli (Extended Data Fig. 6h–k), which are innately salient^36^ and expand to occupy most of the visual space. Although many neurons responded to multiple stimuli (Extended Data Fig. 6j), a decoder trained on neural activity was able to distinguish amongst stimulus types (Extended Data Fig. 6l–q and Methods).

We focused our analysis on the smaller objects presented to similar positions in visual space: the two biological motion stimuli (routine swimming and escape) as well as the non-biological motion stimulus. Of these stimuli, the magnitudes of neural responses to biological motion stimuli were greater than those of responses to the linear motion object (*P* = 0.0021 and *P* = 2.43 × 10^−^^10^, respectively), and responses to the biological motion stimulus that escaped were greater than those to swimming motion (*P* = 0.0056; Fig. 4c). These responses were unaffected by the fish’s swimming movements during imaging (Extended Data Fig. 6r,s).

Thus, stimuli of social relevance to the observer increase the visual response of cells in the midbrain and thalamus. Similar types of neuronal gain enhancement have been identified in the tectum or colliculus of other species in response to innately salient visual stimuli^16,17^. We hypothesize that conspecific escape and social actions are particularly salient to *D. cerebrum*, which therefore drives an increased response in visual neurons.

## Selectivity to escape-like movements

**Extended Data Fig. 7:**
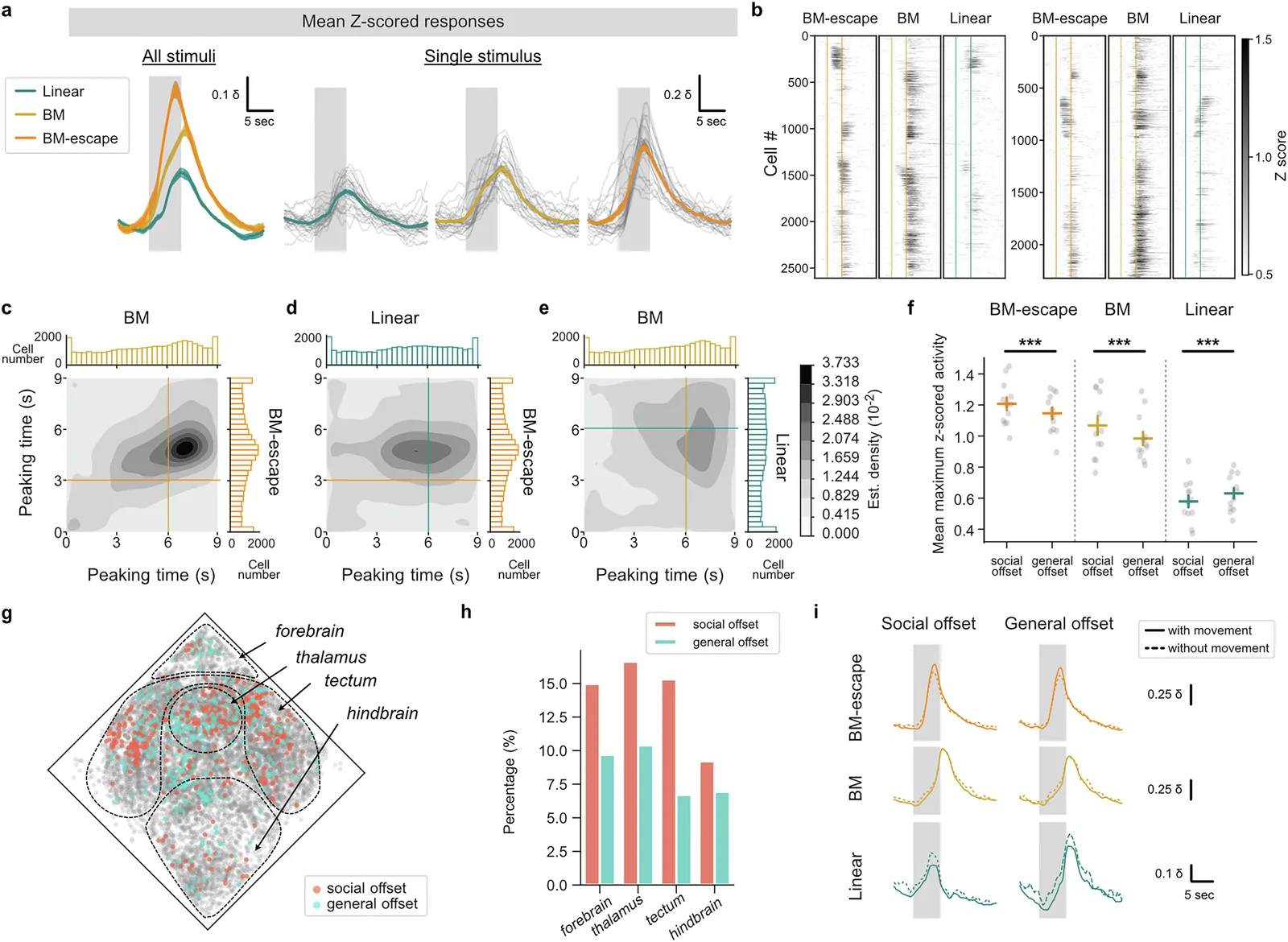
Further characterizations of visual responses to moving objects. **a** Left: mean z-scored response to different stimulus types in all fish recorded. Right: average response across sessions (colored lines) and mean activity of all cells recorded in each session (gray lines). n = 40375 cells. **b** Trial-averaged z-scored response to each stimulus type from all responsive cells in two example fish. Colored lines show the onset and offset of stimuli. n = 2610 and 2322 cells in the two fish. **c-e** Density maps (smoothed scatterplot) of peaking time in all visually responsive cells towards (**c**) BM and BM-escape, (**d**) linear and BM-escape, and (**e**) BM and linear stimulus. Time of escape and offset of BM or linear stimulus are indicated with colored lines. n = 40375 cells. **f** Mean maximum z-scored activity of “social offset” and “general offset” cells to each stimulus type. Each point is the average of all cells recorded in one fish. n = 5237 and 2970 cells. **g** Positions of all cells extracted in an example fish with “social offset” and “general offset” cells colored in coral and teal. Note the different spatial distribution of the two cell types in optic tectum and thalamus. **h** Percentage of visually responsive cells in each anatomical region (14.9%, 16.6%, 15.3%, 9.2% “social offset” cells and 9.6%, 10.3%, 6.7%, and 6.9% “general offset” cells in forebrain, thalamus, tectum, and hindbrain). **i** Mean z-scored response to different stimulus types in all fish recorded, separated by the presence of tail movement. Mean probability of tail movements during stimulus presentation is used to classify sessions with motion. n = 5237 and 2970 “social offset” and “general offset” cells. *p < 0.05, **p < 0.01, ***p < 0.001. All data are from 30 sessions in 12 fish (1–4 imaging session, 1-2 planes per session), and shown in mean ± SEM. See Supplementary Table 1 for full statistics and sample sizes. Source data

To determine how specific social actions are encoded in neural populations, we compared the timing of neural responses to objects with routine biological motion versus those with sudden escapes. We found a subpopulation of neurons whose activity increased upon the escape action of objects with biological motion, at the midway point of the stimulus (Fig. 4d,e and Extended Data Fig. 7a,b). These same neurons also increased their activity at the offset of the routine swimming stimulus, when the objects vanish at the end of the trial (Fig. 4d,e and Extended Data Fig. 7b). This stimulus-offset response was specific to the stimuli that move with biological motion; these cells were not driven by the offset of the linearly translating object (Fig. 4d,e and Extended Data Fig. 7b). Across all recording sessions, neurons preferentially responded to moments of stimulus escape and offset (Fig. 4f). Cells whose activity peaked after the escape action also peaked after offset of the biological motion stimulus (Extended Data Fig. 7c). Although we also observed a subset of cells that peaked in response to the offset of the linear motion object (Extended Data Fig. 7b), these responses were not clearly associated with the actions of objects with biological motion (Extended Data Fig. 7d,e).

We classified neurons as those that responded specifically to the escape or disappearance of biological motion objects (social-offset), versus those that responded to the offset of any stimulus (general-offset) (Fig. 4g and Extended Data Fig. 7f). We examined the spatial location of social-offset neurons (Extended Data Fig. 7g, *n* = 12 fish) and found 45.2% (2,357 out of 5,208 total) of these cells in the tectum and 13.4% (702 out of 5,208 total) in the dorsal thalamus (Fig. 4h and Extended Data Fig. 7h). Neither response type was modulated by the observer’s swimming movements (Extended Data Fig. 7i).

These data suggest that a subset of visual neurons that track objects with biological motion, such as social partners, are responsive to the disappearance of those objects. As adult *D. cerebrum* are capable of high-speed movements (Fig. 1 and ref. ^39^), a social partners’ sudden disappearance from their position in the visual field can be an indication of their rapid escape.

## Neural detection of conspecific actions

**Fig. 5:**
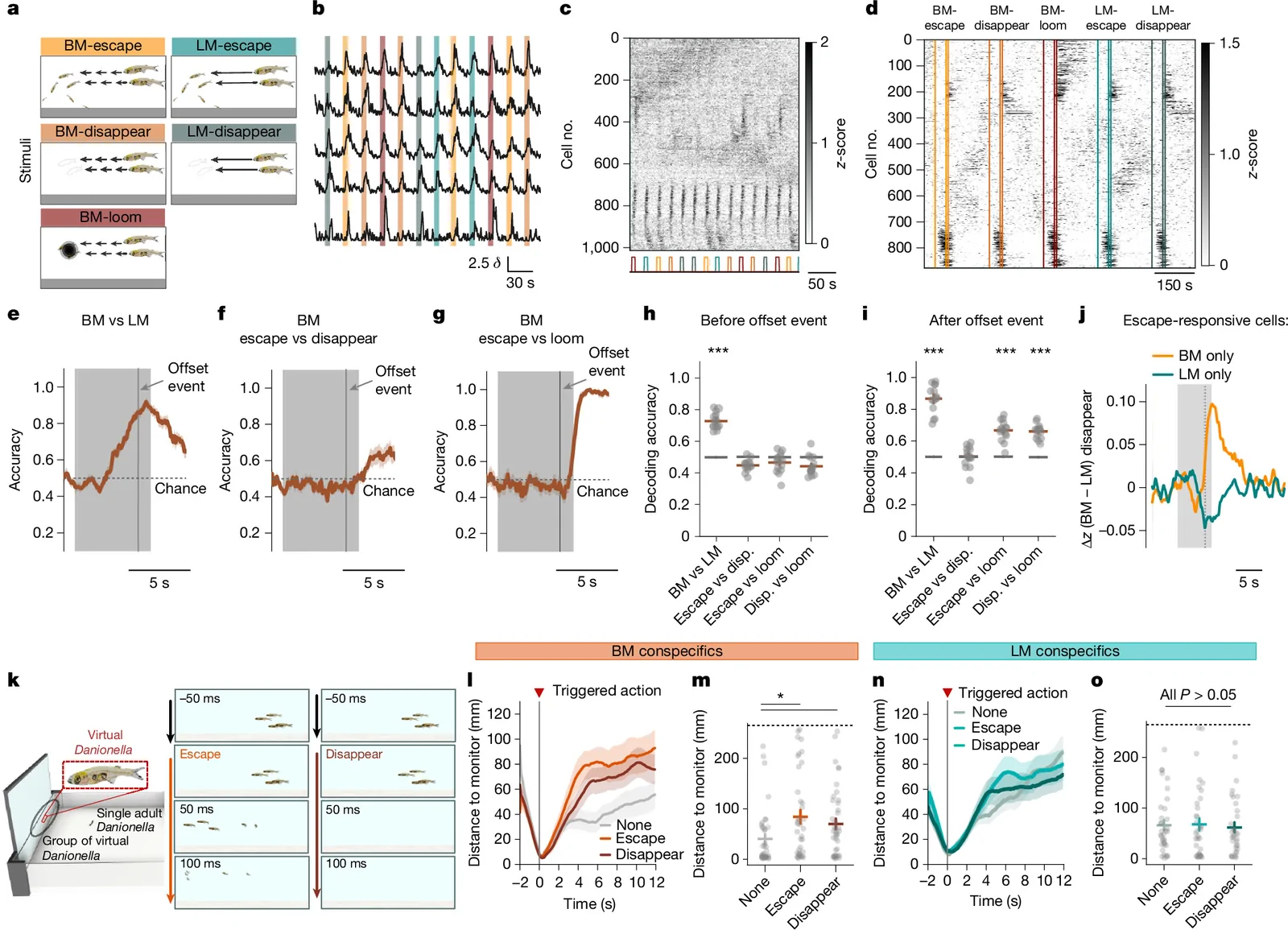
Neural detection of social offset enables social transmission in collective escape. **a**, Illustration of the five types of visual stimuli where virtual *D. cerebrum* moves with either linear motion (LM) or BM (burst-and-glide), followed by escape, disappearance, or a looming sphere stimulus. Note that there are three virtual fish in each stimulus, but two are illustrated here owing to spatial constraints. **b**–**d**, Example neural responses with colour blocks and lines showing the stimulus presentation time and type in **a**. **b**, Short epoch of stimulus-driven single neuron responses in an example fish over 30 s. **c**,**d**, Example raster map of all recorded cells (**c**; *n* = 1,015 cells) and trial-averaged responses from all responsive cells (**d**; *n* = 878 cells) in an example fish. **e**–**g**, Accuracy of support vector machine (SVM) models, 15 imaging sessions with at least 8 trials of each type. Grey shading marks the time of stimulus presentation. Chance level is calculated from the mean accuracy score of bootstrapped models. *n* = 15 (15 iterations of bootstrapped each). Models are trained to classify biological motion versus linear motion (**e**), escape versus disappearance action (**f**) and disappearance versus looming stimulus (**g**). **h**,**i**, Performance of SVM models, 15 sessions. Each dot represents the mean accuracy of a model trained with cells recorded in one session, taken over 2 s before (**h**) and after (**i**) the escape or disappearance (disp.) event. *n* = 15 sessions (15 iterations of bootstrapped each). Grey bars are the average performance of bootstrapped models. **j**, Mean *z*-scored response difference to disappearance in escape-specific responsive cells. *n* = 2,845 (BM-only) and 2,552 (LM-only) cells. **k**, Left, behaviour paradigm for virtual conspecifics-evoked escape. Right, example frames of the virtual *D. cerebrum* group on the monitor during a proximity-triggered escape (left) or disappearance (right). Images of virtual fish are pixelated, as they appear when displayed to real fish in experiments. **l**–**o**, Distance from monitor during sessions when virtual *D. cerebrum* were moving in biological motion (**l**,**m**) or linear motion (**n**,**o**). **l**,**n**, Virtual escape onset is marked with a grey line at 0 s. **m**,**o**, Mean distance is taken from 5 s to 12 s after the triggered action. Dotted line is the maximal distance (265 mm). **m**, *n* = 39 (no action), 39 (escape) and 41 (disappear) sessions. **o**, *n* = 35 (no action), 36 (escape) and 34 (disappear) sessions. Data are mean ± s.e.m. See Supplementary Table 1 for full statistics and sample sizes. *P < 0.05, ***P < 0.001. Fish image in **a**,**k** adapted from ref. ^59^, CC BY 4.0. Source data

**Extended Data Fig. 8:**
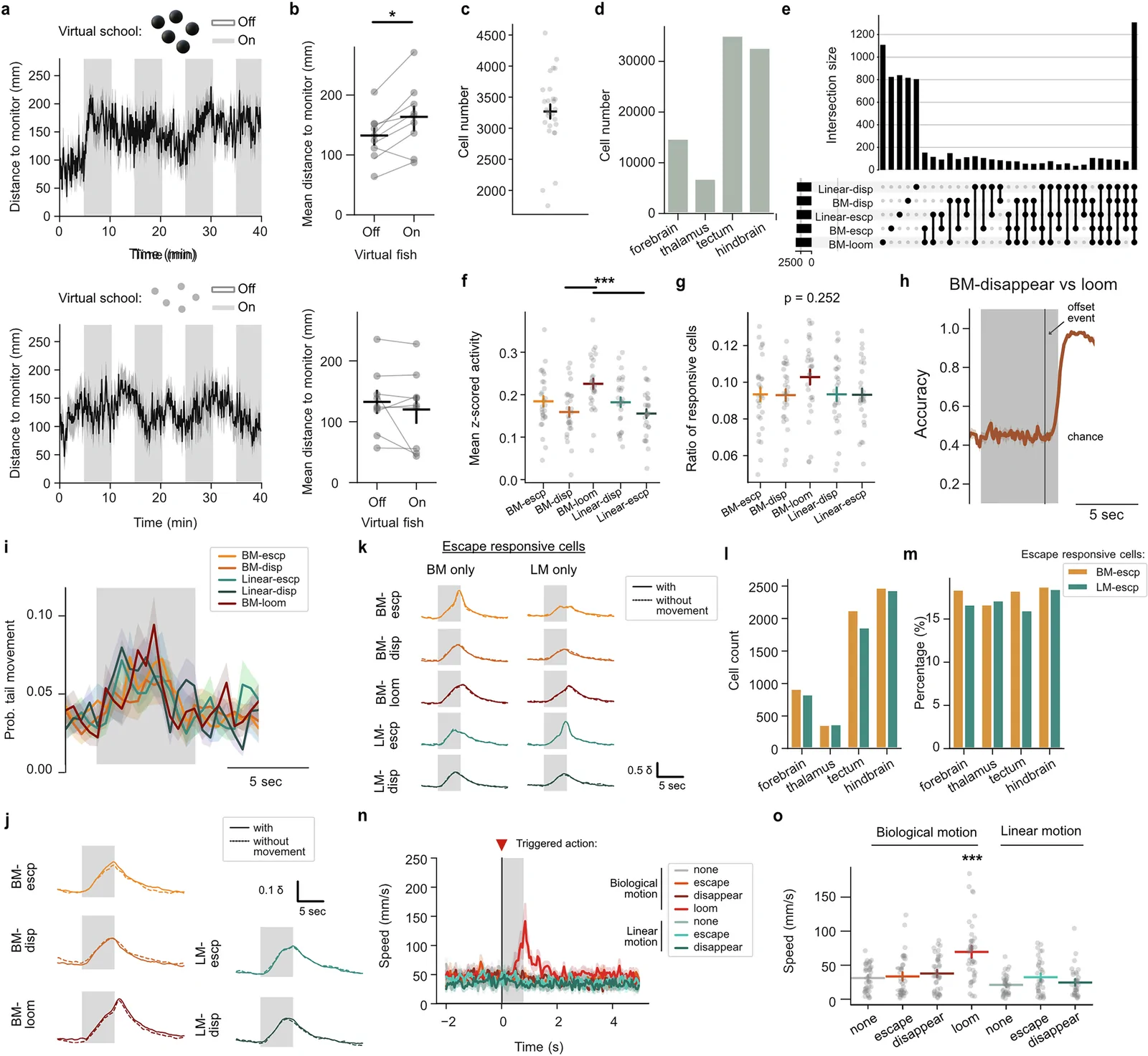
Behavioural and neural responses to visual stimuli with biological and linear motion. **a** Distance from the monitor over 40 min behavioural recording. Virtual group of objects was rendered for four five-minute-long segments, indicated by grey shading. Top: black dots. n = 9 fish. Bottom: smaller, gray dots. n = 9 fish. **b** Mean distance from the monitor. Top: black dots. n = 9 fish. Bottom: smaller, gray dots. n = 9 fish. Each dot is the mean distance of a fish during the 20 min with or without virtual conspecifics display. **c** Mean cell number recorded per imaging plane. n = 28 planes from 9 fish. (3269 ± 114). **d** Total cell number recorded across anatomical regions. n = 14751, 6864, 35092, and 32699 cells in the forebrain, thalamus, tectum, and hindbrain, respectively. **e** Intersection plot (UpSet) showing response combinations of neurons in all fish recorded. Neural responses overlap across linear and biological stimulus types. n = 7738 cells. **f** Maximal Z-scores of cells responsive to each stimulus type. Each dot represents the average of all cells in each recording session. n = 28 sessions. **g** Ratio of responsive cells to each stimulus type. Each dot represents the ratio of all cells recorded in one recording session. n = 28 sessions. **h** Accuracy of support vector machine (SVM) models over time. Gray shade marks the time of stimulus presentation. Chance level is calculated from mean accuracy score of bootstrapped models. N = 15 sessions (15 iterations of bootstrapped each). Models are trained to classify disappear vs. loom. **i** Probability of tail movement of the head-tethered fish during stimulus presentation (see Methods). Gray shade marks the time of stimulus presentation. **j** Mean z-scored response to different stimulus types in all fish recorded, separated by the presence of tail movement. Mean probability of tail movements during stimulus presentation is used to classify sessions with motion (n = 3185 trials, in solid lines) or without motion (n = 2290 trials, in dashed lines). **k** Mean z-scored response to different stimulus types in all fish recorded, separated by the presence of tail movement. Mean probability of tail movements during stimulus presentation is used to classify sessions with motion. n = 2845, 2552 cells that respond to escape that followed biological and linear motion only. **l** Total cell number across anatomically-defined regions: n = 919, 364, 2130, 2477 escape-responsive cells to only the biological motion, and 833, 374, 1865, 2440 cells for linear motion, in the forebrain, thalamus, tectum, and hindbrain, respectively. **m** Percentage of visually responsive cells in each anatomical region: 18.4, 16.7, 18.3, 18.7% of escape responsive cells to only the biological motion, and 16.7, 17.2, 16.0, 18.5% of cells to linear motion, in the forebrain, thalamus, tectum, and hindbrain, respectively. **n**,**o** Speed of single fish exposed to various virtual stimulus types. (**n**) Vertical black line indicates proximity-triggered action of the virtual *D. cerebrum*. Grey shading indicates duration of looming stimulus. **o** Median speed of fish during 5 to 12 sec post triggered action. n = 39, 39, 41, 42 sessions for virtual groups that continue their motion, escape, disappear, or a looming stimulus following biological motion. n = 35, 36, 34 sessions for virtual groups that continue their motion, escape, disappear following linear motion. *p < 0.05, **p < 0.01, ***p < 0.001. Data in (**c-m**) are from 28 sessions in 9 fish (1–5 imaging sessions, 1 plane per session). All data are shown in mean ± SEM. See Supplementary Table 1 for full statistics and sample sizes. Source data

To selectively respond to the actions of social partners, *D. cerebrum* must be able to recognize social partners before they escape. We propose that this is achieved by detecting their species-typical movement patterns: burst-and-glide biological motion. To test this, we recorded neural activity while presenting fish with realistic virtual *D. cerebrum* (Fig. 3a and Extended Data Fig. 5a), which drive robust stimulus engagement (Fig. 3b,c), unlike simple spheres (Extended Data Fig. 8a,b). We programmed three virtual fish to follow a defined trajectory in visual space, moving with either biological motion (burst-and-glide) or linear motion; after 5 s, stimuli either escaped, vanished, or stopped moving while a looming sphere appeared (Fig. 5a). These stimuli enable us to examine how the visual system distinguishes between the motion and actions of virtual conspecifics, and whether these visual neurons respond to any spatially localized visual threat.

We recorded from 3,269 ± 114 neurons in a 978 μm × 978 μm field of view in each of the 28 imaged planes (1 plane per imaging session, 1–5 sessions per fish, *n* = 9 fish; Extended Data Fig. 8c,d). Many neurons responded to each stimulus type, with the greatest response to the highly salient looming stimulus (Fig. 5b–d and Extended Data Fig. 8e–g). We trained linear classifiers to decode trial type from neural activity (Methods). We found that classifiers distinguished between patterns of virtual conspecific motion (biological versus linear motion), with increased classification accuracy during stimulus presentation and before the action (classification accuracy = 72.6%; Fig. 5e). However, classifiers were unable to effectively determine whether the observed action was an escape or disappearance (classification accuracy = 50.2%; Fig. 5f). By contrast, the classifier effectively decoded looming from escape or disappearance after biological motion (classification accuracy = 66.7% and 66.1%, respectively; Fig. 5g–i and Extended Data Fig. 8h), suggesting that these populations are not responding uniformly to all forms of visual danger. Motor activity did not modulate the neurons that responded to this stimulus set (Extended Data Fig. 8i–k), and these neurons were found across all brain regions imaged (Extended Data Fig. 8l,m). Cells that were responsive to the escape of virtual fish swimming with biological motion also responded to the disappearance of those fish, compared to the disappearance of virtual fish moving with linear motion (Fig. 5j).

Together, these results demonstrate that the visual system of *D. cerebrum* can identify objects that move with its species-typical burst-and-glide biological motion, and respond to the escape actions of those social partners. However, we find that these neurons do not distinguish between whether those social partners execute a kinematically realistic escape action or simply vanish.

## Social offset drives escape behaviour

To determine whether a fish’s visual perception of disappearing conspecifics affects their behaviour, we allowed individual freely moving *D. cerebrum* to engage with virtual fish models (as in Fig. 3a and Extended Data Fig. 5a). We programmed groups of five virtual fish to swim with either biological (burst-and-glide) or linear motion. When real *D. cerebrum* came within 16 mm of the groups, virtual fish either escaped, vanished, or did not alter their trajectory (Fig. 5k and  Methods).

We examined the behaviour of fish in response to the actions of virtual conspecifics swimming with biological motion, and found that either their escape or disappearance caused disengagement from the virtual group, resulting in them moving further than the control (no action, 40.4 mm; escape, 84.1 mm; disappear, 69.8 mm; Fig. 5l,m). There was no significant difference between behavioural responses to virtual fish escaping or disappearing (*P* = 0.546). By contrast, when virtual conspecifics moved with non-biological linear motion, real fish did not show a behavioural distinction between no action and their escape or disappearance (no action, 66.0 mm; escape, 65.6 mm; disappear, 61.8 mm; Fig. 5n,o). Notably, behavioural withdrawal from the offset of virtual conspecifics differs from the rapid escape from a direct threat (a loom; Extended Data Figs. 5d and 8n,o and Methods). These data demonstrate that *D. cerebrum* will rapidly disengage from conspecifics that escape or vanish, but only if those conspecifics move with biologically realistic burst-and-glide motion. This behavioural response to visual detection of escaping conspecifics can produce threat avoidance at the level of a small group, where escape amplitude decays with distance from directly threatened individuals^6,21,22^.

## Discussion

By analysing the perceptual and neural basis of escape behaviour in *D. cerebrum* groups, we found that the information transmission underlying this collective behaviour is mediated by the visual detection of conspecifics that suddenly escape or vanish. Our neural recordings indicate that this is accomplished by populations of visually responsive neurons, primarily in the optic tectum and thalamus, that are activated by the rapid disappearance of mobile visual objects with biological motion. Neural encoding of a social offset can be an effective means for animals to detect the escape actions of social partners, and thus execute an escape themselves. The ability to respond to the sensory experiences of one’s social partners (for instance, their detection of a predator that causes escape) is a benefit of living in a group, and exemplifies the ‘many eyes’ effect^1,3,25,26^. Our work provides evidence of how neural computation at the level of individual animals can produce emergent collective behaviours at the level of the group^3,11,12^. Although we focus on collective escape behaviour here, similar visual communication mechanisms may be at work during other collective behaviours, including in the context of navigation, foraging and decision-making^1–6^.

Our large-scale neural recordings demonstrate the central role of the optic tectum in *D. cerebrum*’s visual perception of escaping social partners. The tectum (or colliculus in mammals) enables salience-based attention and neuronal gain across vertebrates^16,17,36^. Whereas most studies have investigated responses to salient stimuli without social features, socially relevant visual stimuli are known to activate tectal neurons in fish^15,18^, mice^51^ and primates^52^. Therefore, the rapid visual detection of social actions with innate salience may be driven in part by conserved midbrain circuits across species. Here we classify social stimuli as those that move with biological motion, but biological motion alone may not always be sufficient to classify objects as being a social agent. Fish may use a combination of many sensory cues to identify conspecifics, including mechanosensory^53^, auditory^35,39^ or chemosensory^54^ information. Integration of multisensory cues is likely to contribute to many collective behaviours, including escape and predator avoidance.

It remains unknown whether the specific social-offset response that we observe here emerges from the on–off organization of presynaptic retinal ganglion cells^55^, or if it is generated by local circuits in the midbrain or thalamus^16,56,57^. In addition to the tectum, we found neurons for social action detection in the thalamus and telencephalon, which are associated with object detection, biological motion perception and social behaviour in zebrafish^18,50,56^. Interconnected tectal, thalamic and forebrain circuits^18,56^ may play an important part in classifying the species-typical movements of social partners to guide collective behaviour. Although we found distinctions between escape behaviours driven by looming or social stimuli, the mechanisms that we describe here need not be exclusive to social stimuli; offset responses could generalize to the rapid disappearance of other visual items that have captured an animal’s attention.

Visual attention to social actions may be particularly important for the adaptive behaviour of schooling fish. Aquatic animals commonly experience a more restricted visual range compared with terrestrial animals^19^, and *D. cerebrum* have been found in turbid waters with reduced visibility^14,34^. Therefore, individual animals may be particularly attuned to the actions of their neighbours, whose movements can inform observers of stimuli beyond their individual sensory range. As *D. cerebrum* are capable of high-speed escape movements^39^, an escaping social partner could rapidly vanish from the short reach of an individual’s field of view. The scattering behaviour that we observed here may be a consequence of these environmental features, as the socially transmitted signal for danger (disappearing conspecifics) may not provide the observer with sufficient information about the direction of the threat—only its imminence. For species that show collective escape waves^6,20–22,36^, social transmission of threat information may also include information about threat direction. Future studies can examine how group composition, ecological constraints and neurobiological mechanisms influence the diverse predatory avoidance strategies of animal collectives^1,11,12^.

Our results provide an instructive example of how the sensory ecology of an organism impacts the behavioural significance of its neural computations and perceptual abilities. *Danionella* will be a useful model clade to explore these concepts^34,58^, as different species occupy distinct ecological niches, such as clear versus turbid waters. Further insights into these natural behaviours will be gleaned by advancing experimental techniques for neural circuit dissection in *Danionella* and other emerging model systems capable of collective behaviour^12^.

## Methods

### Fish husbandry

*D. cerebrum*^13–15,33,35,54,60–63^ were raised and maintained in conventional zebrafish housing systems (Aquaneering; system water temperature 29 ± 0.5 °C, pH 7.0, conductivity 600 µS cm^−^^1^), under 14:10 h light:dark cycles, and were fed 2–3 times a day. *D. cerebrum* were bred in communal tanks of ~40–60 individuals, enriched with ~5 cm silicone tubes to encourage spawning. Eggs were collected during the first 1 h of daylight, and 1–2 h after morning and afternoon feeding. Embryos (0–5 days post-fertilization (dpf)) were raised in egg-water (5 mM NaCl, 169 μM KCl, 330 μM CaCl_2_, 161 μM MgSO_4_∙7H_2_O, and 0.15% methylene blue dissolved in reverse osmosis purified water) in an incubator at 29 ± 0.5 °C.

Larvae (5–14 dpf) were housed in static tanks in the housing systems and cocultured with L-type rotifers (*Brachionus plicatilis*), while water level was raised ~1 cm per day. At 21 dpf, the housing tank was connected to the water circulation system and fish were fed with artemia. At 8–10 weeks post-fertilization, *D. cerebrum* reach adulthood and become fertile. Wild-type *D. cerebrum* were provided by A. Douglass and B. Judkewitz. The transgenic line *Tg(elavl3:H2B-GCaMP6s)*^64^ was provided by B. Judkewitz.

The behavioural and imaging experiments with *D. cerebrum* were approved by the government authorities (US Animal Care, and Institutional Animal Care and Use Committee (IACUC), (USDA Registration Number 93-R-0437)) and carried out in accordance with the US federal and California state law to enforce the Animal Welfare Act (AWA) under the California Health and Safety Code.

### Behavioural experiments setups

All behavioural experiments used *D. cerebrum* that were at least 8 weeks old, and included both sexes. Behavioural recordings were conducted in a room with a portable electric heater to maintain water temperature between 28–30 °C. Data acquisition was automated and timestamped in BonsaiRx^65,66^ (https://bonsai-rx.org). All behavioural arenas were built with custom cut acrylic (black walls and transparent bottom with white styrene to diffuse bottom lit light sources) and encircled with black curtain to control luminance. Light and dark environments are controlled with a bottom-projected white or dark background (AnyBeam Pico Projector, HD301M1-H2).

Behaviours were illuminated with infrared light (CM-IR130-850NM, CMVision Technologies) from the bottom, and acquired at 121 Hz from a camera mounted above (FLIR Grasshopper, 33-534) with an 8 mm/F1.8 lens (Edmund Optics, 15-626) and long-pass filter (Edmund Optics, 12-767).

### Behavioural tracking and processing

Social LEAP-Estimates Animal Poses (SLEAP)^67^ was used to train models for tracking the location and posture of *D. cerebrum*. Each video was proofread after inference and identity tracking to create h5 files that contain positional information of all tracking nodes of each animal for the given behavioural session. The h5 files and a corresponding timestamped csv were processed with custom Python code (3.12) for further analysis (see Code availability). Models were trained with a nine-node skeleton covering the two eyes and evenly distributed points along the midline of the body and tail.

For each time point, animal centroid was defined as the *xy* coordinates of the middle point of the two lobes of the swim bladder. A nose point was defined as the centre point of the 2 eyes, and the orientation of the animal was defined as the direction of the vector pointing from the centroid to the nose point and smoothed by a 5-frame running average (~40 ms). Speed and angular speed of the animal was calculated by taking the derivatives of centroid coordinates and the heading direction and smoothed by a 10-frame running average (~80 ms), respectively. Distance and alignment of each fish to all its neighbours were identified for each time point. All *x* and *y* coordinates were then transformed from pixel to mm by measuring the arena edge, a known physical length, in Fiji. We saved csv files with the timestamps for each video frame and any stimulus presented. Python code aligned all arrays to the behaviour before further analysis.

### Visual loom-evoked escape assay

In the loom-evoked escape behaviours, *D. cerebrum* were left to habituate for 20 min in a square arena (265 × 265 mm) where two of the walls were lined with 10.1-inch LCD monitors (HAMTYSAN, 10.1 inch rendered at 1,024 × 600 pix). Each screen was mapped as a 14 × 20 cm window into a 3D virtual environment, eye perspective at centre point of area, 2 cm above the bottom, using BonVision package^66^. Each experiment consisted of 5× 2-min recordings, and at the 1-min mark a dark sphere of 4.3 cm would appear in one of the corners (*x* = ±30, *y* = ±15, *z* = 1.5 cm) (Extended Data Fig. 1b) and move linearly to the centre of the arena (*x* = 0, *y* = 0, *z* = 1.5 cm) within 3–6 s. With a total virtual displacement of 33.5 cm, the loom moved at a constant speed of 5.6–11.2 cm s^−1^, creating a length/velocity (*L*/*V*) ratio ranging from 384 to 769 ms. Both the starting position and moving duration were randomly determined. We separated each trial by 5-min intervals and used groups of 1, 2, 4 and 8 fish.

Baseline swimming speed in the 30 s before loom onset was fitted with Gaussian mixture model with four Gaussian components (determined by Bayesian information criterion) for single fish and fish in a group of four separately (Extended Data Fig. 1e). The boundary between the second and third components was defined as the threshold between low and high swimming speed. We defined escape latency as the first time point when a fish reached high speed if that time event occurred within 1 s after the end of loom (Extended Data Fig. 1f). We calculated the change in speed from looming stimulus by normalizing each fish’s speed 0.5 s after the end the stimulus to its mean speed 30 s prior to the onset of the loom (Fig. 1d,e). Travel distance before and after stimulus was calculated with integration of speed over 1 s (Fig. 1f and Extended Data Fig. 1g,l,m) window.

Turning behaviour was calculated by taking the absolute heading direction changes in 0.05-s bins, and plot it as probability density function (PDF) or cumulative distribution function over continuous 250-ms block (Extended Data Fig. 1n,o). Same statistical curves were plotted with 100-ms blocks to visualize the median turning angles (Fig. 1g). Inter-fish distance was calculated for each pair of fish within a group. The positions of each fish in the group were shifted by a random number independently to create the shuffled data used to calculate temporally shuffled inter-fish distance (Fig. 1h), and the trajectories of all fish were shuffled post end of loom to create chance level of spatially shuffled inter-fish distance (Extended Data Fig. 1r). Change in inter-fish distance was computed by subtracting the mean over 1 s before loom onset, and the mean at 0.5 s after the stimulus are used for statistical comparison (Fig. 1i).

Eye angle tracing of the loom was performed via two-dimensional ray-casting. In each video frame during loom stimulus presentation, we calculated the coordinates of the virtual sphere, and drew angles from the fish’s midpoint (between the two eyes) to the edges of the 4.3 cm sphere. The angular size of the loom is the absolute angle difference between the vectors to the left and right side of the sphere (Fig. 2a,b, Extended Data Fig. 4a,b). Speed from 6 s before the threshold was used as baseline to calculate changes (Fig. 2b,c).

We created Boolean arrays to identify when each fish first senses the loom at the threshold angle of 7°. These arrays were resampled to align trials of different lengths (speeds of 5.6–11.2 cm s^−1^), and construct the cumulative probability curve of reaching loom threshold (Fig. 2e and Extended Data Fig. 4b). Using trials aligned from loom start to loom end, we identified the time through the trial where 50% of group members pass the angle threshold. This time point was used to assign individual and group information status in the subsequent analysis. For individuals, they were labelled as ‘early-informed’ if loom reaches critical angle at an earlier time than this cut-off. Otherwise, individuals were labelled as ‘late-informed’, including those that never reached critical angles and did not have a valid reaching time (that is, the angle of the loom never reached 7°). The mean of the threshold-reaching time in a group was also compared to the same cut-off to assign the group as ‘early group’ or ‘late group’ (Fig. 2e). Therefore, early groups contained more early-informed individuals, and vice versa. To compare behaviour across trials, we calculated the average speed in time bins 5% of the original trial length.

### Mechanosensory tap-evoked escape assay

In the tap-evoked escape behaviours, *D. cerebrum* were left to habituate for 20 min prior to the experiment in a 350 mm circular arena filled with 1 l of system water^15^. Each experiment consisted of 5 repetitions of 2-min recordings, where an actuator (Adafruit Industries, TAU0730TM-14) mounted beneath one corner of the arena was moved by a 300-ms voltage pulse that delivered a mechanical tap at the 1-min mark. Each trial was separated by 7 min, and experiments included groups of 1, 2, 4 or 8 fish. For the light–dark experiments of tap escape, groups of 1 or 4 fish from a different cohort were used. Behaviours were run with either a white or black background bottom projection.

Baseline swimming speed in the 30 s before tap onset was fitted with Gaussian mixture model with four Gaussian components (determined by Bayesian information criterion) for single fish and fish in groups of four separately (Extended Data Fig. 2g). The boundary between the second and third components defined the threshold between low and high swimming speed. Escape latency within 1 s after the end of tap was identified as the first time point when a fish reached high speed (Extended Data Fig. 2h). The mean speed in the 30 s prior to the tap was used as the baseline for each individual fish to calculate how their speed changed 0.5 s after the tap (Extended Data Fig. 2d,e). Travel distance before and after the stimulus was calculated by integrating the speed over a 1 s (Extended Data Fig. 3d,e,i,j) window.

Turning behaviour was calculated by taking the absolute heading direction changes in 0.05-s bins, and plot it as PDF or cumulative distribution function over continuous 250-ms block (Extended Data Fig. 2n,o). Same statistical curves were plotted with 100-ms blocks to visualize the median turning angles (Extended Data Fig. 2p).

Inter-fish distance was calculated for each pair of fish within a group. The positions of each fish in the group were shifted by a random number independently to create the shuffled data used to calculate temporally shuffled inter-fish distance (Extended Data Fig. 2i,k), and the trajectories of all fish were shuffled post end of tap to create chance level of spatially shuffled inter-fish distance (Extended Data Fig. 2m). Change in inter-fish distance was computed by subtracting the mean over 1 s before tap onset, and the mean at 0.5 s after the stimulus are used for statistical comparison (Extended Data Figs. 2j and 3l).

### Virtual conspecifics engagement and social escape assay

We created a 3D virtual reality (VR) environment using Panda3D (https://www.panda3d.org/), and populated it with five virtual male *D. cerebrum* (Extended Data Fig. 5a) modelled in Blender (v3.5.1). The *Danionella* model was adapted from an image in Kadobianskyi et al.^59^. The VR scene was captured by a virtual camera positioned in VR space such that the virtual *D. cerebrum* appeared on the bottom third of the monitor. These virtual conspecifics appeared on a single monitor, with the opposing monitor displaying an empty background. Each VR *D. cerebrum* appeared ~12 mm in length on the display and had animated tail movements that coincided with the burst phase of their burst-and-glide kinematics (Extended Data Fig. 5b). The virtual school was programmed to swim along the width of the monitor by translating their *x* position in VR space, with small, random deviations in *z* to simulate slight changes in depth. The school always remained in the field of view of the VR camera, and therefore appeared on the monitor, as each fish executed a 180° turn when they reached the boundary of environment.

The environment was rendered on the monitors of the same square arena used in loom-evoked escape assay (Fig. 3a). To establish whether the sight of our virtual school attracted real fish, we first implemented an open loop paradigm (Fig. 3b,c). Here, a single *D. cerebrum* was left to habituate for 5 min in a square arena before being shown 4× 5-min no stimulus followed by 5-min stimulus on, with 5 swimming virtual *D. cerebrum*. At the end of each stimulus-on period, all virtual fish were assigned a random escape heading spanning 150° towards the orientation of the virtual camera so they rapidly swam away from the virtual camera into the horizon of the VR environment (Fig. 3d). These actions were not contingent on the position of the real fish with respect to the virtual fish.

For the social escape assays, we implemented a closed-loop paradigm wherein the proximity of the real fish to the virtual school triggered an escape. For this, we again allowed a single *D. cerebrum* to habituate to the environment for 5 min. After 1 min of the 5-fish virtual school being displayed on the monitor, the test fish’s engagement with the virtual school (test fish distance to centre of virtual school <16 mm) triggered either an escape or disappearance of VR fish depending on the experiment (Figs. 3d,e and 5k and Extended Data Fig. 5c). The overhead camera served to track the centroid position of the fish in real time using the KNN background subtractor function from the openCV library. The number of escaping or disappearing virtual fish was randomized for experiments with differing numbers of escapees, with each fish experiencing a 1-, 3- and 5-fish-escape event from the group of 5 total in the school (Fig. 3f,g). Each trial was separated by 60 s wherein we displayed an empty background after the escape. The virtual fish then re-appeared on the screen and the process repeated for a total of three trials per fish. For trials examining the response to linear movement of the virtual school, we programmed the virtual fish to move at the average velocity of a burst-glide cycle and removed the tail movement animation. In the case of disappearing fish, they vanished at the onset of ‘escape’. We used a similar closed-loop trigger to initiate a black looming stimulus that emerged from the centre of the virtual school (Extended Data Fig. 8n,o). The looming stimulus expanded hyperbolically over the span of 0.75 s, averaging 54° s^−1^, to a final size of 50 mm on the LCD monitor.

### Fish immobilization and holding chamber for in vivo two-photon calcium imaging

We modified the head-tethered preparation and holding chamber design from Zada et al. 2024^15^. In brief, adult *D. cerebrum* were transiently anaesthetized with tricaine (120 mg l^−1^ MS-222 for 1–2 min) before they were removed from water and placed ventral side up on a 24 × 30 × 1 mm coverslip (12545B, Fisher Scientific). Two pieces of pre-cured SYLGARD wedges were fixed on the coverslip with UV-cured plastic (Bondic) to support the fish body from behind the eyes. 5–10% low-melting point agarose (UltraPureTM LMP Agarose, Invitrogen) was added to cover the body from swim bladder to the proximal tail, and on the side of head in front of the SYLGARD pieces. Another horizontal strip of UV-cured plastic was added to connect the coverslip and the agarose for more stability. Agarose from the side of the eyes, gills, and the tip of the tail were removed with a scalpel. Once all components were dried, the fish was placed back into a holding dish and ventilated with oxygenated water to ensure gill movement. Fish that recovered and showed spontaneous gill ventilation were then moved into the holding chamber under the two-photon microscope.

The triangular chamber was built from two monitors (HAMTYSAN, 7-inch 800 × 480 LCD). The two screens occupied ~260 ° of visual space in azimuth (130 ° each side, with ~10 ° gap in the front, and ~90 ° gap behind the fish), and ~105 ° of visual space in elevation (52.5 ° below and above). The chamber was filled with fish housing water (28 °C, ~500 ml). The water level was maintained ~1 cm above the midline of monitor height (10 cm) by gravity flow of fresh fish housing water and a custom vacuum made from a bent borosilicate electrode (WPI) constantly removing excess water throughout the duration of the experiment.

The back wall and floors were made of transparent acrylic for IR lights and recording camera access. A 4.5 mm column of clear acrylic was used as a pedestal to position head-fixed fish at a standard positioning within this arena (intersection of each screen’s midpoint). Coverslips were suspended from the acrylic pedestal using two 3 ×1 mm magnets (Ethcool) that was embedded into the top of the pedestal and another one added when anchored.

### Visual stimuli

#### Dot stimuli

BonsaiRx was used to record the timing of microscope frame acquisitions, and display visual stimuli. Visual stimuli were displayed on the screens using cube mapping in BonVision^66^, where each screen displayed a viewport in a virtual environment. The virtual environment was composed of a 6,000 mm^2^ plane with a smoothed white-noise pattern (‘seafloor’). The fish’s position in this VR world was in the centre (0,0 in *xy* coordinates), 10 mm above the seafloor.

For nonsocial stimuli, a dark sphere was programmed to appear in the front of fish in the virtual environment. After 1 s, it either moved horizontally to the periphery (linear motion stimulus) or moved toward the virtual camera (thus, expanding in size; loom stimulus) over 5 s (Extended Data Fig. 6b). For the social stimuli, behavioural recordings of swimming and escaping *D. cerebrum* from both top-down and lateral view were processed to obtain realistic position changes of fish in 3D space (Extended Data Fig. 6a). Behaviour tracking (121 and 200 Hz for the top-down and lateral side view) was resampled to the refresh rate of monitors (60 Hz) and applied to spheres. The diameter and distance of the spheres in the virtual environment were calibrated to match their size with live fish. The two smaller dots swam from the back to front of the head-tethered fish in the VR environment over 6 s (swim stimulus) or executed a downward and outward escape at the 3 s mark and kept swimming farther (Fig. 4d and Extended Data Fig. 6a,b). All stimuli within a session were delivered to one visual field (left or right).

#### Virtual *D. cerebrum* stimuli

A custom Python script was used to record the timing of microscope frame acquisitions and display visual stimuli. To simulate the presence of conspecifics, we constructed a 3D environment using Panda3D, positioning two virtual cameras to match the perspective of the head-tethered fish. A virtual school comprising three *D. cerebrum* were used, populating one visual field (left or right).

For stimulus types with biological motion, the virtual school moved with realistic burst-and-glide kinematics (rapidly accelerating and then exponentially decelerating at ~1 Hz, Extended Data Fig. 5b) and with animated tail movements that corresponded with the burst phase of motion. For stimulus types with linear motion, the virtual school moved with a constant velocity and without animated tail movements. The start and end position of each fish, as well as the average speed, were matched to stimuli swimming with biological motion. Movement in each trial lasted for 5 s, which corresponded to 4 burst-glide cycles, and was followed by either an escape or disappearance of the virtual fish, or the emergence of a looming stimulus. Escape trajectories spanned 45–135° away from the heading of the head-tethered fish. That is, the virtual fish veered to the left when they were displayed on the left screen, and they veered to the right when displayed on the right screen. To avoid contaminating our imaging path with green/blue light from the visual display, we lined the LCD monitors with red gel light filters (LILIYA, 8.5 × 11 inch cut to size) and red-shifted the virtual environment rendering with a graphic shader (Background colour is set as Light Coral (hex code f08080); ambient light RGB set to 1/0.5/0.5 to bias the light reflection from the seafloor and objects toward longer wavelength).

For both experiments, each recording session started with a 1–2 min baseline with only the seafloor background scene visible. We then used a trial structure to present each of the stimulus types to head-tethered fish for 10 trials each in a pseudorandomized order, with randomized 14–16 s inter-trial intervals. All stimuli were displayed underneath the water surface of the recording arena and on the same side of the monitor within the recording session (15–20 min). Concurrently, we recorded the tails of these fish using a FLIR Grasshopper camera (33-534) affixed with a long-pass filter (Edmund Optics, 12-767) at 30–120 Hz. Each video frame was also timestamped by the Bonsai or Python script for post hoc data synchronization.

### Two-photon calcium imaging

Two-photon microscopy was performed with a ThorLabs Bergamo II multiphoton microscope controlled by ThorImageLS 4.3 and illuminated by an fs-pulsed 80-MHz Ti:S laser (MaiTai DeepSee; SpectraPhysics). GCaMP6s fluorescence was imaged with a 16×/0.8 W Nikon objective at an excitation wavelength of 930 nm and ≤10 mW excitation power. Frames of 1,024 × 1,024 pixels covered a field-of-view of 978 × 978 μm.

We imaged fish at a diagonal, to include as much of the brain in our field of view as possible^15,18^. Depth of the imaging plane was determined by maximizing the number of cells in the optic tectum within the field of view. Dual-plane or single-plane imaging sessions were conducted for each fish if it maintained healthy spontaneous movement for the entirety of the experiments, where the stimuli came from each side of the visual field twice each. For two-plane imaging, we used fast *z* mode in ThorImageLS 4.3 to move across 50–90 μm at 5 Hz. For the single-plane imaging, a 2–3 frame average was used to produce a final effective imaging rate at 5–7.6 Hz.

### Imaging analysis

We only proceeded with experiments where fish remained healthy at the end of recording and did not have substantial *z*-movements or drift during recording. For sessions with detected shifts in *z*, we trimmed the *z*-shifted frames if the remaining uncorrupted experimental length was more than half of the original time series, and analysed neural activity in this epoch.

Imaging data were motion corrected in Suite2p^68^, followed by cell extraction. Time series were inspected using Rastermap^69^ to ensure there was no *z*-motion contamination or slow drift, and we only included neurons with continuous measurements and which were classified as a cell by Suite2p (“iscell”=1). A cellpose^70^ classifier was trained for cell extraction. The anatomical region where each neuron is located was further classified with manually defined boundaries; cells outside these boundaries were not included for region-specific analyses. We excluded the first 5% of each imaging trial to avoid the influence of sound-evoked activity upon the initiation of scanning. Stimulus timing and calcium traces were aligned and resampled to a common 10 Hz sampling rate for further analysis. Then the fluorescence traces were detrended, smoothed with a 1-s rolling mean, and *z*-scored. The *z*-scored fluorescence and the median *xy* coordinates for each cell within these boundaries were used for further analysis.

We identified stimulus-responsive cells by conducting a *t*-test between the mean z-scored fluorescence during the 6-s stimulus and the mean *z*-scored fluorescence in the 5-s baseline pre-stimulus. Neurons were identified as responsive to a certain stimulus type if the significance is *P* < 0.05. Offset cells are defined by peak response time (1) at or later than 6 s to biological motion (BM) stimulus onset and (2) between 3 and 6 s to BM-escape onset. Among them, those peaked at or later than 6 s to the linear stimulus onset are categorized as general-offset cells, and those peaked before 6 s are categorized as social-offset cells. For the support vector machine (SVM) models, we only used experiments where there were at least 8 trials for all four stimulus types. The decoders were trained on the principal components (those that explain 90% of variance) of neural activity from all responsive cells in each recording session. For longer sessions, 8 trials were randomly selected for each stimulus type and split with a stratified K-fold cross-validator (eightfold). Twenty-five iterations of training and testing were performed.

Tail motion was assessed by measuring the number of pixels corresponding to tail movement in each video frame during imaging sessions. We processed each video frame using three consecutive functions from the OpenCV library: First, we applied a uniform Gaussian blur to each frame (cv2.GaussianBlur). Second, we dilated the resulting image (cv2.MORPH_OPEN). Third, we used the KNN background subtractor function to identify and sum moving foreground pixels that corresponded to the moving tail (cv2.createBackgroundSubtractorKNN). To compare movement across videos, we *z*-scored the tail movement of each video comparing each frame to a 15 s rolling mean. Time points with a *z*-score exceeding 2 were classified as motion. We averaged significant movement across fish using a 500-ms bin, and each trial was categorized as a motion trial if the mean of significance movement during stimulus presentation exceeds zero.

### Statistics

Groups were tested for normality using the Shapiro–Wilk test. Non-parametric tests (Mann–Whitney *U*, Kruskal–Wallis *H*) were conducted if the Shapiro–Wilk *P* < 0.05 for any group. Otherwise, parametric tests were used. Post hoc tests were conducted if main effect was *P* < 0.05. All post hoc tests (Dunn’s test for non-parametric data, or *t*-tests for parametric data) were corrected for multiple comparisons with a Bonferroni or Holm correction. For nested data (neurons within imaging session within experimental groups), we determined the significance of pairwise comparisons using linear mixed-effects models, with imaging session identity (id) as a random variable. Exact tests and *P* values are reported in Supplementary Table 1.

### Reporting summary

Further information on research design is available in the Nature Portfolio Reporting Summary linked to this article.

## Online content

Any methods, additional references, Nature Portfolio reporting summaries, source data, extended data, supplementary information, acknowledgements, peer review information; details of author contributions and competing interests; and statements of data and code availability are available at https://doi.org/10.1038/s41586-026-11041-1.

## Supplementary information

Supplementary Table 1 Statistics and sample sizes.

Reporting Summary

Peer Review File

## Source data

Source Data Fig. 1

Source Data Fig. 2

Source Data Fig. 3

Source Data Fig. 4

Source Data Fig. 5

Source Data Extended Data Fig. 1

Source Data Extended Data Fig. 2

Source Data Extended Data Fig. 3

Source Data Extended Data Fig. 4

Source Data Extended Data Fig. 5

Source Data Extended Data Fig. 6

Source Data Extended Data Fig. 7

Source Data Extended Data Fig. 8

## Data Availability

Data are available at https://doi.org/10.6084/m9.figshare.30223705 (ref. ^71^).  Source data are provided with this paper.

## Code availability

Code is available at https://github.com/lblabucsd/Yu_etal_2026/.

## References

[1] Krause, J. & Ruxton, G. D. *Living in Groups* (Oxford Univ. Press, 2002).

[2] Sumpter, D. J. T. The principles of collective animal behaviour. *Philos. Trans. R. Soc. B* **361**, 5–22 (2006). 10.1098/rstb.2005.1733 PMC1626537 16553306

[3] Couzin, I. D. Collective cognition in animal groups. *Trends Cogn. Sci.* **13**, 36–43 (2009). 10.1016/j.tics.2008.10.002 19058992

[4] Couzin, I. D., Krause, J., Franks, N. R. & Levin, S. A. Effective leadership and decision-making in animal groups on the move. *Nature* **433**, 513–516 (2005). 10.1038/nature03236 15690039

[5] Strandburg-Peshkin, A. et al. Visual sensory networks and effective information transfer in animal groups. *Curr. Biol.* **23**, R709–R711 (2013). 10.1016/j.cub.2013.07.059 PMC4780851 24028946

[6] Rosenthal, S. B., Twomey, C. R., Hartnett, A. T., Wu, H. S. & Couzin, I. D. Revealing the hidden networks of interaction in mobile animal groups allows prediction of complex behavioral contagion. *Proc. Natl Acad. Sci. USA* **112**, 4690–4695 (2015). 10.1073/pnas.1420068112 PMC4403201 25825752

[7] Nagy, M., Akos, Z., Biro, D. & Vicsek, T. Hierarchical group dynamics in pigeon flocks. *Nature* **464**, 890–893 (2010). 10.1038/nature08891 20376149

[8] Sumpter, D., Buhl, C., Biro, D. & Couzin, I. Information transfer in moving animal groups. *Theory Biosci.* **127**, 177–186 (2008). 10.1007/s12064-008-0040-1 18458976

[9] Gallup, A. C. et al. Visual attention and the acquisition of information in human crowds. *Proc. Natl Acad. Sci. USA* **109**, 7245–7250 (2012). 10.1073/pnas.1116141109 PMC3358867 22529369

[10] Bain, N. & Bartolo, D. Dynamic response and hydrodynamics of polarized crowds. *Science* **363**, 46–49 (2019). 10.1126/science.aat9891 30606837

[11] Hein, A. M. Ecological decision-making: From circuit elements to emerging principles. *Curr. Opin. Neurobiol.* **74**, 102551 (2022). 10.1016/j.conb.2022.102551 35576827

[12] Yu, J.-H., Napoli, J. L. & Lovett-Barron, M. Understanding collective behavior through neurobiology. *Curr. Opin. Neurobiol.* **86**, 102866 (2024). 10.1016/j.conb.2024.102866 PMC11439442 38852986

[13] Schulze, L. et al. Transparent Danionella translucida as a genetically tractable vertebrate brain model. *Nat. Methods* **15**, 977–983 (2018). 10.1038/s41592-018-0144-6 30323353

[14] Britz, R., Conway, K. W. & Rüber, L. The emerging vertebrate model species for neurophysiological studies is *Danionella cerebrum*, new species (Teleostei: Cyprinidae). *Sci. Rep.* **11**, 18942 (2021). 10.1038/s41598-021-97600-0 PMC8460714 34556691

[15] Zada, D. et al. Development of neural circuits for social motion perception in schooling fish. *Curr. Biol.* **34**, 3380–3391.e5 (2024). 10.1016/j.cub.2024.06.049 PMC11419698 39025069

[16] Isa, T., Marquez-Legorreta, E., Grillner, S. & Scott, E. K. The tectum/superior colliculus as the vertebrate solution for spatial sensory integration and action. *Curr. Biol.* **31**, R741–R762 (2021). 10.1016/j.cub.2021.04.001 PMC8190998 34102128

[17] Knudsen, E. I. Neural circuits that mediate selective attention: a comparative perspective. *Trends Neurosci.* **41**, 789–805 (2018). 10.1016/j.tins.2018.06.006 PMC6204111 30075867

[18] Kappel, J. M. et al. Visual recognition of social signals by a tectothalamic neural circuit. *Nature* **608**, 146–152 (2022). 10.1038/s41586-022-04925-5 PMC9352588 35831500

[19] MacIver, M. A. & Finlay, B. L. The neuroecology of the water-to-land transition and the evolution of the vertebrate brain. *Philos. Trans. R. Soc. B* **377**, 20200523 (2022). 10.1098/rstb.2020.0523 PMC8710882 34957852

[20] Procaccini, A. et al. Propagating waves in starling, Sturnus vulgaris, flocks under predation. *Anim. Behav.* **82**, 759–765 (2011).

[21] Herbert-Read, J. E., Buhl, J., Hu, F., Ward, A. J. W. & Sumpter, D. J. T. Initiation and spread of escape waves within animal groups. *R. Soc. Open Sci.* **2**, 140355 (2015). 10.1098/rsos.140355 PMC4448869 26064630

[22] Poel, W. et al. Subcritical escape waves in schooling fish. *Sci. Adv.* **8**, eabm6385 (2022). 10.1126/sciadv.abm6385 PMC9217090 35731883

[23] Hein, A. M., Gil, M. A., Twomey, C. R., Couzin, I. D. & Levin, S. A. Conserved behavioral circuits govern high-speed decision-making in wild fish shoals. *Proc. Natl Acad. Sci. USA.* **115**, 12224–12228 (2018). 10.1073/pnas.1809140115 PMC6275531 30420510

[24] Ward, A. J. W., Herbert-Read, J. E., Sumpter, D. J. T. & Krause, J. Fast and accurate decisions through collective vigilance in fish shoals. *Proc. Natl Acad. Sci. USA.* **108**, 2312–2315 (2011). 10.1073/pnas.1007102108 PMC3038776 21262802

[25] Lima, S. L. Collective detection of predatory attack by social foragers: fraught with ambiguity? *Anim. Behav.* **50**, 1097–1108 (1995).

[26] Davidson, J. D. et al. Collective detection based on visual information in animal groups. *J. R. Soc. Interface.* **18**, 20210142 (2021). 10.1098/rsif.2021.0142 PMC8261228 34229461

[27] Pacher, K. et al. Better and faster decisions by larger fish shoals in the wild. *Sci. Adv.* **11**, eadt8600 (2025). 10.1126/sciadv.adt8600 PMC12309664 40737396

[28] Sten, T. H., Li, R., Otopalik, A. & Ruta, V. Sexual arousal gates visual processing during Drosophila courtship. *Nature* **595**, 549–553 (2021). 10.1038/s41586-021-03714-w PMC8973426 34234348

[29] Cowley, B. R. et al. Mapping model units to visual neurons reveals population code for social behaviour. *Nature* **629**, 1100–1108 (2024). 10.1038/s41586-024-07451-8 PMC11136655 38778103

[30] Forli, A. & Yartsev, M. M. Hippocampal representation during collective spatial behaviour in bats. *Nature* **621**, 796–803 (2023). 10.1038/s41586-023-06478-7 PMC10533399 37648869

[31] Akinrinade, I. et al. Evolutionarily conserved role of oxytocin in social fear contagion in zebrafish. *Science* **379**, 1232–1237 (2023). 10.1126/science.abq5158 36952426

[32] Puri, P. et al. Collective motion in *Danionella* emerges from discrete copying interactions. *Phys. Rev. Lett.* **137**, 098403 (2026). 10.1103/rlkf-vwlc 42726957

[33] Hoffmann, M., Henninger, J., Veith, J., Richter, L. & Judkewitz, B. Blazed oblique plane microscopy reveals scale-invariant inference of brain-wide population activity. *Nat. Commun.* **14**, 8019 (2023). 10.1038/s41467-023-43741-x PMC10695970 38049412

[34] Bass, A. H. & Perelmuter, J. T. *Danionella* fishes. *Nat. Methods* **21**, 1767–1769 (2024). 10.1038/s41592-024-02433-0 39384984

[35] Henninger, J. et al. Brain-wide hierarchical and sexually dimorphic tuning for social vocalizations. Preprint at *bioRxiv* 10.64898/2026.03.04.709502 (2026).

[36] Branco, T. & Redgrave, P. The neural basis of escape behavior in vertebrates. *Annu. Rev. Neurosci.* **43**, 417–439 (2020). 10.1146/annurev-neuro-100219-122527 32259462

[37] Escobedo, R. et al. A data-driven method for reconstructing and modelling social interactions in moving animal groups. *Philos. Trans. R. Soc. B* **375**, 20190380 (2020). 10.1098/rstb.2019.0380 PMC7423377 32713309

[38] Lin, G. et al. Experimental evidence of stress-induced critical state in schooling fish. *PRX Life* **3**, 033018 (2025).

[39] Veith, J. et al. The mechanism for directional hearing in fish. *Nature* **631**, 118–124 (2024). 10.1038/s41586-024-07507-9 PMC11222163 38898274

[40] Romey, W. L., Smith, A. L. & Buhl, C. Flash expansion and the repulsive herd. *Anim. Behav.* **110**, 171–178 (2015).

[41] Storms, R. F., Carere, C., Zoratto, F. & Hemelrijk, C. K. Complex patterns of collective escape in starling flocks under predation. *Behav. Ecol. Sociobiol.* **73**, 10 (2019). 10.1007/s00265-018-2609-0 PMC6404399 30930523

[42] Hamilton, W. D. Geometry for the selfish herd. *J. Theor. Biol.* **31**, 295–311 (1971). 10.1016/0022-5193(71)90189-5 5104951

[43] Hein, A. M. et al. The evolution of distributed sensing and collective computation in animal populations. *eLife* **4**, e10955 (2015). 10.7554/eLife.10955 PMC4755780 26652003

[44] McKee, A. & McHenry, M. J. The strategy of predator evasion in response to a visual looming stimulus in zebrafish (*Danio rerio*). *Integr. Org. Biol.* **2**, obaa023 (2020). 10.1093/iob/obaa023 PMC7750966 33791564

[45] Dunn, T. W. et al. Neural circuits underlying visually evoked escapes in larval zebrafish. *Neuron* **89**, 613–628 (2016). 10.1016/j.neuron.2015.12.021 PMC4742414 26804997

[46] Fahimipour, A. K. et al. Wild animals suppress the spread of socially transmitted misinformation. *Proc. Natl Acad. Sci. USA* **120**, e2215428120 (2023). 10.1073/pnas.2215428120 PMC10083541 36976767

[47] Hemelrijk, C. K., Costanzo, A., Hildenbrandt, H. & Carere, C. Damping of waves of agitation in starling flocks. *Behav. Ecol. Sociobiol.* **73**, 125 (2019). 10.1007/s00265-015-1891-3 PMC4564680 26380537

[48] Krauzlis, R. J., Bogadhi, A. R., Herman, J. P. & Bollimunta, A. Selective attention without a neocortex. *Cortex* **102**, 161–175 (2018). 10.1016/j.cortex.2017.08.026 PMC5832524 28958417

[49] Larsch, J. & Baier, H. Biological motion as an innate perceptual mechanism driving social affiliation. *Curr. Biol.* **28**, 3523–3532.e4 (2018). 10.1016/j.cub.2018.09.014 30393036

[50] Lifshitz, I. et al. Distinct distributed neural dynamics predict pallium-dependent social approach. *Nat. Commun.* **17**, 4848 (2026). 10.1038/s41467-026-71666-8 PMC13222866 41957349

[51] Solié, C. et al. Superior colliculus to VTA pathway controls orienting response and influences social interaction in mice. *Nat. Commun.* **13**, 817 (2022). 10.1038/s41467-022-28512-4 PMC8831635 35145124

[52] Lu, X. et al. Detecting biological motion signals in human and monkey superior colliculus: a subcortical-cortical pathway for biological motion perception. *Nat. Commun.* **15**, 9606 (2024). 10.1038/s41467-024-53968-x PMC11542025 39505906

[53] Anneser, L. et al. The neuropeptide Pth2 dynamically senses others via mechanosensation. *Nature* **588**, 653–657 (2020). 10.1038/s41586-020-2988-z 33268890

[54] Penalva-Tena, A. et al. Oxytocin-mediated social preference and socially reinforced reward learning in the miniature fish *Danionella cerebrum*. *Curr. Biol.* **35**, 363–372.e3 (2025). 10.1016/j.cub.2024.11.037 39732054

[55] Baden, T. The vertebrate retina: a window into the evolution of computation in the brain. *Curr. Opin. Behav. Sci.* **57**, 101391 (2024). 10.1016/j.cobeha.2024.101391 PMC11183302 38899158

[56] Heap, L. A. L., Vanwalleghem, G., Thompson, A. W., Favre-Bulle, I. A. & Scott, E. K. Luminance changes drive directional startle through a thalamic pathway. *Neuron* **99**, 293–301.e4 (2018). 10.1016/j.neuron.2018.06.013 29983325

[57] Salay, L. D., Ishiko, N. & Huberman, A. D. A midline thalamic circuit determines reactions to visual threat. *Nature* **557**, 183–189 (2018). 10.1038/s41586-018-0078-2 PMC8442544 29720647

[58] Jourjine, N. & Hoekstra, H. E. Expanding evolutionary neuroscience: insights from comparing variation in behavior. *Neuron* **109**, 1084–1099 (2021). 10.1016/j.neuron.2021.02.002 33609484

[59] Kadobianskyi, M., Schulze, L., Schuelke, M. & Judkewitz, B. Hybrid genome assembly and annotation of *Danionella translucida*. *Sci. Data* **6**, 156 (2019). 10.1038/s41597-019-0161-z PMC6710283 31451709

[60] Kadobianskyi, M. et al. Multimodal reference brain atlas of adult *Danionella cerebrum*. Preprint at *bioRxiv* 10.64898/2026.03.09.710483 (2026).

[61] Atabay, K. D. et al. Whole-body single-cell atlas of an adult vertebrate in homeostasis and regeneration. Preprint at *bioRxiv* 10.64898/2026.02.03.703562 (2026). 42956121

[62] Zada, D., Kadobianskyi, M., Judkewitz, B. & Lovett-Barron, M. Convergent thyroid-ATPase interactions regulate collective behavior in *Danionella*. *Cell Rep.* **45**, 116730 (2025). 10.1016/j.celrep.2025.116730 PMC12934356 41417729

[63] Lee, T. J. & Briggman, K. L. Visually guided and context-dependent spatial navigation in the translucent fish *Danionella cerebrum*. *Curr. Biol.* **33**, 5467–5477.e4 (2023). 10.1016/j.cub.2023.11.030 38070503

[64] Rajan, G. et al. Evolutionary divergence of locomotion in two related vertebrate species. *Cell Rep.* **38**, 110585 (2022). 10.1016/j.celrep.2022.110585 35354040

[65] Lopes, G. et al. Bonsai: an event-based framework for processing and controlling data streams. *Front. Neuroinform.* **9**, 7 (2015). 10.3389/fninf.2015.00007 PMC4389726 25904861

[66] Lopes, G. et al. Creating and controlling visual environments using BonVision. *eLife* **10**, e65541 (2021). 10.7554/eLife.65541 PMC8104957 33880991

[67] Pereira, T. D. et al. SLEAP: A deep learning system for multi-animal pose tracking. *Nat. Methods* **19**, 486–495 (2022). 10.1038/s41592-022-01426-1 PMC9007740 35379947

[68] Pachitariu, M. et al. Suite2p: beyond 10,000 neurons with standard two-photon microscopy. Preprint at *bioRxiv* 10.1101/061507 (2016).

[69] Stringer, C. et al. Rastermap: a discovery method for neural population recordings. *Nat. Neurosci.* **28**, 201–212 (2025). 10.1038/s41593-024-01783-4 PMC11706777 39414974

[70] Pachitariu, M. & Stringer, C. Cellpose 2.0: how to train your own model. *Nat. Methods* **19**, 1634–1641 (2022). 10.1038/s41592-022-01663-4 PMC9718665 36344832

[71] Yu., J. H., Meyerhof, G. T., Milan, J., Napoli, J. L. & Lovett-Barron, M. Neuronal detection of social actions directs collective escape behavior. *Figshare*10.6084/m9.figshare.30223705 (2026). PMC13736696 42778611

