# Supplementary material for: Neuronal detection of social actions directs collective escape behaviour
Source: Nature. 2026 Sep 23;659(8139):151–9. doi: 10.1038/s41586-026-11041-1 (PMC13736696; doi:10.1038/s41586-026-11041-1)
Supplement: Supplementary file 1 [file 41586_2026_11041_MOESM2_ESM.pdf]

Corresponding author(s): Matthew Lovett-Barron

Last updated by author(s): Aug 3, 2026

Reporting Summary

Nature Portfolio wishes to improve the reproducibility of the work that we publish. This form provides structure for consistency and transparency in reporting. For further information on Nature Portfolio policies, see our Editorial Policies and the Editorial Policy Checklist.

Statistics

For all statistical analyses, confirm that the following items are present in the figure legend, table legend, main text, or Methods section.

n/a	Confirmed	
☐	☒	The exact sample size (*n*) for each experimental group/condition, given as a discrete number and unit of measurement
☐	☒	A statement on whether measurements were taken from distinct samples or whether the same sample was measured repeatedly
☐	☒	The statistical test(s) used AND whether they are one- or two-sided<br>*Only common tests should be described solely by name; describe more complex techniques in the Methods section.*
☐	☒	A description of all covariates tested
☐	☒	A description of any assumptions or corrections, such as tests of normality and adjustment for multiple comparisons
☐	☒	A full description of the statistical parameters including central tendency (e.g. means) or other basic estimates (e.g. regression coefficient) AND variation (e.g. standard deviation) or associated estimates of uncertainty (e.g. confidence intervals)
☐	☒	For null hypothesis testing, the test statistic (e.g. F, t, r) with confidence intervals, effect sizes, degrees of freedom and P value noted<br>*Give P values as exact values whenever suitable.*
☒	☐	For Bayesian analysis, information on the choice of priors and Markov chain Monte Carlo settings
☒	☐	For hierarchical and complex designs, identification of the appropriate level for tests and full reporting of outcomes
☒	☐	Estimates of effect sizes (e.g. Cohen's *d*, Pearson's *r*), indicating how they were calculated

Our web collection on statistics for biologists contains articles on many of the points above.

Software and code

Policy information about availability of computer code

Data collection	Behavioral data acquisition was automated and timestamped with prebuilt packages and custom workflows in BonsaiRx (v. 2.8.5) and BonVision (v. 0.11.0). Virtual environments were generated using Panda3D (v. 1.1015), and populated with virtual male D. cerebrum modeled in Blender (v. 3.5.1). Two-photon microscopy was acquired with a ThorLabs Bergamo II multiphoton microscope controlled by ThorImageLS 4.3, and synchronized to behavior with BonsaiRx.
Data analysis	Social LEAP-Estimates Animal Poses (SLEAP, v. 1.6.1) was used to train models for tracking the location and posture of D. cerebrum. Imaging data were motion corrected in Suite2p (v. 0.14.4), followed by cell extraction. All downstream analyses are conducted in python (v. 3.12.13), numpy (v. 2.0.2), scipy (v. 1.16.3), scikit-learn (v. 1.6.1). Code is available at https://github.com/lblabucsd/Yu_etal_2026/

For manuscripts utilizing custom algorithms or software that are central to the research but not yet described in published literature, software must be made available to editors and reviewers. We strongly encourage code deposition in a community repository (e.g. GitHub). See the Nature Portfolio guidelines for submitting code & software for further information.

Data

Policy information about availability of data

All manuscripts must include a data availability statement. This statement should provide the following information, where applicable:

- Accession codes, unique identifiers, or web links for publicly available datasets
- A description of any restrictions on data availability
- For clinical datasets or third party data, please ensure that the statement adheres to our policy

Data is available at https://doi.org/10.6084/m9.figshare.30223705

Research involving human participants, their data, or biological material

Policy information about studies with human participants or human data. See also policy information about sex, gender (identity/presentation), and sexual orientation and race, ethnicity and racism.

Reporting on sex and gender	N/A
Reporting on race, ethnicity, or other socially relevant groupings	N/A
Population characteristics	N/A
Recruitment	N/A
Ethics oversight	N/A

Note that full information on the approval of the study protocol must also be provided in the manuscript.

Field-specific reporting

Please select the one below that is the best fit for your research. If you are not sure, read the appropriate sections before making your selection.

☒ Life sciences ☐ Behavioural & social sciences ☐ Ecological, evolutionary & environmental sciences

For a reference copy of the document with all sections, see nature.com/documents/nr-reporting-summary-flat.pdf

Life sciences study design

All studies must disclose on these points even when the disclosure is negative.

Sample size	Sample size was determined based on previous studies (PMID: 39025069, PMID: 35831500).
Data exclusions	We excluded fish from imaging analysis if neurons drifted out of the field of view at any point in the recording. We excluded the first 5% of each imaging trial to avoid the influence of sound-evoked activity upon the initiation of microscope scanning.
Replication	Experiments were replicated across multiple groups of fish, with the exact N noted in each figure.
Randomization	Animals were randomly selected from the D. cerebrum colony for experiments. Group experiments used animals from the same home-tank. In all experiments with multiple stimuli, the order of stimuli were random or pseudo-random.
Blinding	Blinding was not relevant to most experiments, as all individuals received the same set of stimuli and/or a randomly-chosen choice of stimuli. In the case of comparing animal behavior in light versus dark or in different group sizes, experimenters were not blind to the lighting condition in the room or the number of fish in the tank. However, automated behavioral analyses were applied equally across all group sizes and conditions.

Reporting for specific materials, systems and methods

We require information from authors about some types of materials, experimental systems and methods used in many studies. Here, indicate whether each material, system or method listed is relevant to your study. If you are not sure if a list item applies to your research, read the appropriate section before selecting a response.

Materials & experimental systems

n/a	Involved in the study
☒	☐ Antibodies
☒	☐ Eukaryotic cell lines
☒	☐ Palaeontology and archaeology
☐	☒ Animals and other organisms
☒	☐ Clinical data
☒	☐ Dual use research of concern
☒	☐ Plants

Methods

n/a	Involved in the study
☒	☐ ChIP-seq
☒	☐ Flow cytometry
☒	☐ MRI-based neuroimaging

Animals and other research organisms

Policy information about studies involving animals; ARRIVE guidelines recommended for reporting animal research, and Sex and Gender in Research

Laboratory animals	We used wild-type or transgenic Tg(elavl3:H2B-GCaMP6s) Danionella cerebrum, of 8 weeks or older.
Wild animals	No wild animals were used.
Reporting on sex	All behavioral experiments used 8 weeks or older D. cerebrum, and both sexes are included. We did not distinguish between the sex of the animals for analyses
Field-collected samples	No field samples were collected.
Ethics oversight	The behavioral and imaging experiments with D. cerebrum were approved by the government authorities (U.S. Animal Care, and Institutional Animal Care and Use Committee (IACUC), (USDA Registration Number 93-R-0437)) and carried out in accordance with the U.S. federal and California state law to enforce the Animal Welfare Act (AWA) under the California Health and Safety Code.

Note that full information on the approval of the study protocol must also be provided in the manuscript.

Plants

Seed stocks	N/A
Novel plant genotypes	N/A
Authentication	N/A
