# Supplementary material for: Neuronal detection of social actions directs collective escape behaviour
Source: Nature. 2026 Sep 23;659(8139):151–9. doi: 10.1038/s41586-026-11041-1 (PMC13736696; doi:10.1038/s41586-026-11041-1)
Supplement: Supplementary file 2 [file 41586_2026_11041_MOESM3_ESM.pdf]

nature portfolio

Peer Review File

Neuronal detection of social actions directs collective escape behavior

Corresponding Author: Dr Matthew Lovett-Barron

Any redactions in this file are there to maintain patient confidentiality, the confidentiality of unpublished data, or to remove third-party material.

This file contains all reviewer reports in order by version, followed by all author rebuttals in order by version.

Attachments originally included by the reviewers as part of their assessment can be found at the end of this file.

Version 1:

Reviewer comments:

Referee #1

(Remarks to the Author)
This manuscript addresses a central question in the study of collective behavior, namely how individuals within animal groups detect, interpret, and act upon the social actions of their neighbors in order to coordinate rapid defensive responses. The authors focus on Danionella cerebrum, a visually social fish whose behavior and neural activity can be studied with great precision. The overarching problem explored here concerns the perceptual and neural mechanisms that enable the transmission of threat information within schooling groups, particularly when individuals do not have direct access to the external stimulus that triggered the escape of others.

The study examines two core scientific questions. First, what cues allow a fish to recognize that one of its social partners is escaping? Second, how is this information represented in the brain such that it elicits an escape response in the observer? Through an combination of behavioral assays, virtual reality experiments, and large-scale two-photon calcium imaging, the authors show that collective escape in D. cerebrum is enhanced by « social » context, that visual access to social partners is crucial for the full expression of this collective response, and that virtual conspecifics, either executing escape maneuvers or disappearing abruptly, are sufficient to trigger escape behavior in isolated real fish. On the neural side, they identify a population of neurons in the optic tectum and dorsal thalamus whose activity increases when conspecific-like stimuli disappear from the visual field. The authors interpret this “social-off” response as the neural basis for detecting the escape of neighboring fish, which in turn allows rapid and socially mediated information transfer about threats.

The manuscript is generally very well written, with clear reasoning, rigorous methodology, and thoughtfully designed experiments. The combination of virtual reality, freely moving behavioral assays, and population-scale imaging allows the authors to tackle a question that is often difficult to address in collective systems, namely what specific features of conspecific movement convey social meaning to an observer.

My overall evaluation of the work is positive. The results are original and the methodological advances are significant. However, a number of points require attention. Some contextual literature should be incorporated to better frame the species-specific nature of the observed behavior. Certain baseline measurements are missing and would allow more reliable interpretation of the magnitude of the responses. Some behavioral analyses appear incomplete, particularly those involving turning behavior, and their absence complicates the interpretation of certain results. In addition, the neural experiments rely on simplified motion stimuli (“social dots”), yet the behavioral responses to such stimuli have not been validated experimentally. This point is particularly important for substantiating the claim that these dots carry genuine “social” meaning. Finally, the discussion would benefit from a more nuanced and explicit treatment of the distinction between biological motion processing and proper social perception.

I now expand on these issues in greater detail, addressing each point to clarify how the manuscript could be further strengthened.

1. Related work

Because the manuscript examines how fish detect and respond to the escape actions of their neighbors, it is important to integrate recent work addressing how stress affects social interactions and collective organization in fish. In particular, the study by Lin et al. (2025), which demonstrates the emergence of stress-induced critical states in fish schools, is directly relevant. This work shows that species may differ markedly in their collective response to stress. Some tighten their group structure like H. rhodostomus, whereas others display expansions reminiscent of the “flash expansions” observed in D. cerebrum. Incorporating this reference would help clarify that the strong scattering response reported in this manuscript is not a generic feature of schooling fish, but rather a species-specific strategy shaped by ecological and evolutionary pressures. Including this contextual information in both the introduction and discussion would provide readers with a clearer comparative framework and underscore the ecological significance of the authors’ findings.

2. Requests for additional analyses, experiments, and clarifications

A first point that merits clarification concerns the baseline measurements of swimming speed and displacement prior to stimulus onset. Because Figures 1D–E and 1I–J report changes in speed and travel distance, it is essential to display the average swimming speed and distance traveled over a one-second period immediately preceding the presentation of the stimulus. These values should be shown for fish swimming alone and for groups of four, and under both the mechanical tap and the visual loom conditions, but without any stimulus being delivered. These baselines would make it possible to ascertain whether group effects manifest only during escape or also during routine swimming, and would provide the appropriate reference frame for evaluating the magnitude of the escape response.

A second issue arises when comparing the scattering responses to the tap and the loom stimuli. The inter-fish distance increases far more strongly in response to the visual loom (150.3 ±12.9% of baseline) than to the mechanical tap (118.9 ± 4.9% of baseline), despite the fact that the speed increase is actually larger in the tap condition (31.2±5.8 mm/s vs. 23.3±3.4 mm/s). This discrepancy suggests that turning behavior and directional variability, not speed alone, may differ strongly between stimuli. In particular, loom-driven escapes may involve more abrupt or more variable changes in heading direction, thereby generating greater spatial divergence within the group even at lower speeds. To better quantify and understand this phenomenon, it would be important to compute and present the probability distribution function of turning angle changes, ideally sampled at intervals of 0.05–0.1 seconds (or even smaller intervals), for both stimuli. This analysis is standard in the study of individual and collective swimming in fish, and similar methodologies have been applied in species such as Hemigrammus rhodostomus and Danio rerio (see for instance Escobedo et al., 2020). Presenting these distributions would make it possible to understand the behavioral origin of the observed differences in spatial expansion.

Related to this, the manuscript as a whole focuses predominantly on changes in speed, yet Figures 1B and 1G clearly show that changes in direction are also a critical component of the escape response. Because the analysis of loom responses is built around the concept of angular thresholds within the visual field, it is somewhat surprising that the authors do not analyze turning behavior in relation to these thresholds. To complement the speed-based analysis, the same probability distributions of turning angles should be computed for the loom stimulus, again for each angular threshold imposed in the analysis. This would allow a full understanding of how both speed and direction contribute to the observed behavioral responses.

Another point that requires additional experimentation concerns the use of 3D virtual models of D. cerebrum in the freely swimming VR experiments, in contrast with the simplified dots used in the neural imaging assays. The authors interpret the neural responses to the “social dots” as evidence that the brain encodes conspecific actions such as escape and routine swimming. However, it is not yet established that these dots are in fact perceived as social stimuli. Because the behavioral experiments demonstrate that fish respond robustly to photorealistic 3D conspecific models, it would be extremely valuable to conduct parallel experiments in which real fish are exposed to the same dots used in the imaging assays, displaying either typical swimming or escape kinematics. Comparing the responses to 3D models as those reportted in Figure 3 and to the simplified dots in the same condition would allow the authors to assess whether the dots are truly sufficient to elicit social responses, or whether they provoke only generic visual reactions to moving objects. Such experiments would be essential for validating the interpretation that the “social-off” neural response relates to conspecific perception rather than to general motion salience or disappearance of any biologically moving object.

3. Strengthening the discussion and interpretation

A central interpretative challenge in this manuscript lies in determining whether the neural responses attributed to social perception genuinely reflect conspecific recognition, or whether they could instead reflect a more general sensitivity to biological motion or to the sudden disappearance of salient objects. The current terminology, which refers to the stimuli as “social dots,” may implicitly suggest a degree of social specificity that has not yet been completely demonstrated. Clarifying this point in the discussion, in light of additional experiments in which real fish are exposed to the same dots used in the imaging assays and displaying either typical swimming or escape kinematics, would help establish which features of these stimuli possess genuine social relevance.
It would also be useful to clarify that the neural selectivity observed in the tectum and thalamus may reflect enhanced sensitivity to biologically plausible motion patterns, which does not necessarily imply that these stimuli are interpreted as social entities. A more thorough treatment of this conceptual distinction would greatly strengthen the manuscript, making its claims both more precise and more compelling.

Cited references

Lin, G. et al. Experimental evidence of stress-induced critical state in schooling fish. PRX Life, 3, 033018 (2025).
Escobedo et al. A data-driven method for reconstructing and modelling social interactions in animal groups. Phil. Trans. R. Soc. B., 375, 20190380 (2020).

(Remarks on code availability)
I have been able to run the code used to generate the figures

Referee #2

(Remarks to the Author)
In the manuscript, Yu and colleagues first demonstrate that individual D. cerebrum escape from threatening mechanical and visual stimuli more slowly than animals in groups of four or more. This result is convincing and suggests that signals among the fish in a group enhance escape responses. They further show that the enhancement of escape behaviors is present for mechanical stimuli both in the light and the dark, although it is stronger by some measures in the light. From there, they look in more detail into the visual enhancement.

Animals that get early access to a visual loom that reaches a critical size of 7 degrees more likely escape early in a trial, and observations of early reactors spur earlier escapes in animals that hit the 7 degree threshold later, suggesting that threat information is relayed for the early-perceivers to the late-perceivers. Isolating the behavior of a single animals presented with fictive conspecifics, the authors show that the escape or disappearance of conspecifics is sufficient to elicit behavioral escape responses.

They then perform head-tethered calcium imaging while presenting visual spheres moving like schooling fish, spheres moving like escaping fish, and spheres moving in a non-biological manner. Across the tectum and dorsal thalamus, they found that realistic stimuli elicited more neural responses than non-biological ones, and that simulated escaping fish elicited more responses than simulated schooling fish. They conclude that the visual observation of escaping conspecifics is a salient stimulus.

Interestingly, they found cells that were active in response to simulated escapes or disappearance of simulated conspecifics, but not to the disappearance of non-biologically-realistic spheres. Bridging the calcium imaging results to the more realistic free-swimming preparation, they found that escaping or disappearing virtual fish led to escapes in free-swimming animals.

There are several interesting observations arising in this manuscript. The first is the greater tendency of animals in schools to escape versus individual animals. The second is that both visual and acoustic escapes are impacted by the presence of conspecifics. The third is that it is specifically the escape behavior of conspecifics (or simulated conspecifics) that mediates this greater escape probability. The fourth and final is that there are neurons in especially in the tectum, but also in the dorsal thalamus and elsewhere, that are active specifically in response to perceived escape in conspecifics. Each of these conclusions is well supported, and collectively, they present a satisfying piece of progress in our understanding of this specific collective behavior. They provide a useful starting place for exploring whether animals with similar collective behaviors have similar stimulus specificity and neural activity.

There are also some points that require clarification or reconsideration, and these are captured in our specific comments, below. There is a lot to like in this study’s results and interpretations, but there are two considerations that might limit its appeal to Nature. The first is the breadth of collective behaviors that are addressed here. One could understand from the introduction that this manuscript provides major progress in our understanding of collective behavior in general, but it’s really restricted to visual escape specifically (and mechanical to a lesser degree). The second is the lack of function ascribed to the neurons with conspecific escape specific activity. They are strong candidates for being part of the sensorimotor pathway that mediates social escape, but their role is not tested experimentally. We are not asking the authors to address these limitations, which we view as beyond the scope of this study. We list them here for context as the editor considers the manuscript’s impact versus Nature’s expectations.

Specific comments:
1) The authors characterize the scattering response by quantifying the increase in inter-fish distances post stimulus. How long post scatter response does it take for fish to begin schooling together again? Neither main figure nor supplementary figures have a long enough timescale to show this. Also, besides magnitude of response and scattering amplitudes, was there any changes to reaction time (time from stimulus to onset of escape responses)? This would be easier to calculate for mechanosensation stimulus since the stimulus has a distinct onset.

2) The collective behavior is presented as a “flash expansion” and interpreted to be a purposeful spreading of the animals apart. A simpler explanation is that they go from an organized social group to a disorganized set of individuals, and that the spreading is simply entropy acting on the organized group rather than a designed dispersion. This could be addressed with a post-hoc analysis swapping the animals’ escape trajectories the positions of other animals at the start of the stimulus, and comparing the distributions resulting from these shuffled movements with actual distributions from the experiments. Regardless, the authors should make sure that their interpretations are firmly grounded and that alternatives are discussed when appropriate.

3) To us, the most striking result from Figure 2 is that collective enhancement of escape occurs in the dark. The authors

show that by some measures, this enhancement is not complete, and then proceed down a path of visual enhancement, but this feels too much like steering the results in a desired direction. What are the proposed mechanisms for non-visual enhancement of startle in groups? Lateral line? Olfactory? This non-visual enhancement should be addressed and discussed. Certainly calling the section “Visual access to social partners enables collective escape” seems like cherry-picking, since non-visual access does also.

4) Since Danionella school using their vision of social partners, one would assume the inter-fish distance before mechanosensation stimulus would be different in light vs dark. The normalization of inter-fish distance in Figure 2B, masks this effect. Could the authors please include non-normalized data in supplemental figures please?

5) The effect of having informed social partners on individual escape responses was well characterized by the authors. However, instead of categorizing groups as “early” or “late” groups by comparing each group’s average time to reacting critical angle, one obvious question is how many early informed fish are required for a late or non-informed fish to benefit from collective swimming? Is one early-informed fish sufficient to alert the entire group?

6) In virtual reality and two-photon imaging setup, for social stimuli, “the diameter and distance of spheres in the virtual environment were calibrated to match the size with live fish”. However, it is unclear whether the non-social stimuli (linear motion spheres), were also calibrated to match? Was the single linear motion sphere the same size as a single virtual fish or was it matched to the total size of 4 virtual fish, or neither? On this note, was the speed also matched to the average speed of virtual fish? This is an important control to clarify as the authors make a strong argument that the driving force was the biological motion of these dots that activate a specific subset of neurons that is responsible for collective escape. We think it likely that all of these controls were performed appropriately (with matching number, size, and general movement measurements as the biologically-inspired stimuli), but more details would be reassuring.

7) Fig 5 F and G suggest that a higher proportion of thalamic and forebrain neurons are responsive to disappearing conspecifics than tectal neurons. The greater number of responsive tectal neurons appears to be a function of the tectum’s large number of neurons in general, rather that the tectum’s specializing in detecting these specific stimuli. Unless we are misinterpreting these results, the authors should adjust their interpretation to reflect these numbers.

(Remarks on code availability)

Referee #3

(Remarks to the Author)
I co-reviewed this manuscript with one of the reviewers who provided the listed reports.

(Remarks on code availability)

Referee #4

(Remarks to the Author)
Summary of Key Results
Yu et al. investigated collective escape behavior in Danionella by comparing the response to threats in single versus grouped fish. They reported an average increase in threat response within grouped fish. Furthermore, they examined the visual representation of escaping conspecifics using 2-photon imaging in live fish exposed to virtual visual cues. They identified a neural population in the tectum and dorsal thalamus that selectively responds to stimuli mimicking social trajectories, and specifically to the offset of these stimuli.
Originality and significance
In this study, the authors set out to study the neural mechanism by which predator information is shared within a group of animals to drive collective escape. This is an important and completely open question in the field of collective behavior, as collective escape has primarily been studied in species where large-scale neural recording during the behavior is extremely difficult, if not impossible. The establishment of a behavior and imaging paradigm in which Danionella escape is driven by virtual conspecific behavior is exciting, and should lead to important new discoveries about how animals perceive and respond to social cues.
Data and Methodology
In order to provide novel insights into the mechanism of collective escape, there are several ambiguities in the behavioral and imaging data that must be resolved. While the authors show that the ability to see their neighbors escaping enhances scattering behavior, questions remain about whether this escape is driven by escape of social partners, or fast movement/disappearance of any visual object. If it is the later, the mechanism is not truly novel, so this is a key point to establish. In addition, I believe that further analysis of the existing data would allow the authors to determine whether escape is driven by the predator-like stimulus, the movement of the conspecifics, or a combination of both.
On the imaging side, the identification of conspecific escape- and disappearance-driven cells is an important finding, but it is currently not clear whether these cells specifically respond to social objects, whether they also respond to other escape-driving stimuli such as looming, and whether they are more responsive in trials where the fish actually escape. These points could be clarified with additional controls and analyses, and this would provide important information about the type of visual inputs these cells are getting, and their possible roles in the circuit.

Suggested improvements
1. Direct quantification of individual fish escape, and further analysis of individual fish trajectories in Figure 1. Currently, the authors rely on speed over time to infer escape behavior, and they average the speed of many fish, which makes it difficult to compare the latency of escape in groups vs. individual fish. The apparent longer latency of single fish could be due to a wider range of response times rather than a systematic shift in timing. The authors could look at the distribution of bout speeds in their data; if it is clearly multimodal this could allow them to draw a threshold for escape swims and analyse the timing relative to the loom. Secondly, it is important to know whether the fish are escaping away from the loom or from conspecifics, or some combination. The authors could plot the directionality of escapes relative to the position of the looming stimulus and their nearest escaping neighbor to shed light on what they are primarily escaping from.
2. Non-social controls in Figure 3 to show that the "social-ness" of the school is necessary for, or at least enhances, escape. The virtual conspecifics should be replaced with position and average speed-matched visual objects that move steadily rather than in bouts, but scatter to the same extent as the school. If the scattering of the non-social objects also drives escape, that would suggest the fish are simply responding to object motion rather than getting threat information from conspecifics.
3. Direction- and position-matched non-social visual stimuli in Figure 4 and 5. Currently, the sphere moves from the peripheral to central visual field and seems to be at a different elevation from the biologically moving stimuli. These differences in direction and position could be the reason why a different set of neurons responds. The looming stimulus should also move steadily in a similar direction and elevation before expanding.
4. More complete characterization of the functional properties of social escape/disappearance neurons in Figure 5. Add a position-matched non-social looming stimulus to ask whether the neurons are general threat detectors or specifically encoding social threat information. Do head-fixed fish respond with escapes? If so, it would be helpful to know whether these neurons are responding more in escape trials.
Clarity and Context
Visual Labels: It would be helpful to label the position of the looming and vibration sources in Figure 1 (B and G) and Extended Figure 1D, so the reader can visually confirm if the escape is directed away from the source.
Looming stimulus parameters: The expansion of the looming stimulus should be clearly described in terms of size to speed ratio or degrees per second of expansion from the fish's perspective.
Definitions: The distinction between "late informed" and "not informed" fish in Extended Figure 4H is unclear. Are "not informed" fish those that never saw a loom reach critical size? Did "late informed" fish eventually see the loom exceed 7 degrees but not by the cutoff time? A clearer definition is required.
Figure Readability: The normalized time scale in Figure 2 (H, I, J, L, N, P) is difficult to interpret. Indicating exactly when the looming starts and ends would be very helpful.
Figure Panel Order: Figure 4H and 4I are currently discussed before 4F and G; reverse the panel or text order.
References
Since they find evidence for social offset neurons in the thalamus, the authors should reference studies on the role of the thalamus in threat detection in fish (e.g. Heap et al, Neuron, 2018) as well as mammals.

(Remarks on code availability)

Referee #5

(Remarks to the Author)
I co-reviewed this manuscript with one of the reviewers who provided the listed reports.

(Remarks on code availability)

Version 2:

Reviewer comments:

Referee #1

(Remarks to the Author)
I thank the authors for their thorough and constructive revision of the manuscript. They have carefully addressed all of the requests for additional analyses, experiments, and clarifications raised in my previous report. The new behavioral analyses provide a much more comprehensive quantification of the escape response at both the individual and collective levels, considerably strengthening the interpretation of the behavioral data.

I particularly appreciate the authors' decision to perform an entirely new set of imaging experiments using highly realistic virtual Danionella cerebrum. These experiments not only address one of my main concerns regarding the use of simplified visual stimuli, but also substantially extend the original study by demonstrating that the reported neural responses generalize to biologically realistic virtual conspecifics.

I also welcome the revisions made to the Discussion. The authors now avoid the potentially misleading term "social dots" and instead describe these stimuli more appropriately as visual objects exhibiting biological motion, resulting in a more accurate and balanced interpretation of their findings.

Overall, the manuscript has been substantially improved through the extensive revisions and additional analyses performed by the authors. These additions considerably strengthen the evidence supporting the main conclusions. I am satisfied with the revised version and fully support its publication.

(Remarks on code availability)
The authors provide a well-organized code repository together with a README file explaining the structure of the notebooks. Separate notebooks are provided for data processing and for generating each of the main and supplementary figures, making the workflow easy to follow. I was able to open and inspect the notebooks without difficulty, and they appear to provide a clear and useful resource for reproducing the analyses and figures presented in the manuscript. Overall, the code is well organized and should facilitate the reproducibility of the study and its reuse by the community.

Referee #2

(Remarks to the Author)
Yu and colleagues have markedly improved their manuscript in response to our concerns and the excellent comments of two other reviewers. They have satisfied all of our requests, and we now view the manuscript as appropriate for publication. We defer to the other reviewers regarding the appropriateness of responses to their concerns.

(Remarks on code availability)

Referee #3

(Remarks to the Author)
I co-reviewed this manuscript with one of the reviewers who provided the listed reports.

(Remarks on code availability)

Referee #4

(Remarks to the Author)
The revision has addressed all my major concerns with additional experiments and analyses, and I am happy to endorse publication in Nature. I have a few minor corrections which I do not need to see before acceptance:
Minor typo line 530: “Once all components were dried, fish was placed back into a holding fish and ventilated with oxygenated water to ensure gill movement.” First fish should be “the fish” and second fish should be a container of some kind
Minor typo line 533: “The triangular chamber built from two monitors” add a was
Line 582; add more info about the source of the red acrylic sheets and properties of the graphic shader; “To avoid contaminating our imaging path with green/blue light from the visual display, we lined the LCD monitors with red acrylic sheets and red-shifted the virtual environment rendering with a graphic shader.”

(Remarks on code availability)

Referee #5

(Remarks to the Author)
I co-reviewed this manuscript with one of the reviewers who provided the listed reports.

(Remarks on code availability)

Open Access This Peer Review File is licensed under a Creative Commons Attribution 4.0 International License, which permits use, sharing, adaptation, distribution and reproduction in any medium or format, as long as you give appropriate credit to the original author(s) and the source, provide a link to the Creative Commons license, and indicate if changes were made.
In cases where reviewers are anonymous, credit should be given to 'Anonymous Referee' and the source.
The images or other third party material in this Peer Review File are included in the article's Creative Commons license, unless indicated otherwise in a credit line to the material. If material is not included in the article's Creative Commons license and your intended use is not permitted by statutory regulation or exceeds the permitted use, you will need to obtain permission directly from the copyright holder.
To view a copy of this license, visit https://creativecommons.org/licenses/by/4.0/

Summary of revision for all referees:

We thank the reviewers for their helpful comments and suggestions, and appreciate the opportunity to revise our manuscript. In this new version, we have responded to the reviewers' questions, comments, and concerns.

The main changes are summarized here:

1) Further behavioral analysis of freely-moving groups (seen in Figures 1-2, EDFs 1-4),
2) New experiments examining the behavior of freely-moving individual fish with visually realistic virtual *D. cerebrum* (seen in Figures 3,5, EDFs 5,8),
3) A new imaging dataset showing neural responses to the movement and actions of visually realistic virtual *D. cerebrum* (seen in Figure 5), and
4) New additions to the text to better contextualize our findings, discuss implications and caveats, and clarify details of our methods and interpretations.

We detail our response to each comment below, in blue.

Referees' comments:

Referee #1 (Remarks to the Author):

This manuscript addresses a central question in the study of collective behavior, namely how individuals within animal groups detect, interpret, and act upon the social actions of their neighbors in order to coordinate rapid defensive responses. The authors focus on Danionella cerebrum, a visually social fish whose behavior and neural activity can be studied with great precision. The overarching problem explored here concerns the perceptual and neural mechanisms that enable the transmission of threat information within schooling groups, particularly when individuals do not have direct access to the external stimulus that triggered the escape of others.

The study examines two core scientific questions. First, what cues allow a fish to recognize that one of its social partners is escaping? Second, how is this information represented in the brain such that it elicits an escape response in the observer? Through an combination of behavioral assays, virtual reality experiments, and large-scale two-photon calcium imaging, the authors show that collective escape in D. cerebrum is enhanced by « social » context, that visual access to social partners is crucial for the full expression of this collective response, and that virtual conspecifics, either executing escape maneuvers or disappearing abruptly, are sufficient to trigger escape behavior in isolated real fish. On the neural side, they identify a population of neurons in the optic tectum and dorsal thalamus whose activity increases when conspecific-like stimuli disappear from the visual field. The authors interpret this "social-off" response as the neural basis for detecting the escape of neighboring fish, which in turn allows rapid and socially mediated information transfer about threats.

The manuscript is generally very well written, with clear reasoning, rigorous methodology, and thoughtfully designed experiments. The combination of virtual reality, freely moving behavioral assays, and population-scale imaging allows the authors to tackle a question that is often difficult to address in collective systems, namely what specific features of conspecific movement convey social meaning to an observer.

My overall evaluation of the work is positive. The results are original and the methodological advances are significant. However, a number of points require attention. Some contextual literature should be incorporated to better frame the species-specific nature of the observed behavior. Certain baseline measurements are missing and would allow more reliable interpretation of the magnitude of the responses. Some behavioral analyses appear incomplete, particularly those involving turning behavior, and their absence complicates the interpretation of certain results. In addition, the neural experiments rely on simplified motion stimuli ("social dots"), yet the behavioral responses to such stimuli have not been validated experimentally. This point is particularly important for substantiating the claim that these dots carry genuine "social" meaning. Finally, the discussion would benefit from a more nuanced and explicit treatment of the distinction between biological motion processing and proper social perception.

We thank the reviewer for their positive assessment of our manuscript, and the suggestions to improve it. We address each point below.

I now expand on these issues in greater detail, addressing each point to clarify how the manuscript could be further strengthened.

1. Related work

Because the manuscript examines how fish detect and respond to the escape actions of their neighbors, it is important to integrate recent work addressing how stress affects social interactions and collective organization in fish. In particular, the study by Lin et al. (2025), which demonstrates the emergence of stress-induced critical states in fish schools, is directly relevant. This work shows that species may differ markedly in their collective response to stress. Some tighten their group structure like H. rhodostomus, whereas others display expansions reminiscent of the "flash expansions" observed in D. cerebrum. Incorporating this reference would help clarify that the strong scattering response reported in this manuscript is not a generic feature of schooling fish, but rather a species-specific strategy shaped by ecological and evolutionary pressures. Including this contextual information in both the introduction and discussion would provide readers with a clearer comparative framework and underscore the ecological significance of the authors' findings.

We thank the reviewer for bringing this literature to our attention. We agree this is an important comparison to make, and we now address this in line 69, where we first describe the scattering behavior:

> "While some dense animal collectives show contractions or directional escape waves in response to external threats[8,27–29,45,48], we observed that *D. cerebrum* groups scattered in response to looming stimuli (**Fig. 1h**)."

48. Lin, G. *et al.* Experimental Evidence of Stress-Induced Critical State in Schooling Fish. *PRX Life* **3**, 033018 (2025).

And in line 83, where we discuss the implications of this finding:

> "This group scattering can be referred to as a flash expansion[48,51–53], a type of collective escape strategy that may improve the fitness of group members by preventing predators from following all animals in the group at once[51,54–56]. However, we note that during escapes *D. cerebrum* are not actively avoiding each other; rather, they transiently depart from their typically coherent group structure."

2. Requests for additional analyses, experiments, and clarifications

A first point that merits clarification concerns the baseline measurements of swimming speed and displacement prior to stimulus onset. Because Figures 1D–E and 1I–J report changes in speed and travel distance, it is essential to display the average swimming speed and distance traveled over a one-second period immediately preceding the presentation of the stimulus. These values should be shown for fish swimming alone and for groups of four, and under both the mechanical tap and the visual loom conditions, but without any stimulus being delivered. These baselines would make it possible to ascertain whether group effects manifest only during escape or also during routine swimming, and would provide the appropriate reference frame for evaluating the magnitude of the escape response.

This is a very useful suggestion, and we now plot this pre-stimulus speed data in **Extended Data Fig. 1d,g,k,m** for loom, and **Extended Data Figs. 2f, 3c,e,h,j** for tap stimuli. These data show that the behavioral effects we focus on here – a group-dependent increase in speed and distance travelled – are a response to the external threat stimulus. See, for instance, **Extended Data Fig. 1l** and **m** to compare after the loom to before the loom.

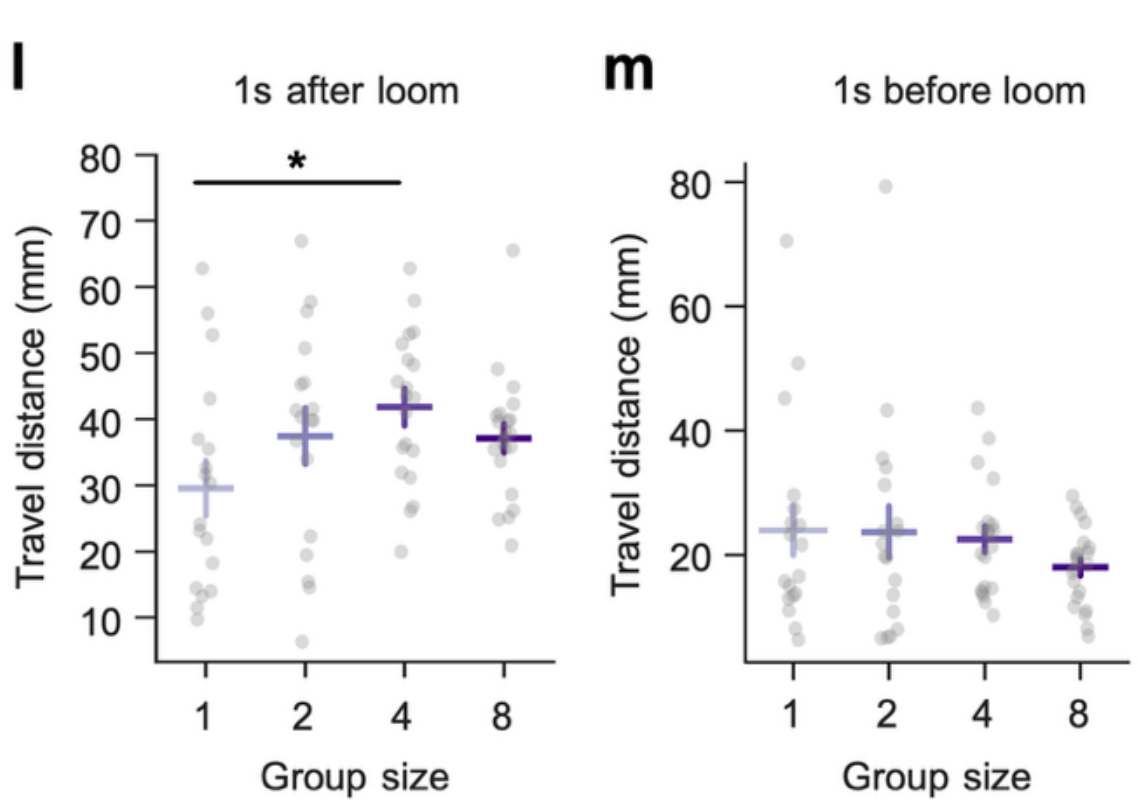

Extended Data Fig. 1. l) Travel distance (mm) 1 sec from the end of loom. N=18, 18, 20, 22 groups for single fish, group of two, group of four, and group of eight. Kruskal-Wallis: $H = 8.810$, $p = 0.0319$ followed by post hoc Dunn's test with Bonferroni correction, $p=0.0220$. **m)** Baseline travel distance (mm) 1 sec before the end of loom. N=18, 18, 20, 22 groups for single fish, group of two, group of four, and group of eight. Kruskal-Wallis: $H = 1.928$, $p = 0.587$.

A second issue arises when comparing the scattering responses to the tap and the loom stimuli. The inter-fish distance increases far more strongly in response to the visual loom (150.3 ±12.9% of baseline) than to the mechanical tap (118.9 ± 4.9% of baseline), despite the fact that the speed increase is actually larger in the tap condition (31.2±5.8 mm/s vs. 23.3±3.4 mm/s). This discrepancy suggests that turning behavior and directional variability, not speed alone, may differ strongly between stimuli. In particular, loom-driven escapes may involve more abrupt or more variable changes in heading direction, thereby generating greater spatial divergence within the group even at lower speeds. To better quantify and understand this phenomenon, it would be important to compute and present the probability distribution function of turning angle changes, ideally sampled at intervals of 0.05–0.1 seconds (or even smaller intervals), for both stimuli. This analysis is standard in the study of individual and collective swimming in fish, and similar methodologies have been applied in species such as Hemigrammus rhodostomus and Danio rerio (see for instance Escobedo et al., 2020). Presenting these distributions would make it possible to understand the behavioral origin of the observed differences in spatial expansion.

We agree with this point as well, and we now plot this data in **Fig. 1g**, **Extended Data Fig. 1n,o** for looming-evoked escape, and **Extended Data Fig. 2n-p** for tap-evoked escape, using a similar approach to Escobedo et al. (2020). To make the plots in **Figs. 1g, Extended Data Figs. 1n,o, and 2n,o** we used intervals of 250ms (to allow readers to distinguish between each time bin), whereas for the timeseries in **Fig. 1g, and Extended Data Fig. 2p**, we used 100ms intervals.

47. Escobedo, R. *et al.* A data-driven method for reconstructing and modelling social interactions in moving animal groups. *Philos. Trans. R. Soc. Lond. B Biol. Sci.* **375**, 20190380 (2020).

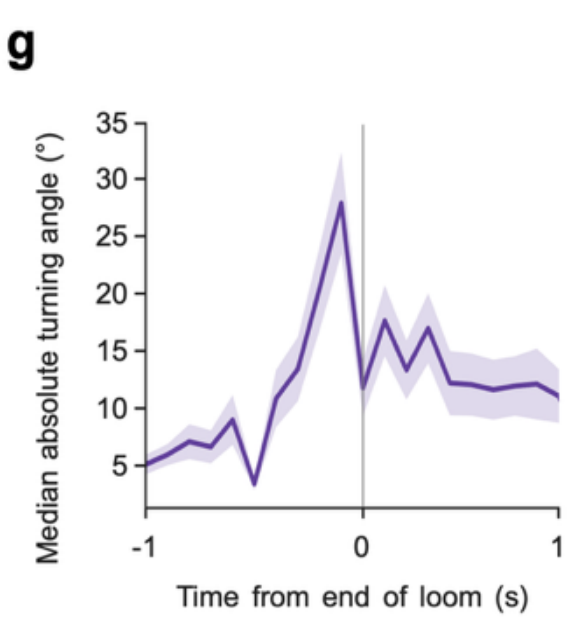

Fig 1 (*Looming stimuli*). **g)** The median turning angle (absolute change in heading direction) within 0.05 sec for all fish in groups of four. Median values in 100 ms bins from -1 to 1 sec from the end of the loom are shown in dark to light shades of purple. N=20 groups (80 fish). Note the increase of large turning angle before the end of loom.

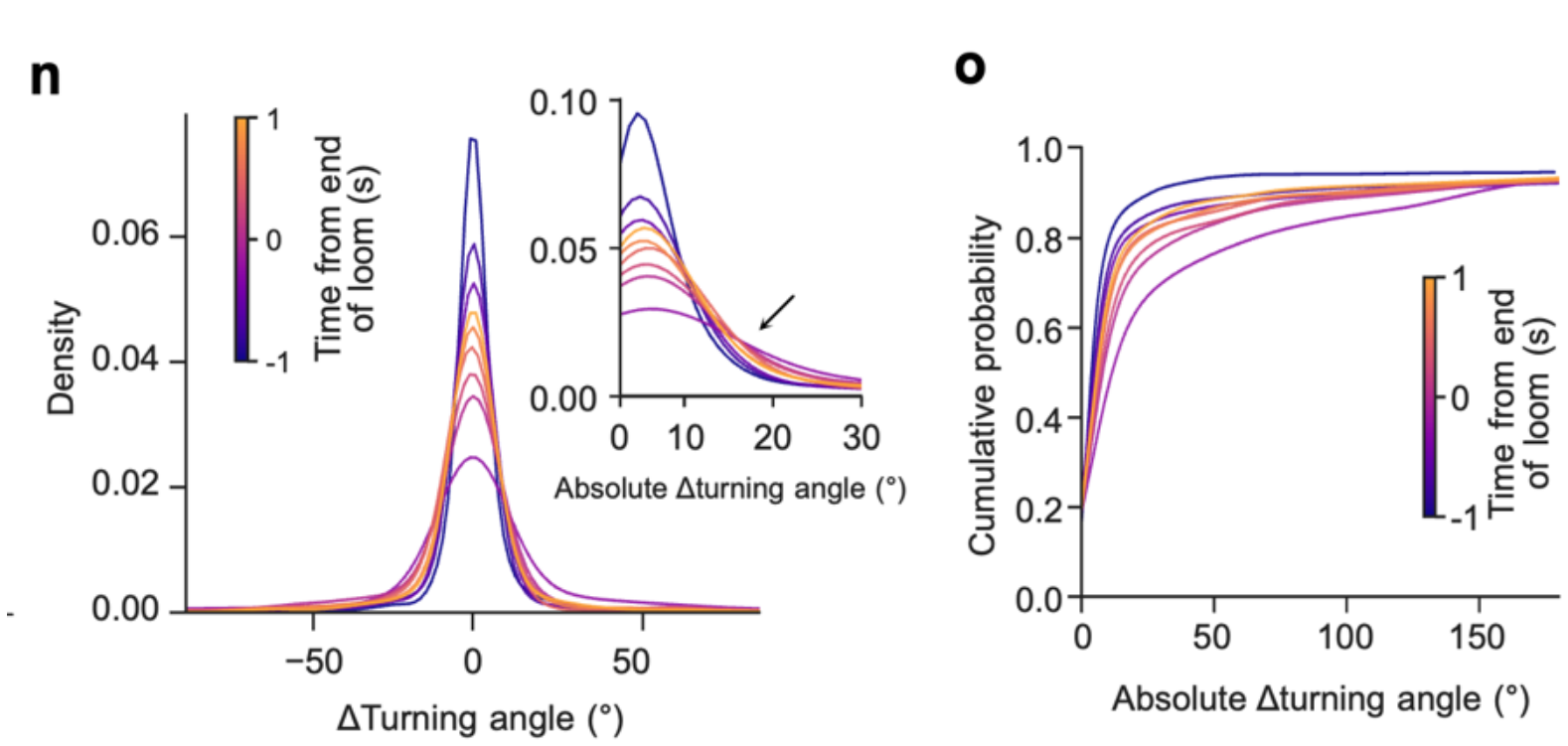

Extended Data Fig. 1. (*Looming stimuli)* **n)** Probability density function (PDF) of turning angle (change in heading direction) within 0.05 sec for all fish in groups of four. PDFs in 250 msec intervals from -1 to 1 sec from the end of the loom are shown in dark to light shades of purple. Inset shows the PDFs of absolute turning angles between 0 to 30 degrees. Note the increase of large angles 250ms before the end of loom onset marked

with arrow. N=20 groups (80 fish). **o)** Cumulative distribution function (CDF) of turning angle (absolute change in heading direction) within 0.05 sec for all fish in groups of four. CDFs in 250 msec intervals from -1 to 1 sec from the end of the loom are shown in dark to light shades of purple. N=20 groups (80 fish).

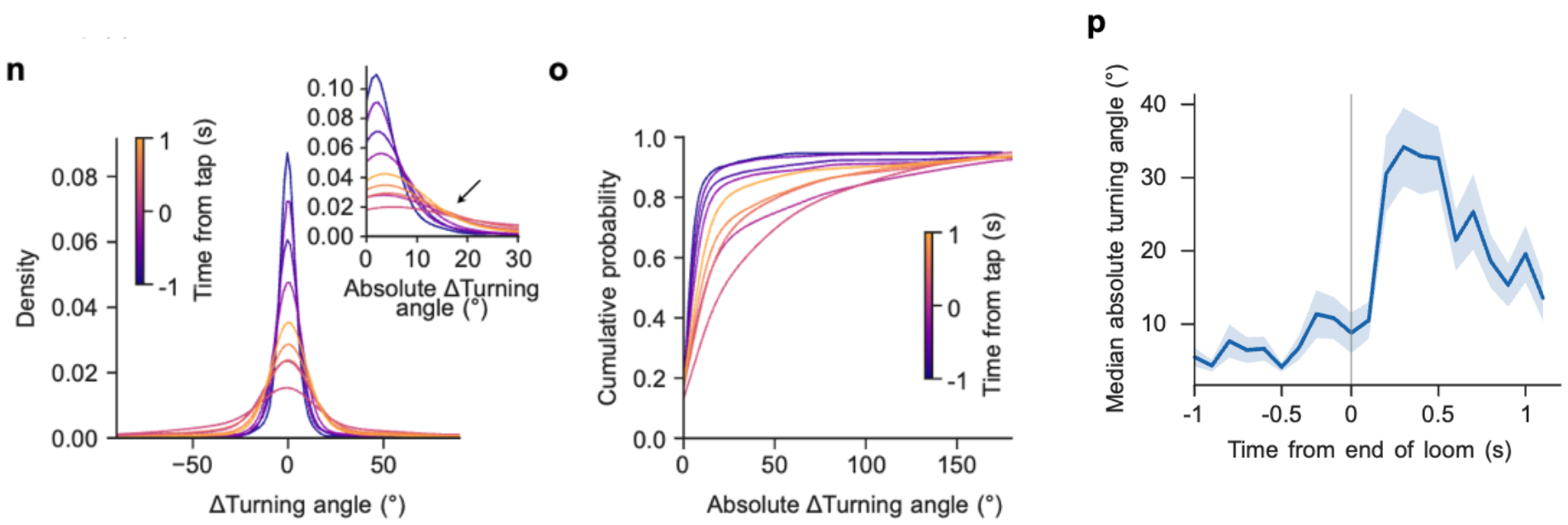

Extended Data Fig. 2. (*Tap stimuli)* **n)** Probability density function (PDF) of turning angle (absolute change in heading direction) within 0.05 sec for all fish in groups of four. PDFs in 250 msec intervals from -1 to 1 sec from the tap onset are shown in dark to light shades of blue. Inset shows the PDFs of absolute turning angles between 0 to 30 degrees. Note the increase of large angles 250ms before the tap onset marked with arrow. N=15 groups (60 fish). **o)** Cumulative distribution function (CDF) of turning angle (absolute change in heading direction) within 0.05 sec for all fish in groups of four. CDFs PDFs in 250 msec intervals from -1 to 1 sec from tap onset are shown in dark to light shades of blue. N=15 groups (60 fish). **p)** The median turning angle (absolute change in heading direction) within 0.05 sec for all fish in groups of four. Median values 100 msec intervals from -1 to 1 sec from the end of the loom are shown in dark to light shades of purple. N=15 groups (60 fish).

Related to this, the manuscript as a whole focuses predominantly on changes in speed, yet Figures 1B and 1G clearly show that changes in direction are also a critical component of the escape response. Because the analysis of loom responses is built around the concept of angular thresholds within the visual field, it is somewhat surprising that the authors do not analyze turning behavior in relation to these thresholds. To complement the speed-based analysis, the same probability distributions of turning angles should be computed for the loom stimulus, again for each angular threshold imposed in the analysis. This would allow a full understanding of how both speed and direction contribute to the observed behavioral responses.

We agree with this point as well, and we now plot this data in **Fig. 2d** for looming-evoked escape. In line 109 we write:

> "We also observed an increase in turning amplitude at these larger visual angles (**Fig. 2d**)."

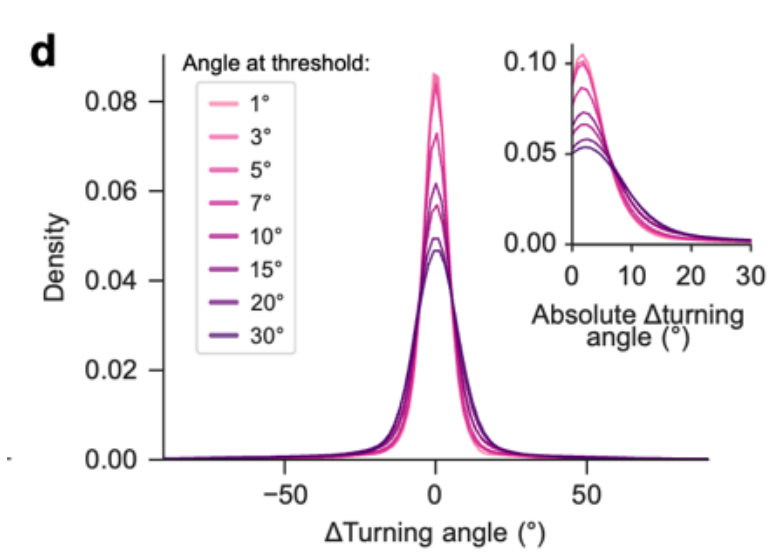

Fig. 2. d) Probability density function (PDF) of turning angle (change in heading direction) PDFs in 250 msec after crossing different visual loom angle thresholds for all fish in groups of four or larger. Inset shows the PDFs of absolute turning angles between 0 to 45 degrees. N=376, 376, 374, 333, 230, 141, 102, and 58 for 1°, 3°, 5°, 7°, 10°, 15°, 20°, and 30°, respectively.

Another point that requires additional experimentation concerns the use of 3D virtual models of D. cerebrum in the freely swimming VR experiments, in contrast with the simplified dots used in the neural imaging assays. The authors interpret the neural responses to the "social dots" as evidence that the brain encodes conspecific actions such as escape and routine swimming. However, it is not yet established that these dots are in fact perceived as social stimuli. Because the behavioral experiments demonstrate that fish respond robustly to photorealistic 3D conspecific models, it would be extremely valuable to conduct parallel experiments in which real fish are exposed to the same dots used in the imaging assays, displaying either typical swimming or escape kinematics. Comparing the responses to 3D models as those reportted in Figure 3 and to the simplified dots in the same condition would allow the authors to assess whether the dots are truly sufficient to elicit social responses, or whether they provoke only generic visual reactions to moving objects. Such experiments would be essential for validating the interpretation that the "social-off" neural response relates to conspecific perception rather than to general motion salience or disappearance of any biologically moving object.

We appreciate the reviewer bringing up this point. While behavioral engagement with simple dots has been established in the zebrafish shoaling literature (Larsch & Baier 2018; Kappel et al., 2022), we agree that we should examine the behavioral engagement of *D. cerebrum* to such stimuli. We have now examined the behavior of adult *Danionella cerebrum*'s attraction to dot stimuli moving with biological motion. Unlike zebrafish, *D. cerebrum* do not show attraction to the biological motion of dots (see **Extended Data Fig. 8a,b**), indicating that conspecific shape and/or texture is required for social attraction.

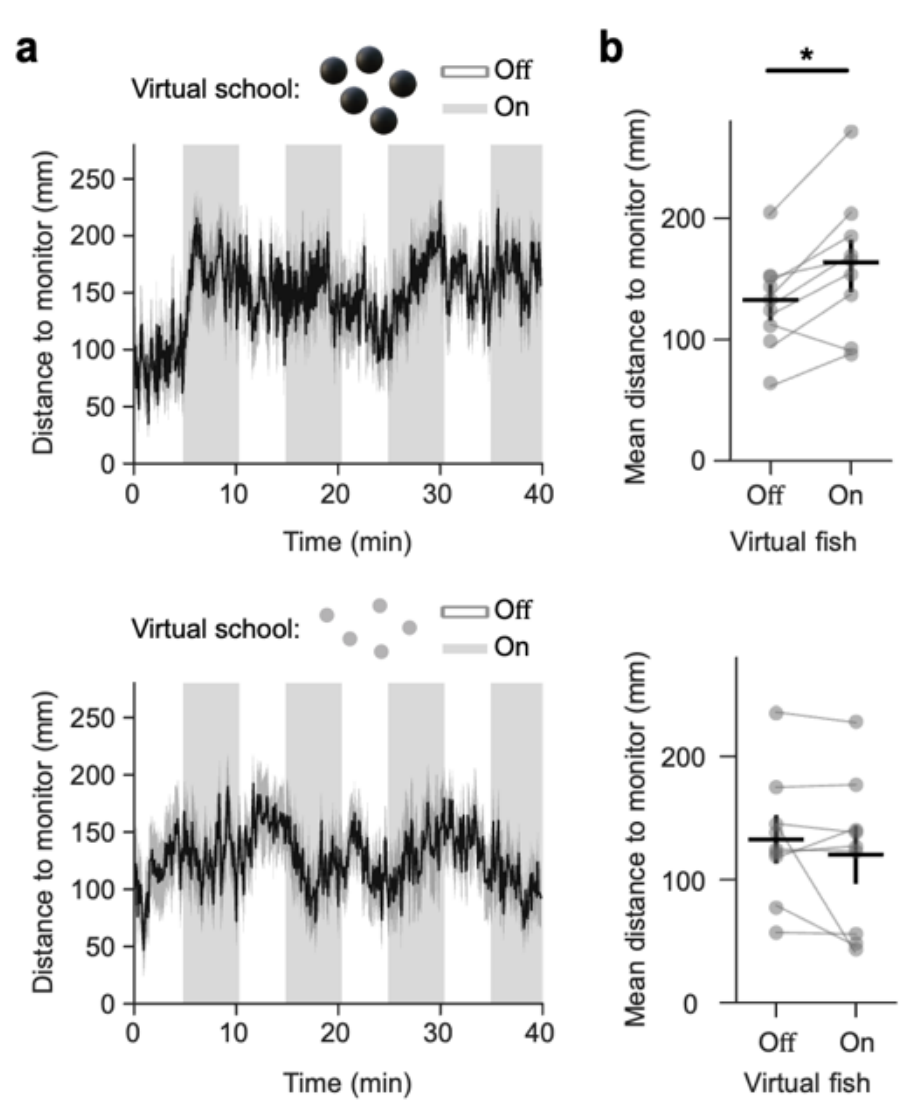

Extended Data Fig. 8. a) Distance from the monitor (mm) of the single fish over 40 min. Virtual school was rendered for four five-minute-long segments, indicated by grey shading. Images of the school are shown on top of each plot: (top) N=9 fish present with black dots, (bottom) N=9 fish present with smaller, lower contrast grey dots. **b)** Mean distance from the monitor (mm) of the single fish. Top: N=9 fish present with black dots: Wilcoxon signed-rank test = 2.0, p = 0.0117. Bottom: N=9 fish present with smaller, lower contrast grey dots: Wilcoxon signed-rank test = 14.0, p = 0.359.

Without a baseline attraction to these simple stimuli in a freely-moving condition, we cannot conduct the closed-loop experiment from **Fig. 3**, where the real fish's close proximity to the virtual school triggers the virtual escape action. We therefore conducted an entirely new set of imaging experiments, to validate and extend our

findings in dots to highly-realistic virtual *D. cerebrum* (See new **Fig. 5** and **Extended Data Fig. 8**). These virtual stimuli differ from the simple dot stimuli in 2 ways:

1) They are realistic *Danionella* mimics, with a body shape and body kinematics matched to real fish (including an undulating tail motion) – just as in our freely-moving experiments in **Figs. 3, 5k-o**, and **Extended Data Fig. 5**.

a

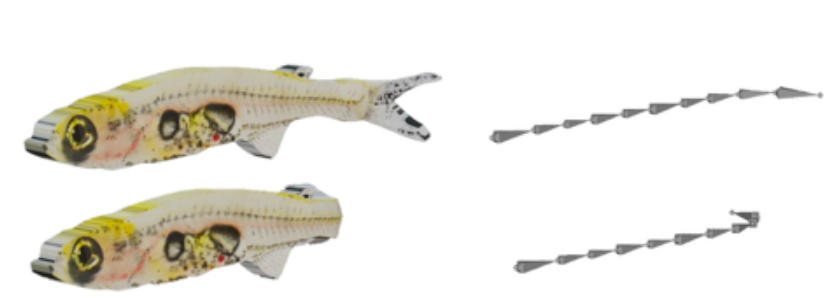

Extended Data Fig. 5. a) *D. cerebrum* model texture (left) and skeleton (right) used in the virtual reality environment. Note the bending of the tail in the model follows the curvature of the skeleton in three-dimensional space.

2) While dot stimulus motion was derived from recordings of real *Danionella*, here we programmed virtual *D. cerebrum* to move with precise parameters (new **Fig. 5a**). This ensures the virtual stimuli of different types only vary in the specifically-designed parameters. This is discussed further in response to Reviewers 2 and 3, and we describe these parameters on line 237 of the main text, and line 571 of the *Methods*:

> 237: "We programmed three virtual fish to follow a defined trajectory in visual space, moving with either biological (burst-and-glide) or linear motion; after 5 s, stimuli either escaped, vanished, or stopped moving while a looming sphere appeared (**Fig. 5a**). These stimuli allow us to examine how the visual system distinguishes between the motions and actions of virtual conspecifics, and whether these visual neurons respond to any spatially-localized visual threat."
>
> 571: "For stimulus types with biological motion, the virtual school moved with realistic burst-and-glide kinematics (rapidly accelerating and then exponentially decelerating at ~1 Hz, **Extended Data Fig. 5b**) and with animated tail movements that corresponded with the burst phase of motion. For stimulus types with linear motion, the virtual school moved with a constant velocity and without animated tail movements. The start and end position of each fish, as well as the average speed, were matched to stimuli swimming with biological motion. Movement in each trial lasted for 5 s, which corresponded to 4 burst-glide cycles, and was followed by either an escape or disappearance of the virtual fish, or the emergence of a looming stimulus. Escape trajectories spanned 45–135° away from the heading of the head-tethered fish. I.e., the virtual fish veered to the left when they were displayed on the left screen, and they veered to the right when displayed on the right screen."

We thank the reviewer for motivating these experiments, to clarify the limitations of simplified visual stimuli for the study of social behaviors, and to extend our imaging experiments to visually realistic conspecific mimics.

3. Strengthening the discussion and interpretation

A central interpretative challenge in this manuscript lies in determining whether the neural responses attributed to social perception genuinely reflect conspecific recognition, or whether they could instead reflect a more general sensitivity to biological motion or to the sudden disappearance of salient objects. The current terminology, which refers to the stimuli as "social dots," may implicitly suggest a degree of social specificity that has not yet been completely demonstrated. Clarifying this point in the discussion, in light of additional experiments in which real fish are exposed to the same dots used in the imaging assays and displaying either typical swimming or escape kinematics, would help establish which features of these stimuli possess genuine social relevance.

This is an important point, and we now use more careful language to clarify this point in the discussion. Given our findings discussed above regarding the limitations of simple visual stimuli (**Extended Data Figures 8a,b**), we no longer use the term "social dots", and now describe these as visual objects with biological motion.

Furthermore, we now show that fish will avoid virtual conspecifics that either escape or disappear after moving with biological motion, but not linear motion (see new data in **Fig. 5k-o**).

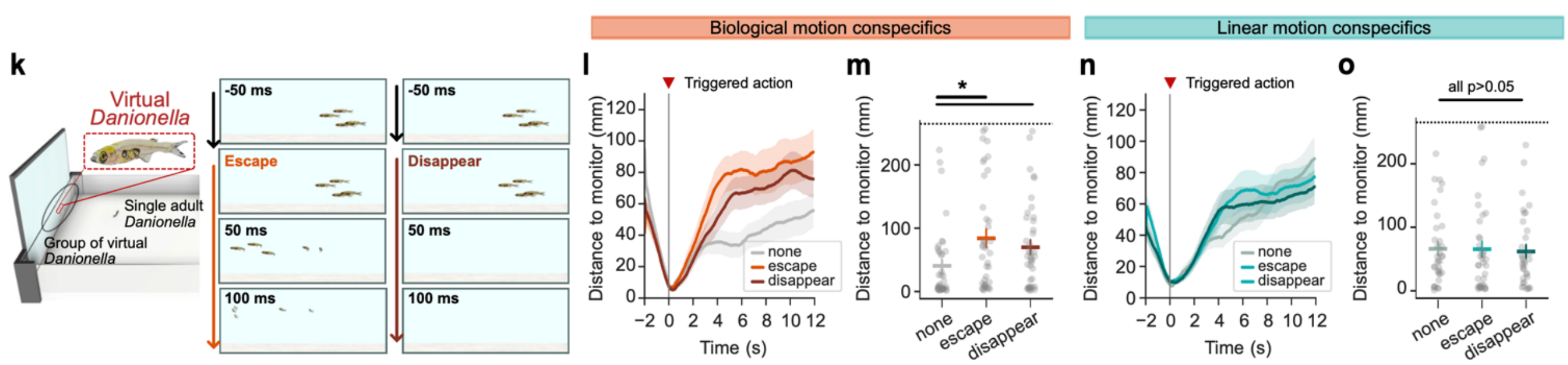

Fig. 5. k) Left: Behavior paradigm for virtual conspecifics-evoked escape: school of photorealistic *D. cerebrum* is displayed on the LCD monitor on the side of the square arena where a single *D. cerebrum* freely swims in. Right: Example frames of virtual *D. cerebrum* group on the monitor in span of 150 msec at a proximity-triggered escape and disappearance. **l)** Distance from monitor (mm) of the single fish responding to virtual *D. cerebrum* moving in biological motion. Proximity-triggered action is marked with a vertical gray line. N=39, 39, and 41 for virtual groups that continue their motion, escape or disappear, respectively. **m)** Distance from monitor (mm). Dotted grey line is the distance of the far wall (265 mm). N=39, 39, and 41 for virtual groups that continue their motion, escape or disappear, respectively. Kruskal-Wallis: H = 9.222, p = 0.0099 followed by post hoc Dunn's test: none vs. escape, p=0.0131; none vs. escape, p=0.0376. **n)** Distance from monitor (mm) of the single fish responding to virtual *D. cerebrum* moving in linear motion. Proximity-triggered action is marked with a vertical gray line. N=35, 38, 34 for virtual groups that continue their motion, escape or disappear, respectively. **o)** Distance from monitor (mm). Dotted grey line is the distance of the far wall (265 mm). N=35, 38, and 34 for virtual groups that continue their motion, escape or disappear, respectively. Kruskal-Wallis: H = 0.293, p = 0.863.

In the discussion we clarify that, while the offset response is described here for a social object, this does not preclude the possibility that other salient objects could elicit a similar response. For these fish, conspecifics are highly salient visual objects – identified in part by their biological motion patterns. We now write in line 314 of the discussion:

> "Although we found distinctions between escape behaviors driven by looming or social stimuli, the mechanisms we describe here need not be exclusive to social stimuli – they could generalize to the rapid disappearance of other visual items that have captured an animal's attention."

This could be an example whereby general-purpose neural circuits are co-opted to conduct some of the neural computations at play in species-specific social behaviors.

It would also be useful to clarify that the neural selectivity observed in the tectum and thalamus may reflect enhanced sensitivity to biologically plausible motion patterns, which does not necessarily imply that these stimuli are interpreted as social entities. A more thorough treatment of this conceptual distinction would greatly strengthen the manuscript, making its claims both more precise and more compelling.

We agree this is an important point to clarify, and now have added several statements throughout the manuscript when presenting these findings. For instance, in lines 258 and 277 of the results, we clarify the specific role of biological motion in the processes we describe in this study:

> 260: "Together, these results demonstrate that the visual system of *D. cerebrum* can identify objects that move with its species-typical burst-and-glide biological motion, and thus respond to the escape actions of those social partners."

> 277: "This data demonstrates that *D. cerebrum* will rapidly disengage from conspecifics that escape or vanish, but only if those conspecifics move with biologically realistic burst-and-glide motion."

We expand upon this topic in line 303 of the discussion:

> "Here we classify "social" stimuli as those that move with biological motion. However, biological motion alone may not always be sufficient to classify objects as being a social agent. Animals may use a combination of many sensory cues to identify conspecifics, including mechanosensory[70], auditory[41,49], or chemosensory[71] information. Integration of multisensory cues likely contributes to many collective behaviors, including escape and predator avoidance."

Referee #1 (Remarks on code availability):

I have been able to run the code used to generate the figures

We thank the reviewer for testing this. Our data and code are now updated to include the new experiments and figures in the revised manuscript.

Referee #2 (Remarks to the Author):

In the manuscript, Yu and colleagues first demonstrate that individual D. cerebrum escape from threatening mechanical and visual stimuli more slowly than animals in groups of four or more. This result is convincing and suggests that signals among the fish in a group enhance escape responses. They further show that the enhancement of escape behaviors is present for mechanical stimuli both in the light and the dark, although it is stronger by some measures in the light. From there, they look in more detail into the visual enhancement.

Animals that get early access to a visual loom that reaches a critical size of 7 degrees more likely escape early in a trial, and observations of early reactors spur earlier escapes in animals that hit the 7 degree threshold later, suggesting that threat information is relayed for the early-perceivers to the late-perceivers. Isolating the behavior of a single animals presented with fictive conspecifics, the authors show that the escape or disappearance of conspecifics is sufficient to elicit behavioral escape responses.

They then perform head-tethered calcium imaging while presenting visual spheres moving like schooling fish, spheres moving like escaping fish, and spheres moving in a non-biological manner. Across the tectum and dorsal thalamus, they found that realistic stimuli elicited more neural responses than non-biological ones, and that simulated escaping fish elicited more responses than simulated schooling fish. They conclude that the visual observation of escaping conspecifics is a salient stimulus.

Interestingly, they found cells that were active in response to simulated escapes or disappearance of simulated conspecifics, but not to the disappearance of non-biologically-realistic spheres. Bridging the calcium imaging results to the more realistic free-swimming preparation, they found that escaping or disappearing virtual fish led to escapes in free-swimming animals.

There are several interesting observations arising in this manuscript. The first is the greater tendency of animals in schools to escape versus individual animals. The second is that both visual and acoustic escapes are impacted by the presence of conspecifics. The third is that it is specifically the escape behavior of conspecifics (or simulated conspecifics) that mediates this greater escape probability. The fourth and final is that there are neurons in especially in the tectum, but also in the dorsal thalamus and elsewhere, that are active specifically in response to

perceived escape in conspecifics. Each of these conclusions is well supported, and collectively, they present a satisfying piece of progress in our understanding of this specific collective behavior. They provide a useful starting place for exploring whether animals with similar collective behaviors have similar stimulus specificity and neural activity.

There are also some points that require clarification or reconsideration, and these are captured in our specific comments, below. There is a lot to like in this study's results and interpretations, but there are two considerations that might limit its appeal to Nature. The first is the breadth of collective behaviors that are addressed here. One could understand from the introduction that this manuscript provides major progress in our understanding of collective behavior in general, but it's really restricted to visual escape specifically (and mechanical to a lesser degree). The second is the lack of function ascribed to the neurons with conspecific escape specific activity. They are strong candidates for being part of the sensorimotor pathway that mediates social escape, but their role is not tested experimentally. We are not asking the authors to address these limitations, which we view as beyond the scope of this study. We list them here for context as the editor considers the manuscript's impact versus Nature's expectations.

We thank the reviewers for their close reading and supportive comments about our manuscript. We agreed with their comments and critiques, and have addressed each point herein.

Specific comments:

1) The authors characterize the scattering response by quantifying the increase in inter-fish distances post stimulus. How long post scatter response does it take for fish to begin schooling together again? Neither main figure nor supplementary figures have a long enough timescale to show this. Also, besides magnitude of response and scattering amplitudes, was there any changes to reaction time (time from stimulus to onset of escape responses)? This would be easier to calculate for mechanosensation stimulus since the stimulus has a distinct onset.

We now show the longer-timescale behavior in **Extended Data Figs. 1q, 2l**, and **3n**. The reviewers are correct that this is easier to calculate for the mechanosensory stimulus, which has a discrete onset. For visual stimuli, we aligned behavioral measures by the time of maximum loom size. Interestingly, we found that groups would scatter and reform on a short ~10-25s timescale for both mechanosensory (**Extended Data Fig. 2l**) and visual (**Extended Data Fig. 1q**) threats. This appears to require vision, as groups in the dark do not show this rapid reassembly after mechanical startle (**Extended Data Fig. 3n**).

q

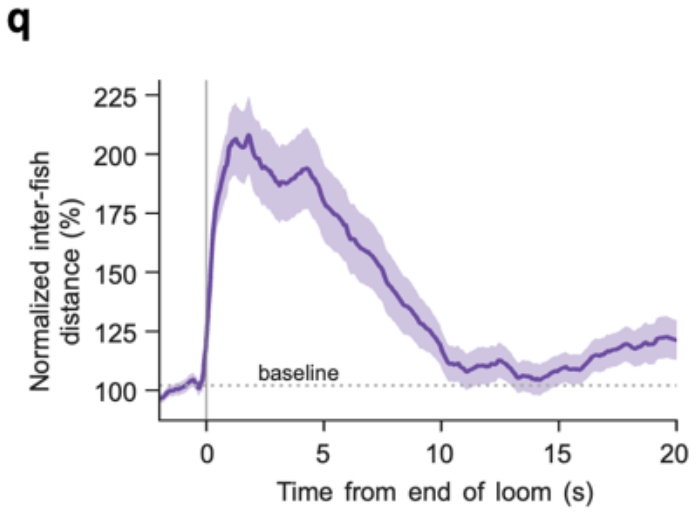

Extended Data Fig. 1. q) Normalized inter-fish distance (%) at end of the loom. Time is extended to 20 sec after the end of loom. Note the recovery of inter-fish distance to baseline at 10-15 sec post stimulus.

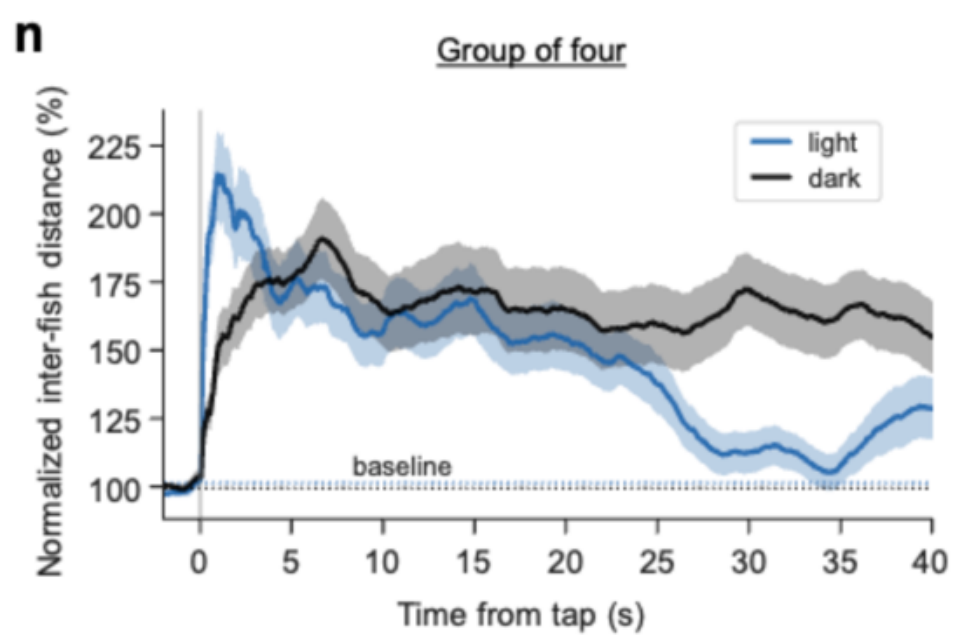

Extended Data Fig. 3. n) Normalized inter-fish distance (%) at tap onset. Time is extended to 40 sec after tap onset, Note the inter-fish distance for fish in dark condition didn't recover at the end.

2) The collective behavior is presented as a “flash expansion” and interpreted to be a purposeful spreading of the animals apart. A simpler explanation is that they go from an organized social group to a disorganized set of individuals, and that the spreading is simply entropy acting on the organized group rather than a designed dispersion. This could be addressed with a post-hoc analysis swapping the animals' escape trajectories the positions of other animals at the start of the stimulus, and comparing the distributions resulting from these shuffled movements with actual distributions from the experiments. Regardless, the authors should make sure that their interpretations are firmly grounded and that alternatives are discussed when appropriate.

We agree that our initial analyses did not distinguish between these possibilities. We now make this comparison in **Extended Data Figs. 1r** (visual) and **2m** (mechanosensory), by comparing scattering to shuffled fish identities post-loom (i.e.: a random distribution of fish).

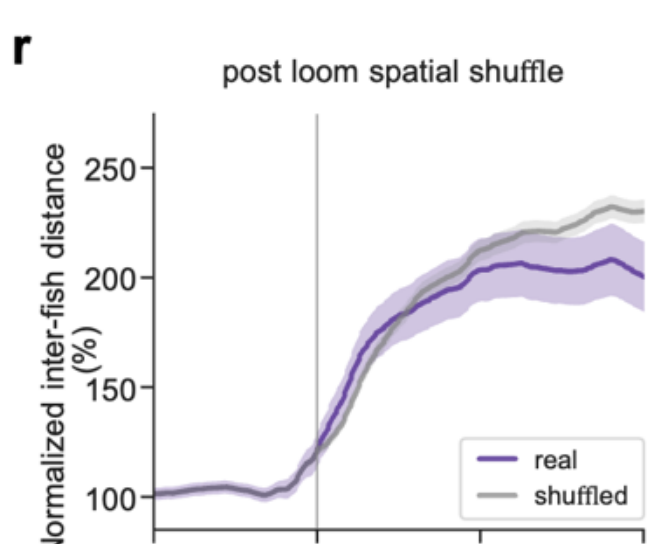

Extended Data Fig. 1. r) Normalized inter-fish distance (%) at the end of loom. Data from 5 sec before tap onset is used as the baseline. Post-loom escape trajectories are shuffled among fish to create spatially shuffled values and present in gray. N=20 groups.

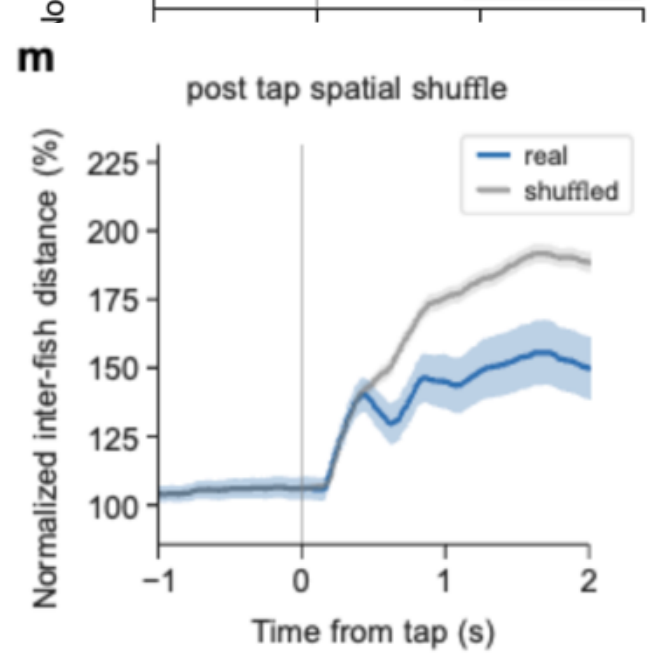

Extended Data Fig. 2. m) Normalized inter-fish distance (%) at tap onset. Data from 5 sec before tap onset is used as the baseline. Post-loom escape trajectories are shuffled among fish to create spatially shuffled values and present in gray. N=15 groups.

Indeed, we find that our observed scattering behavior is not more distributed than chance. In line 79 we now state the following:

"While collective escape to visual and mechanical stimuli are not identical (**Extended Data Fig. 2q-s**), we conclude that groups of *D. cerebrum* exhibit a stereotyped collective escape behavior to mechanosensory or visual threats by rapidly scattering. In both cases, groups only disperse briefly (**Extended Data Figs. 1q, 2l**) and no more than a random arrangement (**Extended Data Figs. 1r, 2m**). This group scattering can be referred to as a flash expansion[48,51–53], a type of collective escape strategy that may improve the fitness of group members by preventing predators from following all animals in the group at once[51,54–56]. However, we note that during escapes *D. cerebrum* are not actively avoiding each other; rather, they transiently depart from their typically coherent group structure."

3) To us, the most striking result from Figure 2 is that collective enhancement of escape occurs in the dark. The authors show that by some measures, this enhancement is not complete, and then proceed down a path of visual enhancement, but this feels too much like steering the results in a desired direction. What are the proposed mechanisms for non-visual enhancement of startle in groups? Lateral line? Olfactory? This non-visual enhancement should be addressed and discussed. Certainly calling the section "Visual access to social partners enables collective escape" seems like cherry-picking, since non-visual access does also.

This is a fair criticism of how we wrote the initial manuscript, and the escape from mechanosensory stimuli is indeed interesting. We have now changed this section to be "**Visual access to social partners <u>can</u> enhance collective escape**". We have also added additional commentary discussing non-visual, group-enhanced escape in in line 305 of the discussion:

"Animals may use a combination of many sensory cues to identify conspecifics, including mechanosensory[70], auditory[41,49], or chemosensory[71] information. Integration of multisensory cues likely contributes to many collective behaviors, including escape and predator avoidance."

However, our goal in this manuscript is to characterize how information about threats are shared through the visual systems of socially-coupled *Danionella*. While both mechanosensory and visual escape behaviors show interesting collective dynamics (scattering, for example), only the visual loom-escape assay allows us to compare the behavior of more- or less-informed individuals in the group (see **Fig. 2**). This is a more naturalistic scenario for groups being attacked by a predator from one direction. As we state in line 99 of the results:

"While the mechanical threat stimulus is delivered synchronously to all group members, the visual loom stimulus is perceived asynchronously by individual fish, depending on the proximity of each fish to the approaching visual object; the size of the looming sphere in each individual's field of view depends on their position in the arena relative to its approach (**Fig. 2a, Extended Data Fig. 4a**).."

To make this focus clearer to the reader, we now only show looming-evoked escape data in the main figures (i.e., **Figs. 1** and **2**), and move all the mechanosensory-evoked escapes to **Extended Data Figs. 2** and **3**. While we have added new analyses of the mechanosensory-driven escape behavior in the revision, this arrangement ensures that the results of these tap-evoked escape experiments are reported in detail, but that the primary manuscript figures and the bulk of the text now focus on the visually-evoked behaviors.

4) Since Danionella school using their vision of social partners, one would assume the inter-fish distance before mechanosensation stimulus would be different in light vs dark. The normalization of inter-fish distance in Figure 2B, masks this effect. Could the authors please include non-normalized data in supplemental figures please?

Yes, we show this now in **Extended Data Fig. 3m**. Fish in the light are slightly closer together in the light than in the dark. This minor change may be due to the ability for *D. cerebrum* to use non-visual cues (or very dim light) to group together. Though, as we have demonstrated in prior work (Zada et al., *Current Biology* 2024), *D. cerebrum* are unable to align into polarized schools in the dark.

However, the effect of vision on grouping is more apparent in **Extended Data Fig. 3n**. Here we show that fish in the light can reform their groups after only ~ 25s post-tap, whereas fish in the dark cannot achieve this and maintain their greater distance

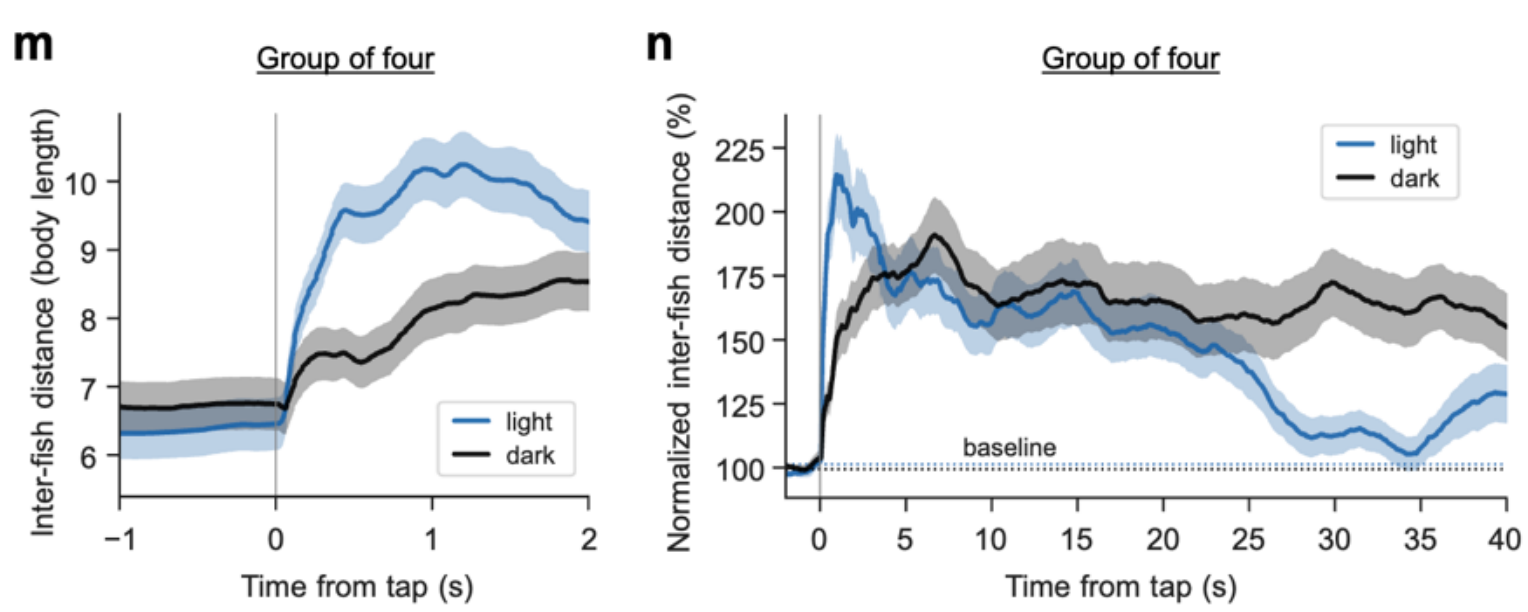

Extended Data Fig. 3. m) Inter-fish distance (body length) at tap onset. N=20 and 18 groups in light and dark conditions. **n)** Normalized inter-fish distance (%) at tap onset. Time is extended to 40 sec after tap onset, Note the inter-fish distance for fish in dark condition didn't recover at the end.

5) The effect of having informed social partners on individual escape responses was well characterized by the authors. However, instead of categorizing groups as "early" or "late" groups by comparing each group's average time to reacting critical angle, one obvious question is how many early informed fish are required for a late or non-informed fish to benefit from collective swimming? Is one early-informed fish sufficient to alert the entire group?

We thank the reviewers for this suggestion. We now plot this in **Fig. 2n,o** and **Extended Data Fig. 4k**, and do indeed find that observer escape speed does increase with the number of informed group members. In line 130 of the results, we now state:

> "Furthermore, the speed of late- or not-informed observers increases with greater number of early-informed fish in their group (**Fig. 2n,o, Extended Data**

Fig. 4k). These results indicate that individuals with limited direct perception of a looming threat stimulus can nonetheless execute a timely escape when their social partners are informed of the threat."

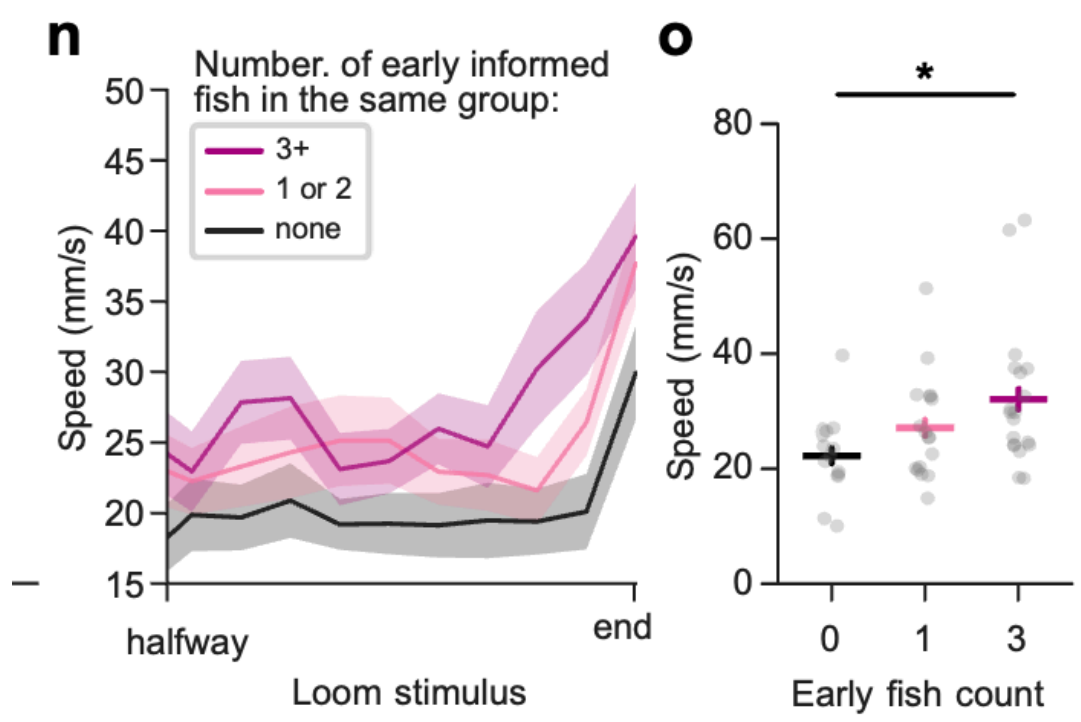

Fig. 2. n) Speed (mm/s) during loom stimulus of the late and not informed fish. N=13, 17 and 19 groups for groups with none, one to two, and more than three early informed individuals. **o)** Mean speed (mm/s) of the last 20% of loom stimulus. N=13, 17 and 19 groups for groups with none, one to two, and more than three early informed individuals. Kruskal-Wallis: H = 6.505, p = 0.0386 followed by post hoc Dunn's test with Bonferroni correction (none vs. 3+, p=0.0323).

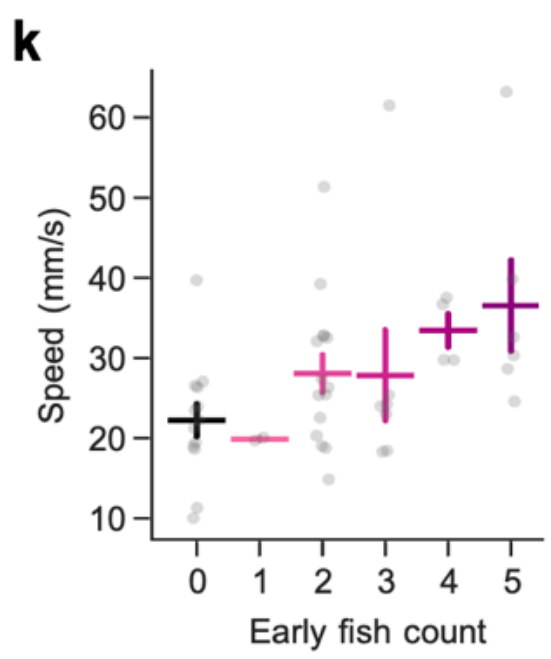

Extended Data Fig. 4. k) Speed (mm/s) of the last 20% of loom stimulus. N=13, 2, 15, 7, 4, and 6 groups for groups with 0, 1, 2, 3, 4, and 5 early informed individuals, respectively. Kruskal-Wallis: H = 15.579, p = 0.0292 followed by post hoc Dunn's test with Bonferroni correction.

6) In virtual reality and two-photon imaging setup, for social stimuli, "the diameter and distance of spheres in the virtual environment were calibrated to match the size with live fish". However, it is unclear whether the non-social stimuli (linear motion spheres), were also calibrated to match? Was the single linear motion sphere the same size as a single virtual fish or was it matched to the total size of 4 virtual fish, or neither? On this note, was the speed also matched to the average speed of virtual fish? This is an important control to clarify as the authors make a strong argument that the driving force was the biological motion of these dots that activate a specific subset of neurons that is responsible for collective escape. We think it likely that all of these controls were performed appropriately (with matching number, size, and general movement measurements as the biologically-inspired stimuli), but more details would be reassuring.

We thank the reviewers for raising this issue. We now clarify the details of the dot stimuli (**Fig. 4** and **Extended Data Figs. 6**) further in the *Methods* (line 551):

> "For nonsocial stimuli, a dark sphere was programmed to appear in the front of fish in the virtual environment. After 1 s, it either moves horizontally to the peripheral (linear motion stimulus) or move toward the virtual camera (thus, expanding in size; loom stimulus) over 5 s **(Extended Data Fig. 6b)**. For the social stimuli, behavioral recordings of swimming and escaping *D. cerebrum*

from both top-down and lateral view were processed to obtain realistic position changes of fish in 3-dimensional space (**Extended Data Fig. 6a**). Behavior tracking (121 and 200 Hz for the top-down and lateral side view) was resampled to the refreshing rate of monitors (60 Hz) and applied to spheres. The diameter and distance of the spheres in the virtual environment were calibrated to match their size with live fish. These two smaller dots swim from the back to front of the head-tethered fish in the VR environment over 6 s (swim stimulus) or executed a downward and outward escape at the 3 s mark and kept swimming farther (**Fig. 4d, Extended Data Fig. 6a,b**). All stimuli within a session were delivered to one visual field."

In addition, we conducted a new set of experiments motivated by this comment and the comments of other Reviewers. On line 170 we now state:

> "We displayed simplified biological motion stimuli[19,23,64] – fish-sized spherical objects whose motion in virtual space is derived from recordings of real *D. cerebrum* groups moving with their characteristic burst-and-glide kinematics (**Extended Data Fig. 6a**)."

These dot stimuli follow very similar, but not identical, trajectories. We therefore designed new virtual stimuli for further imaging experiments (**Fig. 5** and **Extended Data Fig. 8**), where virtual *D. cerebrum* move with precise parameters. This ensures the virtual stimuli of different types only vary in the specifically-designed parameters (described on line 571 of the methods):

> "For stimulus types with biological motion, the virtual school moved with realistic burst-and-glide kinematics (rapidly accelerating and then exponentially decelerating at ~1 Hz, **Extended Data Fig. 5b**) and with animated tail movements that corresponded with the burst phase of motion. For stimulus types with linear motion, the virtual school moved with a constant velocity and without animated tail movements. The start and end position of each fish, as well as the average speed, were matched to stimuli swimming with biological motion. Movement in each trial lasted for 5 s, which corresponded to 4 burst-glide cycles, and was followed by either an escape or disappearance of the virtual fish, or the emergence of a looming stimulus. Escape trajectories spanned 45–135° away from the heading of the head-tethered fish. I.e., the virtual fish veered to the left when they were displayed on the left screen, and they veered to the right when displayed on the right screen."

We introduce these stimuli in line 237 of the results section:

> "We programmed three virtual fish to follow a defined trajectory in visual space, moving with either biological (burst-and-glide) or linear motion; after 5 s, stimuli either escaped, vanished, or stopped moving while a looming sphere appeared (**Fig. 5a**). These stimuli allow us to examine how the visual system distinguishes

> between the motions and actions of virtual conspecifics, and whether these visual neurons respond to any spatially-localized visual threat."

We think this imaging results from this new stimulus set extends upon our previous findings using simplified dot stimuli, and rules out any potential confounds of using naturalistic conspecific trajectories.

7) Fig 5 F and G suggest that a higher proportion of thalamic and forebrain neurons are responsive to disappearing conspecifics than tectal neurons. The greater number of responsive tectal neurons appears to be a function of the tectum's large number of neurons in general, rather that the tectum's specializing in detecting these specific stimuli. Unless we are misinterpreting these results, the authors should adjust their interpretation to reflect these numbers.

This is certainly true – we focused our imaging on the tectum and surrounding structures (see **Fig. 4b**), and thus sampled more neurons there. We now clarify that thalamus and telencephalon also contain cells with these response properties, in new **Fig. 4h,** and **Extended Data Figs. 7g,h** and **8l,m**. In line 310 of the discussion, we now discuss this point explicitly:

> "We also found neurons for social action detection in the dorsal thalamus and telencephalon, which are associated with object detection, biological motion perception, and social behavior in zebrafish[23,24,60,73]. Interconnected tectal, thalamic, and forebrain circuits[21,23,73,75] may play an important role in identifying the species-typical movements of social partners to guide collective behavior.."

Referee #3 (Remarks to the Author):

I co-reviewed this manuscript with one of the reviewers who provided the listed reports.

Referee #4 (Remarks to the Author):

Summary of Key Results

Yu et al. investigated collective escape behavior in Danionella by comparing the response to threats in single versus grouped fish. They reported an average increase in threat response within grouped fish. Furthermore, they examined the visual representation of escaping conspecifics using 2-photon imaging in live fish exposed to virtual visual cues. They identified

a neural population in the tectum and dorsal thalamus that selectively responds to stimuli mimicking social trajectories, and specifically to the offset of these stimuli.

Originality and significance

In this study, the authors set out to study the neural mechanism by which predator information is shared within a group of animals to drive collective escape. This is an important and completely open question in the field of collective behavior, as collective escape has primarily been studied in species where large-scale neural recording during the behavior is extremely difficult, if not impossible. The establishment of a behavior and imaging paradigm in which Danionella escape is driven by virtual conspecific behavior is exciting, and should lead to important new discoveries about how animals perceive and respond to social cues.

We thank the reviewer for their positive assessment of our work.

Data and Methodology

In order to provide novel insights into the mechanism of collective escape, there are several ambiguities in the behavioral and imaging data that must be resolved. While the authors show that the ability to see their neighbors escaping enhances scattering behavior, questions remain about whether this escape is driven by escape of social partners, or fast movement/disappearance of any visual object. If it is the later, the mechanism is not truly novel, so this is a key point to establish. In addition, I believe that further analysis of the existing data would allow the authors to determine whether escape is driven by the predator-like stimulus, the movement of the conspecifics, or a combination of both.

These are useful points to consider, and we appreciate the opportunity to clarify these issues. We performed additional experiments and analyses, which highlight that the response we observe to social-offset is not a generic OFF or danger response. We also expanded on our interpretation in the discussion. We clarify the core finding in three ways:

1) We first explored the extent to which social-offset neurons responded specifically to characteristics found in conspecifics. We performed new behavioral and imaging experiments with visually realistic virtual conspecifics; we find that observers' neural activity distinguishes between virtual fish swimming with socially-relevant biological motion versus biologically-unrealistic linear motion (**Fig. 5b-j and Extended Data Fig. 8**). Additionally, we observe that fish only execute an avoidance response to observing the escape or disappearance of the virtual fish swimming with biological motion (**Fig. 5k-o**) Therefore, even when visual objects were matched in appearance and position in visual space, *D. cerebrum* show specific behavioral responses to the offset of virtual fish moving with biological motion.

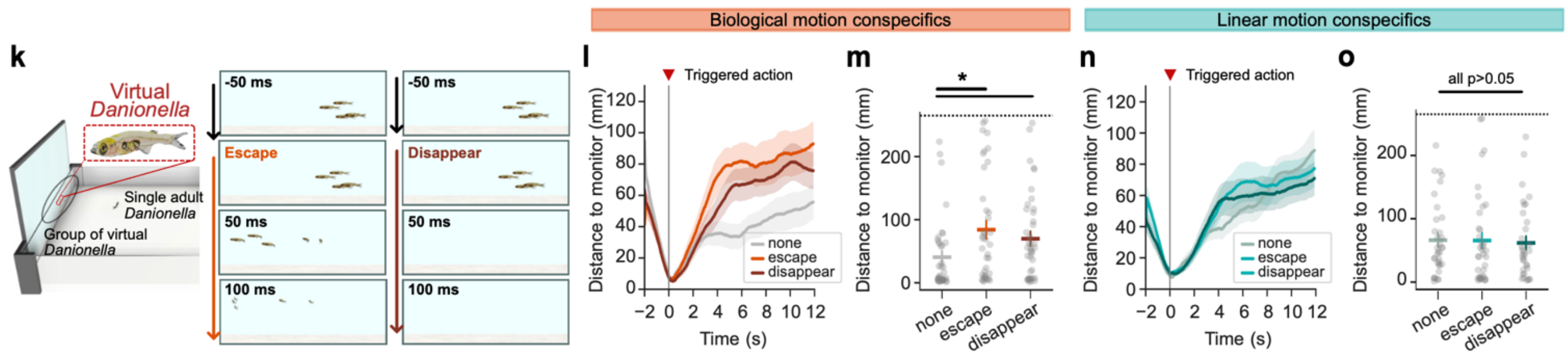

Fig. 5. k) Left: Behavior paradigm for virtual conspecifics-evoked escape: school of photorealistic *D. cerebrum* is displayed on the LCD monitor on the side of the square arena where a single *D. cerebrum* freely swims in. Right: Example frames of virtual *D. cerebrum* group on the monitor in span of 150 msec at a proximity-triggered escape and disappearance. **l)** Distance from monitor (mm) of the single fish responding to virtual *D. cerebrum* moving in biological motion. Proximity-triggered action is marked with a vertical gray line. N=39, 39, and 41 for virtual groups that continue their motion, escape or disappear, respectively. **m)** Distance from monitor (mm). Dotted grey line is the distance of the far wall (265 mm). N=39, 39, and 41 for virtual groups that continue their motion, escape or disappear, respectively. Kruskal-Wallis: H = 9.222, p = 0.0099 followed by post hoc Dunn's test: none vs. escape, p=0.0131; none vs. escape, p=0.0376. **n)** Distance from monitor (mm) of the single fish responding to virtual *D. cerebrum* moving in linear motion. Proximity-triggered action is marked with a vertical gray line. N=35, 38, 34 for virtual groups that continue their motion, escape or disappear, respectively. **o)** Distance from monitor (mm). Dotted grey line is the distance of the far wall (265 mm). N=35, 38, and 34 for virtual groups that continue their motion, escape or disappear, respectively. Kruskal-Wallis: H = 0.293, p = 0.863.

2) We next compared neural and behavioral responses to the escape of conspecifics and general danger stimuli. In a subset of visual stimuli, we replaced the biological motion fishes' escape or disappearance with a looming stimulus emerging from the virtual group. These two stimuli were easily distinguished by classifiers trained on recorded neural activity (**Fig. 5g,I** and **Extended Data Fig. 8h**) demonstrating that the social-offset response we observe is not a generic response to danger localized in a position in visual space. We state in line 250 of the results:

> "…the classifier effectively decoded looming from escape or disappearance after biological motion (classification accuracy = 66.7% and 66.4%, respectively; **Fig, 5g-i; Extended Data Fig. 8h**), suggesting these populations are not responding uniformly to all forms of visual danger."

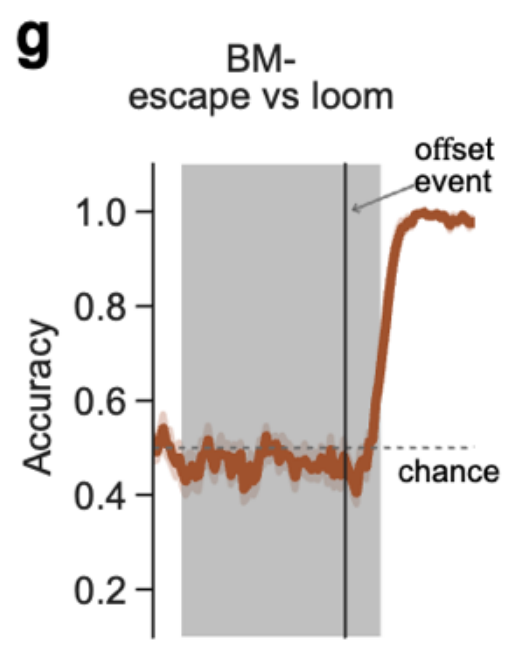

Fig. 5. g (*including context from panel e's legend*) Accuracy of support vector machine (SVM) models over time. Gray shade marks the time of stimulus presentation. Horizontal dashed line is the chance level, calculated from mean accuracy score of models trained with bootstrapped data. N=15 (15 iterations of bootstrapped each). Models are trained to classify disappearance vs. looming stimulus

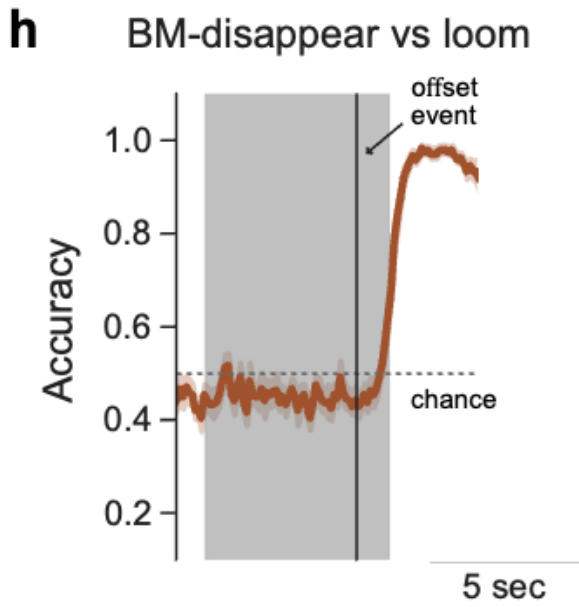

Extended Data Fig. 8. h) Accuracy of support vector machine models over time. Gray shade marks the time of stimulus presentation. Horizontal dashed line is the chance level, calculated from mean accuracy score of models trained with bootstrapped data. N=15 (15 iterations of bootstrapped each). Models are trained to classify disappear vs. loom.

In addition, we have further analyzed our data to show that looming-triggered escape is distinct from socially-inferred escape – especially in terms of speed. Specifically, when responding directly to threat, fish respond quicker than when inferring threat from conspecifics. This is important, as negative feedback is critical for suppressing continuous spread of (and potentially false) information. In line 150 of the results, we discuss this finding:

> "This escape behavior was comparable in magnitude, but delayed in onset, to escape from direct perception of a threat; when we replaced the conspecific escape action with a visual looming stimulus, observers escaped at a faster rate (see *Methods*; **Extended Data Fig. 5d,e**). This finding is in agreement with prior work showing that socially-transmitted information decays with distance[8,52,58,59], a strategy to prevent continuous transmission of threat information[29,58]."

In addition, on line 275 of the results we write:

> "Notably, the behavioral withdrawal from the offset of virtual conspecifics differs from the rapid escape from a direct threat (i.e. a loom; see *Methods*; **Extended Data Figs. 5d, 8n,o**)."

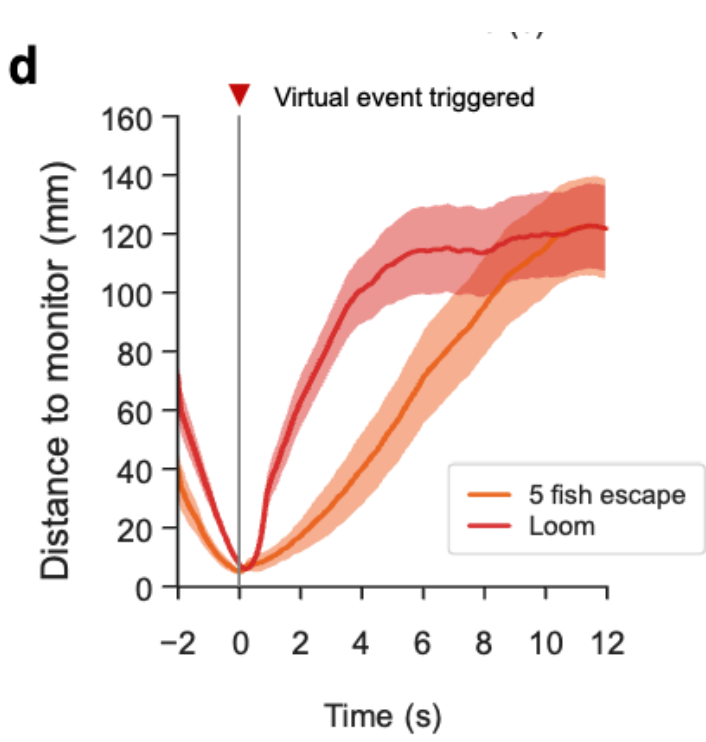

Extended Data Fig. 5. d) Distance from monitor (mm) of the single fish. Virtual escape onset/loom onset is marked with a gray line. N = 26 and 41 fish for five-virtual-fish escape and loom, respectively. Note the five-virtual-fish escape data are replotted from Fig. 3f.

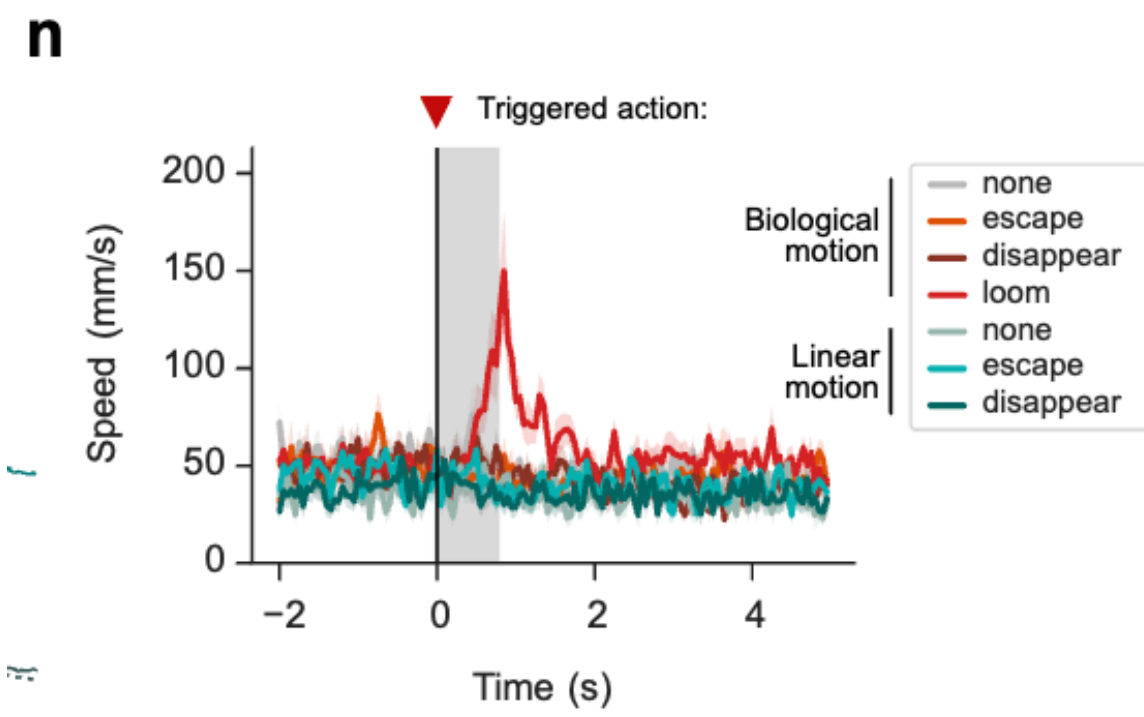

Extended Data Fig. 8. n) Speed (mm/s) of single fish exposed to various stimulus types. Vertical black line indicates proximity-triggered action of the virtual *D. cerebrum*. Grey shading indicates duration of looming stimulus. N=39, 39, 41, 42 for virtual groups that continue their motion, escape, disappear, or a looming stimulus following biological motion. N=35, 38, 34 for virtual groups that continue their motion, escape, disappear following linear motion.

3) Finally, we clarify that our observation of a social offset response need not preclude the possibility that this response could be for *any* salient object. Our point here is that, for these fish, a highly salient object is their conspecifics – identified in part by their biological motion patterns. We say in line 314 of the discussion:

> "Although we found distinctions between escape behaviors driven by looming or social stimuli, the mechanisms we describe here need not be exclusive to social stimuli – they could generalize to the rapid disappearance of other visual items that have captured an animal's attention."

As we also discussed in our response to Reviewer 1's comments, we think this could be an example whereby neural circuits capable of fundamental sensory integration functions are co-opted to conduct some of the neural computations relevant to species-specific social behaviors.

On the imaging side, the identification of conspecific escape- and disappearance-driven cells is an important finding, but it is currently not clear whether these cells specifically respond to social objects, whether they also respond to other escape-driving stimuli such as looming, and whether they are more responsive in trials where the fish actually escape. These points could be clarified with additional controls and analyses, and this would provide important information about the type of visual inputs these cells are getting, and their possible roles in the circuit.

We have now clarified this finding, with the new set of experiments described above, and further analysis of our existing data. With this we address the following points:

a) Specificity to social objects: In new imaging experiments with visually realistic virtual conspecifics, we find that observers' neural activity distinguishes between virtual fish swimming with socially-relevant biological motion versus biologically-unrealistic linear motion, even when position and trajectory is otherwise identical (**Fig. 5e,h**). This is then substantiated by our behavioral findings, that fish only execute an avoidance response to observing the escape or disappearance of the virtual fish swimming with biological motion (**Fig. 5k-o**)

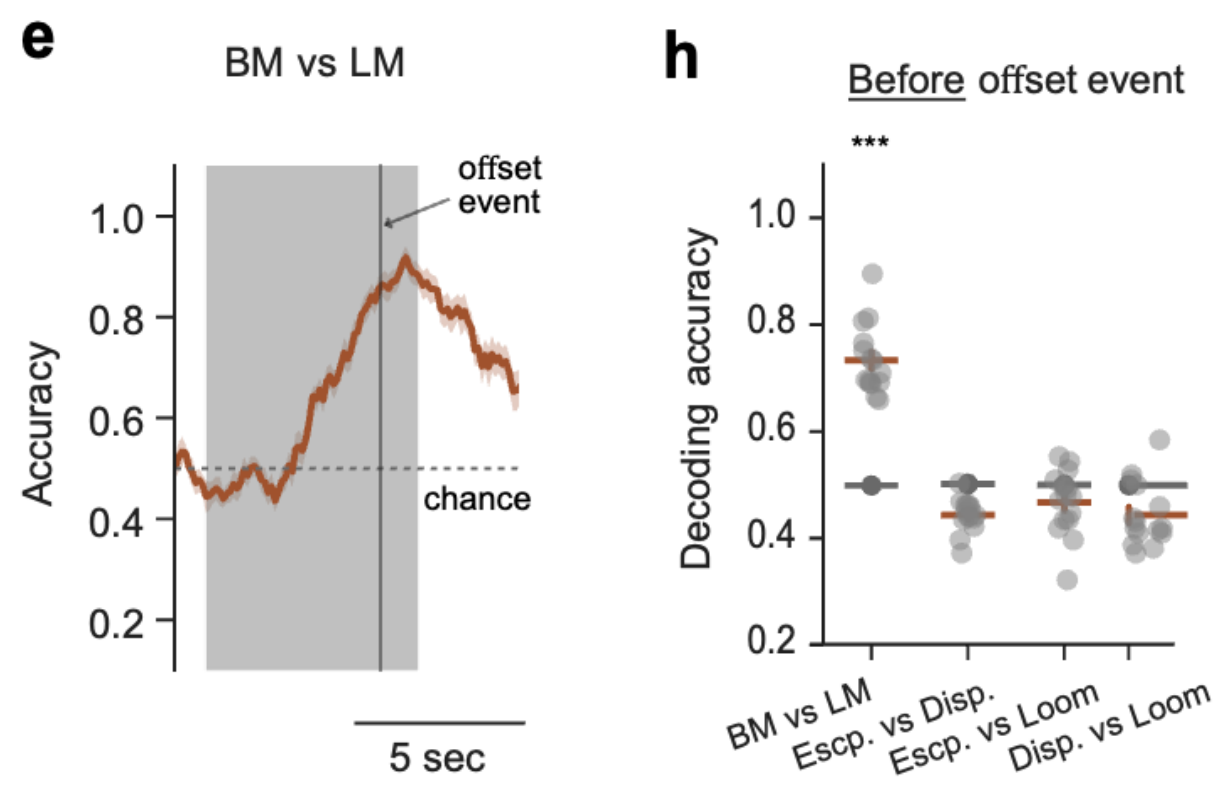

Fig. 5. e) Accuracy of support vector machine (SVM) models over time. Gray shade marks the time of stimulus presentation. Horizontal dashed line is the chance level, calculated from mean accuracy score of models trained with bootstrapped data. N=15 (15 iterations of bootstrapped each). Models are trained to classify biological motion vs. linear motion. **h)** Mean accuracy of SVM models over 2 sec before the escape or disappearance event. Each point represents the mean performance of a model trained with cells recorded in one session. N=15 (15 iterations of bootstrapped each). Gray bars represent the average performance of models trained with bootstrapped data across sessions. BM vs. LM: one-tailed T-test t = 23.449, p = 1.662×10^{-14}. Escape vs. Disappear: one-tailed Mann-Whitney U = 11.0, p = 0.999. Escape vs. Loom: one-tailed T-test t = -2.071, p = 0.976. Disappear vs. Loom: one-tailed T-test t = -3.6, p = 0.994.

b) Response to looming: As discussed in the response to the prior comment, we replaced the biological motion fishes' escape or disappearance with a small looming stimulus emerging from the virtual group. These stimuli were distinguished by classifiers trained on recorded neural activity (**Fig. 5g,l** and **Extended Data Fig. 8h**) demonstrating that the social-offset response we observe is not a generic response to danger localized in a position in visual space.

c) Modulation of visual responses by motor state. We agree that it is certainly possible that the visual responses we observe in the tectum are modulated by motor activity. Our imaging preparation does not currently allow for tethered *D. cerebrum* to execute the full range of tail motion required to escape, but we are able to monitor total rate of distal tail movement during imaging (**Extended Data Figs. 6r, 8i**). In these experiments, we observed that neurons had similar visual responses in trials with or without tail motion (**Extended Data Figs. 6s, 7i, 8j,k**). We thus conclude these are primarily visually-driven neurons, and their connections to the motor system remain to be discovered. These circuit level studies will be advanced by new tools for *Danionella* neuroscience, as we say in line 335 of the discussion:

> "Further insights into these natural behaviors will be gleaned by advancing experimental techniques for neural circuit dissection in *Danionella* and other emerging model systems capable of collective behavior[16,79]."

Suggested improvements

1. Direct quantification of individual fish escape, and further analysis of individual fish trajectories in Figure 1. Currently, the authors rely on speed over time to infer escape behavior, and they average the speed of many fish, which makes it difficult to compare the latency of escape in groups vs. individual fish. The apparent longer latency of single fish could be due to a wider range of response times rather than a systematic shift in timing. The authors could look at the distribution of bout speeds in their data; if it is clearly multimodal this could allow them to draw a threshold for escape swims and analyse the timing relative to the loom. Secondly, it is important to know whether the fish are escaping away from the loom or from conspecifics, or some combination. The authors could plot the directionality of escapes relative to the position of the looming stimulus and their nearest escaping neighbor to shed light on what they are primarily escaping from.

We have now included these suggested analyses in the revised manuscript:

1) We now quantify the distributions of speed over time for each fish (**Extended Data Figure 1e, 2g**). We quantified response rate as the time to reach a "high-speed threshold" (determined in **Extended Data Figs. 1e, 2g**) from the time since stimulus onset (tap) or offset (loom) (**Extended Data Figure 1f,2h**); see *Methods*). This data shows that in addition to the lower change in speed, single fish also had a longer reaction time.

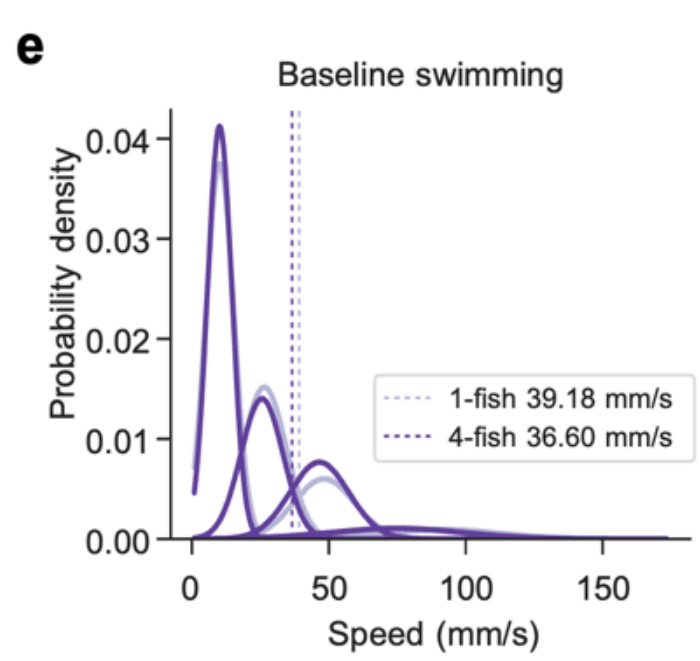

Extended Data Fig. 1. e) Probability density function (PDF) of baseline speed (mm/s) in the 30 sec before loom onset. The multimodal distributions from single fish and groups of four are fitted separately with a Gaussian mixed model (number of components determined by Bayesian Information Criterion). The boundary of the middle two components is defined as the threshold for low vs high speed states and marked with dashed lines for each group size.

The question of directionality is interesting, as we find the fish scatter away from each other. As mentioned, there is no directionality to the tap stimulus, but individual trajectories following loom presentation are shown in **Extended Data Figure 1h**. and comparison with active avoidance in **Extended Data Figure 1r**.

As we say in line 66:

> "Escaping fish also rapidly increased the magnitude of their turning, but their turns were directionally unbiased (**Fig. 1g; Extended Data Fig. 1n,o**)[47]..

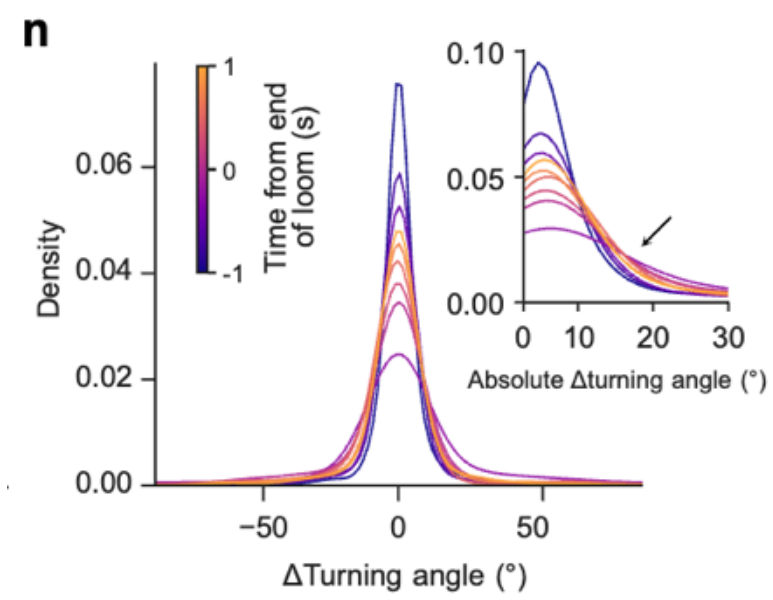

Extended Data Fig. 1. n) Probability density function (PDF) of turning angle (change in heading direction) within 0.05 sec for all fish in groups of four. PDFs in 250 msec intervals from -1 to 1 sec from the end of the loom are shown in dark to light shades of purple. Inset shows the PDFs of absolute turning angles between 0 to 30 degrees. Note the increase of large angles 250ms before the end of loom onset marked with arrow. N=20 groups (80 fish).

2. Non-social controls in Figure 3 to show that the "social-ness" of the school is necessary for, or at least enhances, escape. The virtual conspecifics should be replaced with position and average speed-matched visual objects that move steadily rather than in bouts, but scatter to the same extent as the school. If the scattering of the non-social objects also drives escape, that would suggest the fish are simply responding to object motion rather than getting threat information from conspecifics.

We thank the reviewer for this suggestion, as this forms the core of our new imaging and behavioral experiments in this revision. We conducted a new set of imaging experiments with highly-realistic virtual *D. cerebrum* (See **Fig 5** and **Extended Data Figure 8**). Dot motion in **Fig. 4** are from recordings of real *Danionella*, but in these new experiments we used virtual *D. cerebrum* with programmed movement. This ensures the virtual stimuli of different types only vary in the specifically-designed parameters: burst-and-glide motion versus linear motion, escape or disappearance (described on line 571 of the methods):

> "For stimulus types with biological motion, the virtual school moved with realistic burst-and-glide kinematics (rapidly accelerating and then exponentially decelerating at ~1 Hz, **Extended Data Fig. 5b**) and with animated tail movements that corresponded with the burst phase of motion. For stimulus types with linear motion, the virtual school moved with a constant velocity and without animated tail movements. The start and end position of each fish, as well as the average speed, were matched to stimuli swimming with biological motion. Movement in each trial lasted for 5 s, which corresponded to 4 burst-glide cycles, and was followed by either an escape or disappearance of the virtual fish, or the emergence of a looming stimulus. Escape trajectories spanned 45–135° away from the heading of the head-tethered fish. I.e., the virtual fish veered to the left when they were displayed on the left screen, and they veered to the right when displayed on the right screen."

We found that neural activity recorded in observers distinguishes between viewing virtual *D. cerebrum* moving with biological (social) or linear (non-social) motion (**Fig. 5e,h,i**), where neurons are specifically responsive to the actions of fish moving with biological motion (**Fig, 5j**).

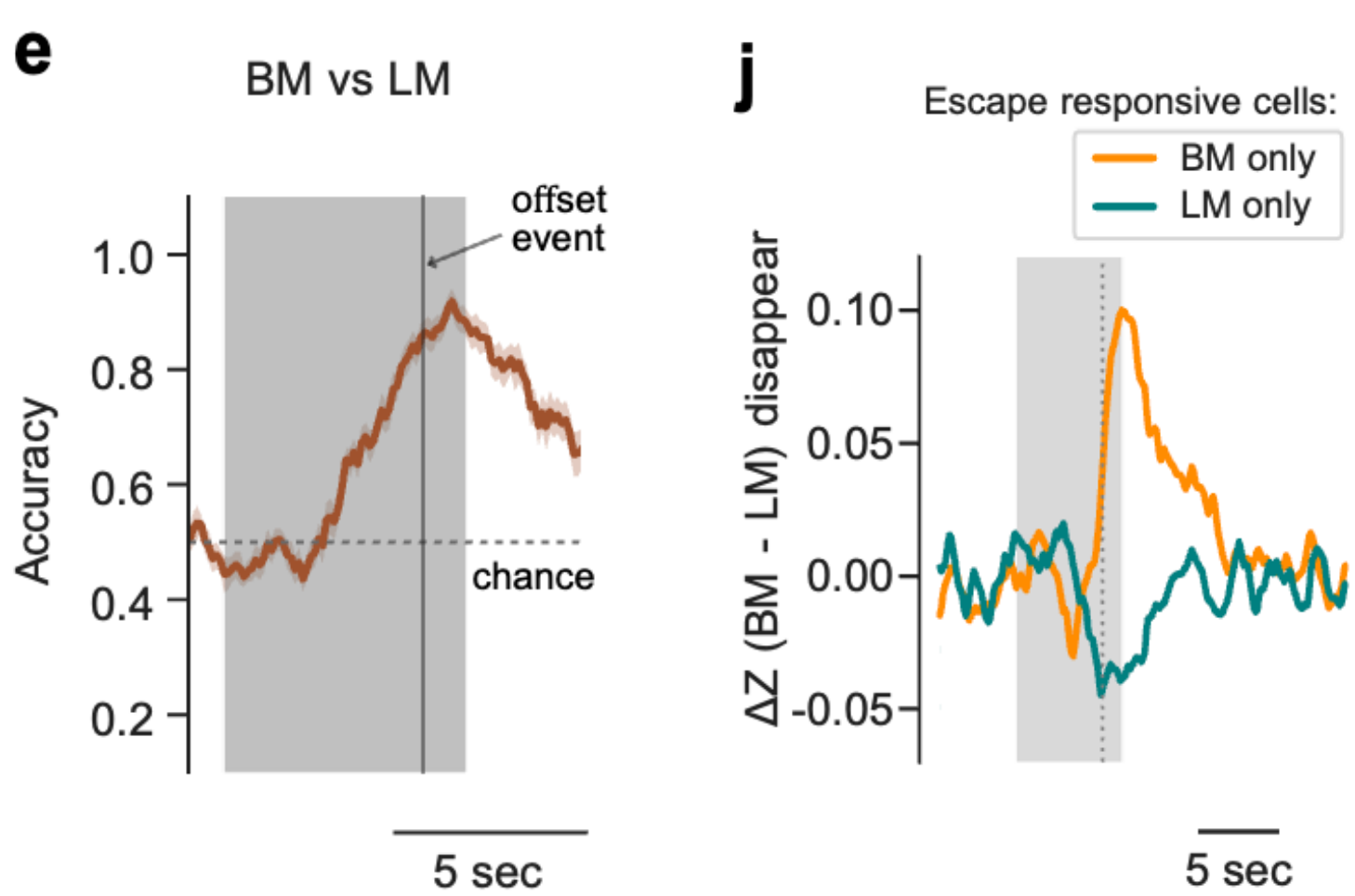

Fig. 5. e) Accuracy of support vector machine (SVM) models over time. Gray shade marks the time of stimulus presentation. Horizontal dashed line is the chance level, calculated from mean accuracy score of models trained with bootstrapped data. N=15 (15 iterations of bootstrapped each). Models are trained to classify biological motion vs. linear motion. **j)** Mean difference in Z-scored response to disappearance stimuli in escape responsive cells. that only respond to escape. N=2844, 2551 for cells that respond to escape that followed biological and linear motion only, respectively.

In addition, we conducted behavioral experiments with these VR stimuli. We observed that *D. cerebrum* only execute an avoidance response to observing the escape or disappearance of the virtual fish swimming with biological motion (**Fig. 5k-o**). On line 268, we write:

> "We examined the behavior of fish in response to the actions of virtual conspecifics swimming with biological motion, and found that either their escape or disappearance caused disengagement from the virtual group, moving further than the control (no action = 40.4 mm, escape = 84.1 mm, disappear = 69.8 mm; **Fig. 5l,m**). There was no significant difference between behavioral responses to virtual fish escaping or disappearing (p = 0.546). In contrast, when virtual conspecifics moved with non-biological linear motion, real fish did not show a behavioral distinction between no-action and their escape or disappearance (no action = 66.0 mm, escape = 65.6 mm, disappear = 61.8 mm; **Fig. 5n,o**)."

Therefore, even when visual objects were matched in terms of appearance and positions in visual space, *D. cerebrum* show specific behavioral responses to "social" objects, which we define as those moving with biological motion. We clarify our specific definition of "social" in this context on line 303 of the discussion:

> "Here we classify "social" stimuli as those that move with biological motion. However, biological motion alone may not always be sufficient to classify objects as being a social agent."

3. Direction- and position-matched non-social visual stimuli in Figure 4 and 5. Currently, the sphere moves from the peripheral to central visual field and seems to be at a different elevation from the biologically moving stimuli. These differences in direction and position

could be the reason why a different set of neurons responds. The looming stimulus should also move steadily in a similar direction and elevation before expanding.

We appreciate this point, which is why our new datasets feature virtual *D. cerebrum* that move with precise parameters, ensuring that virtual stimuli only vary in the specifically-designed parameter of interest. We describe this in line 237 of the results:

> "We programmed three virtual fish to follow a defined trajectory in visual space, moving with either biological (burst-and-glide) or linear motion; after 5 s, stimuli either escaped, vanished, or stopped moving while a looming sphere appeared (**Fig. 5a**). These stimuli allow us to examine how the visual system distinguishes between the motions and actions of virtual conspecifics, and whether these visual neurons respond to any spatially-localized visual threat."

And further describe these precise parameters in the methods (line 571), and copied in response to "suggested improvements #2", above.

4. More complete characterization of the functional properties of social escape/disappearance neurons in Figure 5. Add a position-matched non-social looming stimulus to ask whether the neurons are general threat detectors or specifically encoding social threat information. Do head fixed fish respond with escapes? If so, it would be helpful to know whether these neurons are responding more in escape trials.

As discussed above, we found that visual neurons distinguished between a position-matched loom and social escapes/disappearances (**Fig. 5g,l** and **Extended Data Fig. 8h**). In addition, we find that the observers' tail movements do not substantially impact their visual responses (**Extended Data Figs. 6s, 7i, 8j,k**).

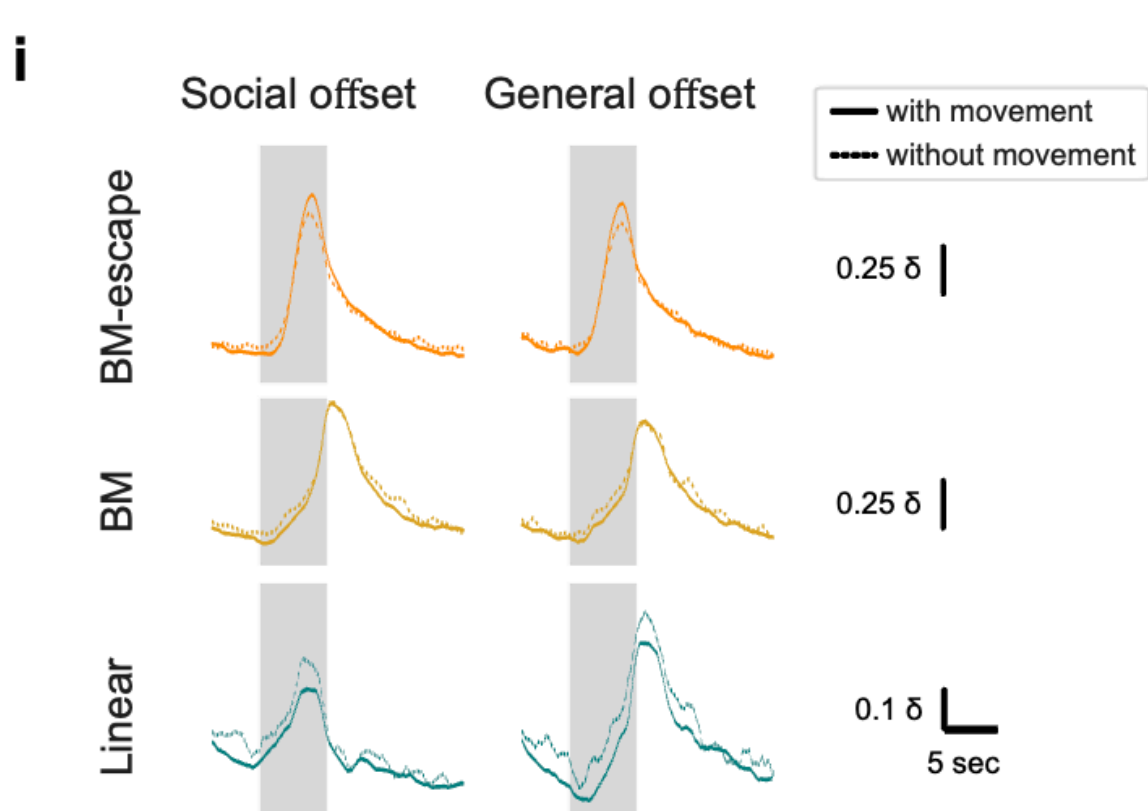

Extended Data Fig. 7. i) Mean z-scored response to different stimulus types in all fish recorded, separated by the presence of tail movement. Mean probability of tail movements during stimulus presentation is used to classify trials with motion N=15711 and 30444 "social offset" and "general offset" cells.

Clarity and Context

Visual Labels: It would be helpful to label the position of the looming and vibration sources in Figure 1 (B and G) and Extended Figure 1D, so the reader can visually confirm if the escape is directed away from the source.

We agree with this suggestion, and now show this clearly in the new **Fig. 1b** for the loom. For the vibration stimulus (new **Extended Data Figs. 2,3**), the tap vibrates the entire arena, and thus has no defined spatial focus.

Looming stimulus parameters: The expansion of the looming stimulus should be clearly described in terms of size to speed ratio or degrees per second of expansion from the fish's perspective.

On lines 413 of the *Methods*, we now state that:

> "Each experiment consisted of five 2-minute recordings, and at 1-minute mark a dark sphere of 4.3 cm would appear in one of the corners (x=±30, y=±15, z=1.5 cm) (**Extended Data Fig. 1b**) and move linearly to the center of the arena (x=0, y=0, z=1.5 cm) within 3-6 seconds. With a total virtual displacement of 33.5 cm, the loom moved at a constant speed of 5.6–11.2 cm/s, creating a length/velocity (L/V) ratio ranging from 384 to 769 ms. Both the starting position and moving duration were randomly determined. We separated each trial with a 5 minute of intervals and used groups of 1, 2, 4, and 8 fish."

Definitions: The distinction between "late informed" and "not informed" fish in Extended Figure 4H is unclear. Are "not informed" fish those that never saw a loom reach critical size? Did "late informed" fish eventually see the loom exceed 7 degrees but not by the cutoff time? A clearer definition is required.

On line 441 of the methods, we now clarify that:

> "We created Boolean arrays to identify when each fish first senses the loom at the threshold angle of 7°. These arrays were resampled to align trials of different lengths (speeds of 5.6–11.2 cm/s), and construct the cumulative probability curve of reaching loom threshold (**Fig. 2e, Extended Data Fig. 4b**). Using trials aligned from loom start to loom end, we identified the time through the trial where 50% of group members pass the angle threshold. This timepoint is used to assign individual and group information status in the subsequent analysis. For individuals, they were labeled as "early informed" if loom reaches critical angle at an earlier time than this cutoff. Otherwise, individuals were labeled as "late informed", including those that never reached critical angles and didn't have a valid reaching time (i.e.: the angle of the loom never reached 7°). The mean of the threshold-reaching time in a group was also compared to the same cutoff to assign the group as "early group" or "late group" (**Fig. 2e**). Therefore, early groups contained more early-informed individuals, and vice versa. To compare behavior across trials, we calculated the average speed in time bins 5% of the original trial length."

Figure Readability: The normalized time scale in Figure 2 (H, I, J, L, N, P) is difficult to interpret. Indicating exactly when the looming starts and ends would be very helpful.

Thank you for noting this – we now clearly note on the x-axis that this is at the midpoint to the end of the looming stimulus.

Figure Panel Order: Figure 4H and 4I are currently discussed before 4F and G; reverse the panel or text order.

Thank you for noting this – we have now corrected this ordering.

References

Since they find evidence for social offset neurons in the thalamus, the authors should reference studies on the role of the thalamus in threat detection in fish (e.g. Heap et al, Neuron, 2018) as well as mammals.

We appreciate the authors for pointing this out, and we now cite these references in an expanded section of the discussion, starting on line 308:

> "It remains unknown whether the specific "social offset" response we observe here emerges from the ON/OFF organization of presynaptic retinal ganglion cells[72], or if it is generated by local circuits in the midbrain or thalamus[20,21,73,74]. We also found neurons for social action detection in the dorsal thalamus and telencephalon, which are associated with object detection, biological motion perception, and social behavior in zebrafish[23,24,60,73]. Interconnected tectal, thalamic, and forebrain circuits[21,23,73,75] may play an important role in identifying the species-typical movements of social partners to guide collective behavior."

73. Heap, L. A. L., Vanwalleghem, G., Thompson, A. W., Favre-Bulle, I. A. & Scott, E. K. Luminance changes drive directional startle through a thalamic pathway. *Neuron* **99**, 293-301.e4 (2018).

74. Salay, L. D., Ishiko, N. & Huberman, A. D. A midline thalamic circuit determines reactions to visual threat. *Nature* **557**, 183–189 (2018).

Referee #5 (Remarks to the Author):

I co-reviewed this manuscript with one of the reviewers who provided the listed reports.

In summary, we thank all the Reviewers for their careful reading of our manuscript, and appreciate the time they took to provide thorough feedback. We have responded to the reviewers' comments in our substantially revised manuscript, and have taken their advice to improve our study. We believe our updated manuscript, figures, source data, and code, are well suited for publication.

Summary of revision:

We thank the referees for their detailed assessment of our revised manuscript, and appreciate their positive assessments. We are grateful they share our enthusiasm about these findings.

We detail our response to each Referee below, in blue.

Referee #1 (Remarks to the Author): I thank the authors for their thorough and constructive revision of the manuscript. They have carefully addressed all of the requests for additional analyses, experiments, and clarifications raised in my previous report. The new behavioral analyses provide a much more comprehensive quantification of the escape response at both the individual and collective levels, considerably strengthening the interpretation of the behavioral data.

I particularly appreciate the authors' decision to perform an entirely new set of imaging experiments using highly realistic virtual Danionella cerebrum. These experiments not only address one of my main concerns regarding the use of simplified visual stimuli, but also substantially extend the original study by demonstrating that the reported neural responses generalize to biologically realistic virtual conspecifics. I also welcome the revisions made to the Discussion. The authors now avoid the potentially misleading term "social dots" and instead describe these stimuli more appropriately as visual objects exhibiting biological motion, resulting in a more accurate and balanced interpretation of their findings.

Overall, the manuscript has been substantially improved through the extensive revisions and additional analyses performed by the authors. These additions considerably strengthen the evidence supporting the main conclusions. I am satisfied with the revised version and fully support its publication.

We thank referee #1 for their review of our revised manuscript, and are glad to see they agreed with the changes we made in this past revision.

Referee #1 (Remarks on code availability): The authors provide a well-organized code repository together with a README file explaining the structure of the notebooks. Separate notebooks are provided for data processing and for generating each of the main and supplementary figures, making the workflow easy to follow. I was able to open and inspect the notebooks without difficulty, and they appear to provide a clear and useful resource for reproducing the analyses and figures presented in the manuscript. Overall, the code is well organized and should facilitate the reproducibility of the study and its reuse by the community.

Thank you for checking the code.

Referee #2 (Remarks to the Author): Yu and colleagues have markedly improved their manuscript in response to our concerns and the excellent comments of two other reviewers. They have satisfied all of our requests, and we now view the manuscript as appropriate for

publication. We defer to the other reviewers regarding the appropriateness of responses to their concerns.

Referee #3 (Remarks to the Author): I co-reviewed this manuscript with one of the reviewers who provided the listed reports.

We thank referees #2 and #3 for the time they took to assess our work, and appreciate their positive assessment of the revised manuscript.

Referee #4 (Remarks to the Author): The revision has addressed all my major concerns with additional experiments and analyses, and I am happy to endorse publication in Nature. I have a few minor corrections which I do not need to see before acceptance: Minor typo line 530: "Once all components were dried, fish was placed back into a holding fish and ventilated with oxygenated water to ensure gill movement." First fish should be "the fish" and second fish should be a container of some kind Minor typo line 533: "The triangular chamber built from two monitors" add a was Line 582; add more info about the source of the red acrylic sheets and properties of the graphic shader; "To avoid contaminating our imaging path with green/blue light from the visual display, we lined the LCD monitors with red acrylic sheets and red-shifted the virtual environment rendering with a graphic shader."

We thank the referee for this assessment, and for their detailed read. We have now addressed these remaining points:

"Once all components were dried, the fish was placed back into a holding dish and ventilated with oxygenated water to ensure gill movement."

"To avoid contaminating our imaging path with green/blue light from the visual display, we lined the LCD monitors with red gel light filters (LILIYA, 8.5x11 inch cut to size) and red-shifted the virtual environment rendering with a graphic shader (Background color is set as Light Coral (hex code #f08080); ambient light RGB set to 1/0.5/0.5 to bias the light reflection from the seafloor and objects toward longer wavelength)."

Referee #5 (Remarks to the Author): I co-reviewed this manuscript with one of the reviewers who provided the listed reports.

We thank referees #4 and #5 for their time and effort, and appreciate their positive appraisal of our paper.

In summary, we thank all the Referees for providing useful feedback on our original submission, and taking the time to assess our revised manuscript. We are grateful they have now endorsed publication of our work.
