# Supplementary material for: Neuronal detection of social actions directs collective escape behaviour
Source: Nature. 2026 Sep 23;659(8139):151–9. doi: 10.1038/s41586-026-11041-1 (PMC13736696; doi:10.1038/s41586-026-11041-1)
Supplement: Supplementary file 3 [file 41586_2026_11041_MOESM1_ESM.docx]

**Supplementary Table 1**. Statistics and sample sizes

| **Figure** | **Group** | **Sample size** | **Statistical tests** | **P value** |
| --- | --- | --- | --- | --- |
| **1e** | single fish  four-fish group | n = 18 sessions  n = 20 sessions | two-sided T-test | t = -2.229, p = 0.0321 |
| **1f** | single fish  four-fish group | n = 18 sessions  n = 20 sessions | two-sided T-test | t = -2.754, p = 0.00917 |
| **1i** | single fish  four-fish group | n = 18 sessions  n = 20 sessions | two-sided Mann-Whitney test | U = 336.0, p = 0.000247 |
| **2b,2d** | loom threshold 1°, 3°, 5°, 7°, 10°, 15°, 20°, and 30° | n = 376, n = 376, n = 374, n = 333,  n = 230, n = 141, n = 102, n = 58 sessions | n/a | n/a |
| **2c** | loom threshold 1° (1), 3° (2), 5° (3), 7° (4), 10° (5), 15°(6), 20° (7), and 30° (8) | n = 372, 370, 371, 332, 230, 141, 102, 58 sessions | Kruskal-Wallis test | H = 78.309, p = 3.46 x 10^-14^ |
|  |  |  | post hoc Dunn’s test with Bonferroni correction | (1, 2), p = 1.00  (1, 3), p = 1.00  (1, 4), p = 0.0203  (1, 5), p = 7.90 x 10^-5^  (1, 6), p = 1.90 x 10^-5^  (1, 7), p = 2.70 x 10^-5^  (1, 8), p = 6.50 x 10^-5^  (2, 3), p = 1.00  (2, 4), p = 0.0620  (2, 5), p = 3.10 x 10^-4^  (2, 6), p = 6.60 x 10^-5^  (2, 7), p = 8.00 x 10^-5^  (2, 8), p = 1.49 x 10^-4^  (3, 4), p = 0.500  (3, 5), p = 0.00451  (3, 6), p =7.72 x 10^-4^  (3, 7), p = 7.01 x 10^-4^  (3, 8), p = 8.12 x 10^-4^  (4, 5), p = 1.00  (4, 6), p = 0.530  (4, 7), p = 0.275  (4, 8), p = 0.107  (5, 6), p = 1.00  (5, 7), p = 1.00  (5, 8), p = 1.00  (6, 7), p = 1.00  (6, 8), p = 1.00  (7, 8), p = 1.00 |
| **2g** | first 25% informed fish  last 25% informed fish | n = 94 sessions  n = 51 sessions | two-sided Mann-Whitney test | U = 2948, p = 0.0226 |
| **2i** | early informed fish | n = 153 sessions (early group)  n = 35 sessions (late group) | two-sided Mann-Whitney | U = 3165, p = 0.0935 |
| **2k** | late informed fish | n = 49 sessions (early group)  n = 139 sessions (late group) | two-sided Mann-Whitney | U = 4699.0, p = 7.88 x 10^-5^ |
| **2m** | not informed fish | n = 10 sessions (early group)  n = 33 sessions (late group) | two-sided Mann-Whitney | U = 255.0, p = 0.0100 |
| **2o** | late fish with none, 1-2, 3+ early neighbors | n = 13 sessions  n = 17 sessions  n = 19 sessions | Kruskal-Wallis test | H = 6.505, p = 0.0386 |
|  |  |  | post hoc Dunn's test with Bonferroni correction | (none, 1-2), p =0.460  (none, 3+), p = 0.0323  (1-2, 3+), p = 1.00 |
| **3c** | projection of virtual conspecifics animation | n = 14 fish (off)  n = 14 fish (on) | two-sided Wilcoxon signed-rank test | W = 1.0, p = 2.441 x 10^-4^ |
| **3g** | trial with X virtual fish escape | n=26 (1 fish)  n=26 (3 fish)  n=26 (5 fish) | two-sided Friedman chi square test | Q = 6.230, p = 0.044 |
|  |  |  | post hoc Dunn’s test with Holm correction | (1, 3), p = 0.690  (1, 5), p = 0.0283  (3, 5), p = 0.0560 |
| **4c** | average Z-scored response of cells during each stimulus type | n=30 sessions (from 12 fish) | Kruskal-Wallis test | H = 42.254, p = 6.67 x 10^-10^ |
|  |  |  | post hoc Dunn’s test with Bonferroni correction | (linear, BM), p = 2.09 x 10^-3^  (BM, BM-escp), p = 5.64 x 10^-3^  (linear, BM-escp), p = 2.43 x 10^-10^ |
| **4f** | average of cells peaked in 0-3, 3-6, 6-9 sec post stimulus onset | n=12 fish (BM-escape) | linear mixed effects model | (0-3, 3-6), p < 1.00 x 10^-10^  (3-6, 6-9), p = 2.43 x 10^-204^  (0-3, 6-9), p = 1.42 x 10^-78^ |
|  |  | n=12 fish (BM) | linear mixed effects model | (0-3, 3-6), p = 1.30 x 10^-264^  (0-3, 6-9), p = 4.77 x 10^-283^ |
|  |  | n=12 fish (Linear) | linear mixed effects model | (0-3, 3-6), p = 1.40 x 10^-161^  (0-3, 6-9), p = 2.81 x 10^-196^ |
| **5h** | Support vector model performance trained with real vs. bootstrapped data (over the 2 sec before escape or disappearance) | n=15 sessions (BM vs. LM) | one-sided T-test | t = 17.705, p = 4.831 x 10^-17^ |
|  |  | n=15 sessions (Escape vs. Disappear) | one-sided Mann-Whitney test | U = 25.0, p = 0.998 |
|  |  | n=15 sessions (Escape vs. Loom) | one-tailed T-test | t = -2.141, p = 0.979 |
|  |  | n=15 sessions  (Disappear vs. Loom) | one-tailed T-test | t = -3.790, p = 0.999 |
| **5i** | Support vector model performance trained with real vs. bootstrapped data (over the 2 sec after escape or disappearance) | n=15 sessions (BM vs. LM) | one-tailed T-test. | t = 16.274, p = 4.177 x 10^-16^ |
|  |  | n=15 sessions (Escape vs. Disappear) | one-tailed T-test | t = 0.140, p = 0.444. |
|  |  | n=15 sessions (Escape vs. Loom) | one-tailed T-test | t = 10.054, p = 4.276 x 10^-11^ |
|  |  | n=15 sessions  (Disappear vs. Loom) | one-tailed T-test | t = 14.067, p = 1.606 x 10^-14^ |
| **5m** | virtual conspecifics action following biological motion | n=39 (no action)  n=39 (escape)  n=41 (disappear) | Kruskal-Wallis test | H = 9.222, p = 0.0099 |
|  |  |  | post hoc Dunn’s test (sig. comparisons) | (no action, escape), p = 0.0131  (no action, disappear), p = 0.0376 |
| **5o** | virtual conspecifics action following linear motion | n=35 (no action)  n=36 (escape)  n=34 (disappear) | Kruskal-Wallis | H = 0.219, p = 0.895 |
| **ED 1d** | single fish  four-fish group | n = 18 sessions  n = 20 sessions | two-sided Mann-Whitney | U = 179.0, p = 0.988 |
| **ED 1f** | single fish  four-fish group | n = 15 sessions  n = 80 sessions | two-sided Mann-Whitney | U = 780.0, p = 0.0385 |
| **ED 1g** | single fish  four-fish group | n = 18 sessions  n = 20 sessions | two-sided T-test | t = -1.059, p = 0.296 |
| **ED 1j** | single fish (1)  two fish (2)  four fish (3)  eight fish (4) | n = 18 sessions (1)  n = 18 sessions (2)  n = 20 sessions (3)  n = 22 sessions (4) | One-way ANOVA | F = 1.866, p = 0.142 |
| **ED 1k** |  |  | Kruskal-Wallis test | H = 0.093, p = 0.992 |
| **ED 1l** |  |  | Kruskal-Wallis test | H = 8.810, p = 0.0319 |
|  |  |  | post hoc Dunn’s test with Bonferroni correction (sig. comparisons) | (1, 3), p=0.0220 |
| **ED 1m** |  |  | Kruskal-Wallis test | H = 1.928, p = 0.587 |
| **ED 2e** | single fish  four-fish group | n = 15 sessions  n = 15 sessions | two-sided T-test | t = -2.980, p = 0.0059 |
| **ED 2f** | single fish  four-fish group | n=15 sessions  n = 15 sessions | two-sided Mann-Whitney test | U = 60.0, p = 0.0310 |
| **ED 2h** | single fish  four-fish group | n = 14 sessions  n = 58 sessions | two-sided Mann-Whitney test | U = 540.0, p = 0.0369 |
| **ED 2j** | single fish  four-fish group | n=15 sessions  n = 15 sessions | two-sided Mann-Whitney | U = 180.0, p = 0.0054 |
| **ED 3b** | light condition | n = 15 sessions (light)  n = 13 sessions (dark) | two-sided Mann-Whitney test | U = 70.0, p = 0.213 |
| **ED 3c** |  |  | two-sided T-test | t = 0.827, p = 0.415 |
| **ED 3d** |  |  | two-sided Mann-Whitney test | U = 77.0, p = 0.356 |
| **ED 3e** |  |  | two-sided Mann-Whitney test | U = 109.0, p = 0.612 |
| **ED 3g** | light condition | n = 20 sessions (light)  n = 18 sessions (dark) | two-sided T-test | t = 1.522, p = 0.136 |
| **ED 3h** |  |  | two-sided Mann-Whitney test | U = 181.0, p = 0.988 |
| **ED 3i** |  |  | two-sided T-test | t = 1.856, p = 0.0716 |
| **ED 3j** |  |  | two-sided Mann-Whitney test | U = 188.0, p = 0.826 |
| **ED 3l** |  |  | two-sided T-test | t = 3.516, p = 0.00120 |
| **ED 4k** | number of early informed neighbors: 0-1 (1), 2 (2), 3 (3), 4 (4), 5 (5) | n = 15 sessions (1)  n = 15 sessions (2)  n = 7 sessions (3)  n = 4 sessions (4)  n = 6 sessions (5) | Kruskal-Wallis H-test | H = 13.636, p = 0.00855 |
|  |  |  | post hoc Dunn's test with Bonferroni correction | (1, 2), p = 0.551  (1, 3), p = 1.00  (1, 4), p = 0.0851  (1, 5), p = 0.303  (2, 3), p = 1.00  (2, 4), p = 1.00  (2, 5), p = 1.00  (3, 4), p = 0.488  (3, 5), p = 0.330  (4, 5), p = 1.00 |
| **ED 5e** | virtual escape  loom | n = 26 sessions  n = 41 sessions | Kruskal-Wallis test | H = 0.537, p = 0.463 |
| **ED 6q** | Support vector model performance trained with real vs. bootstrapped data (during 6 sec stimulus presentation) | n = 15 sessions (all stimulus) | two-sided T-test | t = 23.449, p = 6.012 x 10^-20^ |
|  |  | n = 15 sessions (BM vs. BM-escp) | two-sided T-test | t = 20.384, p = 2.461 x 10^-18^ |
|  |  | n = 15 sessions (neutral vs. dangerous) | two-sided T-test | t = 19.563, p = 7.234 x 10^-18^ |
|  |  | n = 15 sessions (BM vs. linear) | two-sided T-test | t = 27.534, p = 8.040 x 10^-22^ |
| **ED 7f** | social offset cells  general offset cells | n = 5237 cells  n = 2970 cells | linear mixed effects model analysis | (BM-escape), p = 1.453 x 10^-17^  (BM), p = 6.177 x 10^-6^  (linear), p = 2.474 x 10^-9^ |
| **ED 8b** | projection of virtual black dots | n = 9 fish (off)  n = 9 fish (on) | two-sided Wilcoxon signed-rank test | W = 2.0, p = 0.0117 |
|  | projection of virtual gray dots | n = 9 fish (off)  n = 9 fish (on) | two-sided Wilcoxon signed-rank test | W = 14.0, p = 0.359 |
| **ED 8f** | average Z-scored response of cells during each stimulus type | n = 28 sessions | One-way ANOVA | F = 5.88, p = 2.15 x 10^-4^ |
|  |  |  | post hoc Tukey’s HSD test (sig. comparisons) | (BM-disp, BM-loom), p = 0.0007  (BM-loom, Linear-disp), p = 0.0003 |
| **ED 8g** | ratio of responsive cells during each stimulus type | n = 28 sessions | One-way ANOVA | F = 1.357, p = 0.252 |
| **ED 8o** | virtual fish action | n = 39 (BM-no action)  n = 39 (BM-escape)  n =41 (BM-disappear)  n = 42 (BM-loom)  n =35 (LM-no action)  n = 36 (LM-escape)  n = 34 (LM-disappear) | Kruskal-Wallis test | H = 46.381, p=2.485 x 10^-8^ |
|  |  |  | post hoc Dunn’s test with a Holm multiple-testing correction (sig. comparisons) | (BM-no action, BM-loom), p = 5.164 x 10^-4^  (BM-escp, BM-loom), p = 3.026 x 10^-4^  (BM-disp, BM-loom), p = 0.0141  (LM-no action, BM-loom), p = 1.043 x 10^-7^  (LM-escp, BM-loom), p = 3.722 x 10^-4^  (LM-disp, BM-loom), p = 1.076 x 10^-6^ |
